# The Impact of CpG Island on Defining Transcriptional Activation of the Mouse L1 Retrotransposable Elements

**DOI:** 10.1371/journal.pone.0011353

**Published:** 2010-06-29

**Authors:** Sung-Hun Lee, Soo-Young Cho, M. Frances Shannon, Jun Fan, Danny Rangasamy

**Affiliations:** 1 The John Curtin School of Medical Research, Australian National University, Canberra, Australia; 2 Division of Molecular and Life Sciences, Hanyang University, Ansan, Republic of Korea; Georgia Institute of Technology, United States of America

## Abstract

**Background:**

L1 retrotransposable elements are potent insertional mutagens responsible for the generation of genomic variation and diversification of mammalian genomes, but reliable estimates of the numbers of actively transposing L1 elements are mostly nonexistent. While the human and mouse genomes contain comparable numbers of L1 elements, several phylogenetic and L1Xplore analyses in the mouse genome suggest that 1,500–3,000 active L1 elements currently exist and that they are still expanding in the genome. Conversely, the human genome contains only 150 active L1 elements. In addition, there is a discrepancy among the nature and number of mouse L1 elements in L1Xplore and the mouse genome browser at the UCSC and in the literature. To date, the reason why a high copy number of active L1 elements exist in the mouse genome but not in the human genome is unknown, as are the potential mechanisms that are responsible for transcriptional activation of mouse L1 elements.

**Methodology/Principal Findings:**

We analyzed the promoter sequences of the 1,501 potentially active mouse L1 elements retrieved from the GenBank and L1Xplore databases and evaluated their transcription factors binding sites and CpG content. To this end, we found that a substantial number of mouse L1 elements contain altered transcription factor YY1 binding sites on their promoter sequences that are required for transcriptional initiation, suggesting that only a half of L1 elements are capable of being transcriptionally active. Furthermore, we present experimental evidence that previously unreported CpG islands exist in the promoters of the most active T_F_ family of mouse L1 elements. The presence of sequence variations and polymorphisms in CpG islands of L1 promoters that arise from transition mutations indicates that CpG methylation could play a significant role in determining the activity of L1 elements in the mouse genome.

**Conclusions:**

A comprehensive analysis of mouse L1 promoters suggests that the number of transcriptionally active elements is significantly lower than the total number of full-length copies from the three active mouse L1 families. Like human L1 elements, the CpG islands and potentially the transcription factor YY1 binding sites are likely to be required for transcriptional initiation of mouse L1 elements.

## Introduction

The long interspersed nuclear element-1 (LINE-1 or L1) is the most prolific class of mammalian retrotransposable elements, comprising 21 to 19% of the human and mouse genomic sequences [1], [2]. L1 is an insertional mutagen capable of proliferating by its own retrotransposition. By providing the machinery necessary for the retrotransposition of Alu elements and processed pseudogenes [3], L1 acts as a major contributor to genome shaping. An L1 element can also modulate the expression of a given gene by contributing a source of transcriptional regulatory signals previously not present in the promoter of that gene [4]. In addition, L1 elements can shuffle exons throughout the genome creating new RNA products [5], further highlighting their evolutionary significance in genome function.

L1 elements share the same organization and conserved motifs between mammalian species; a single line of successive L1 elements has been amplified between 40 and 12 million years in the primate lineage leading to humans [6]. While the average retrotransposon activity of L1 has declined in humans, a significant number of L1 elements are still actively expanding in mammals and contributing to the dynamic nature of mammalian genomes. Both mouse and human genomes contain at least half a million copies of L1 elements scattered throughout the chromosomes. The majority of these elements are inactive because of truncation, mutation, and/or heavily rearranged sequences [7]. Less than 1% of L1 elements are full-length and classified as active or retrotransposition-competent. The full-length L1 is approximately 6 to 7 kb long and is composed of the 5′-untranslated region (5′-UTR), which harbors an internal promoter, two open reading frames (ORF1 and ORF2), and 3′ poly-A tail. ORF1 encodes a p40 protein with RNA-binding and chaperone activity while ORF2 encodes a protein of approximately 150 kDa with endonuclease and reverse transcriptase activities. Both ORF1 and ORF2 proteins are required for autonomous retrotransposition of L1 elements ([8] and references therein].

L1 is transcribed from its 5′-UTR internal promoter. Although mouse and human L1 ORFs are homologous, the promoter sequence contained within the 5′-UTR region shows no sequence homology between the two species [9]. In humans, the 5′-UTR is at least 910 base pairs (bp) long, with an internal promoter located within the first 155-bp and, with additional sequences for transcription-factor (YY1 and RUNX3)-binding sites and CpG dinucleotides are necessary for L1 transcription [10], [11], [12]. In contrast, the 5′UTR sequence of the mouse L1 contains tandem repeats of approximately 200-bp monomers that functions as a promoter [13], [14]. Increasing the number of monomers is reported to increase the level of promoter activity [15]. However, little is known about transcription factor binding sites or CpG dinucleotides within or near the 5′-UTR region that might regulate mouse L1 transcription. Previous phylogenetic analyses suggest that three mouse L1 subfamilies (T_F_, G_F_, and A distinguished by their monomer sequences; younger T_F_ <G_F_ <A older) exist in the mouse genome and are active [14]. Combined, these three L1 subfamilies make up 3,000 active L1 elements. This greatly exceeds the estimated number of potentially active human L1 elements. However, until now, it has been unclear why such a high copy number of the active L1 elements exist in the mouse genome and what mechanisms are responsible for the transcriptional activation of mouse elements.

A recent release of the L1Xplorer database [16] predicted that there are 151 full-length, active L1 elements potentially capable of retrotransposition activity in the human genome (Ensembl version 38.36). In contrast, the mouse genome (Ensembl version 24.33) is predicted to contain at least 1,501 potentially active L1 elements, ten times higher than in humans. At present, it is not known whether all the predicted mouse L1 elements retain their ability to be expressed and retrotransposed into the genome, or if only a subset of elements is responsible for the high density of L1 elements in the mouse genome. The molecular differences between the elements also remain unclear. Given that the retrotransposition of L1 elements often disrupts genes and causes several genetic diseases [17], recent L1 research has focused on the identification of currently active L1 loci in the genome.

Recent promoter analysis shows that both mouse and human L1 elements contain a putative E2F/Rb binding site (5′-G/CG/CCGGC-3′) within their 5′-UTR promoters [18]. Because the E2F/Rb protein complex binds to CpG islands in several genes and regulates gene expression [19], we hypothesized that, as for human L1, the presence of CpG-rich sequences in mouse 5′-UTR promoters might play a role in the regulation of mouse L1 expression. To explore this hypothesis, we performed comparative analyses of the 5′UTR sequences of mouse elements, particularly focusing on all active mouse L1 elements retrieved from the GenBank and L1Xplore databases, and analyzed the transcription factor binding sites and CpG dinucleotides within the 5′-UTR sequences. Here we show that only a half of mouse L1 elements (approximately 710) is capable of activity, by measuring the promoter activity using luciferase reporter constructs–a significantly lower fraction than we initially predicted. Of the 710 mouse elements, only 124 contain previously unreported CpG islands in their promoters that showed a high level of promoter activity. Unlike humans, none of the mouse L1 promoters contains RUNX3 transcription factor binding sites. In addition, we found that approximately 754 mouse L1 elements contain altered YY1 transcription factor binding sites in their promoter sequences that may be necessary for L1 expression in the mouse genome.

## Results and Discussion

### Sequence analysis of intact L1 elements in the mouse genome

**Figure 1:**
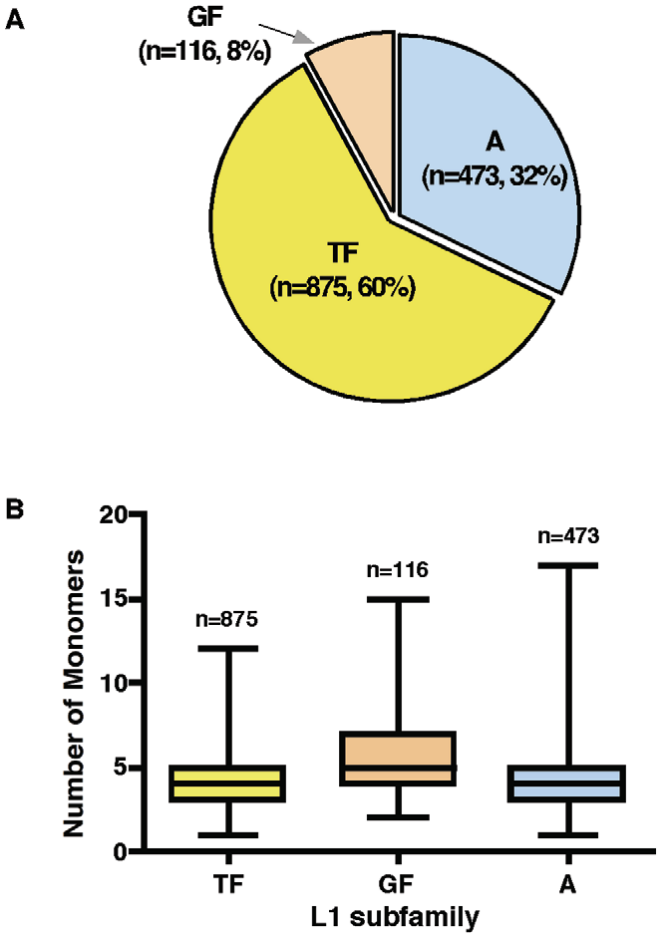
Identification of potentially active L1 elements in the mouse genome sequence (UCSC mm8) based on their monomer sequences of subfamilies. (**A**) A total of 1,464 active L1 elements were analyzed and the distribution of three main L1 subfamilies T_F_, A, and G_F_ is shown. The T_F_ subfamily is considered as the youngest active elements. Subfamily F and unclassified L1 elements were removed from the data analysis. (**B**) The average number of monomers present in each subfamily of L1 5′-UTR promoters. Boxplots show the average length of monomers and standard deviation representing the varying length of monomers. The longest monomer in the T_F_ subfamily is 12, followed by 15 and 17 monomers for G_F_ and A subfamilies, respectively.

To identify and characterize potentially active L1 elements in the mouse genome, we utilized the non-redundant L1Xplorer (Ens24.33) database to ensure that our dataset contained only full-length, intact L1 elements including a 5′-UTR promoter, two open reading frames (ORF1 and ORF2), and 3′ poly-A tail sequences. Interrogating this database revealed the presence of 1,464 potentially active L1 elements residing in the mouse haploid genome (version mm5, NCBIm33). This figure supports previous estimates that the diploid mouse genome consists of approximately 3,000 potentially active L1 elements (2*1464 = 2928) [14], [20]. Unlike that of the human L1, the mouse 5′-UTR L1 promoter is a bipartite sequence in which tandem repeats of monomers are situated upstream of non-monomer sequences. By linking the 5′-UTR sequences to reporter genes, it has been shown that the monomers possess promoter activity [15]. The mouse genome contains several subfamilies of L1 elements, defined by differences in their monomer sequences. The recently evolved T_F_ subfamily, together with the A and G_F_ subfamilies are considered active elements [21]. We first estimated the number of potentially active L1 elements by combining the number of L1 elements in these three subfamilies (**Figure 1A**). In our data analysis, the most abundant L1 is the youngest T_F_ subfamily, which contains 875 elements, followed by 473 and 116 elements for the A and G_F_ subfamilies, respectively. In addition, the mouse genome also contains 3 L1 elements of the oldest F subfamily (with highly truncated 5′-UTR promoters) and 34 unclassified L1 elements. These elements may not be currently active in the genome because their 5′-UTR sequences are heavily rearranged and divergent from one another beyond recognition.

Because L1 promoter activity is thought to be proportional to the number of the monomers in that promoter, we next determined the average number of monomers present in each of the mouse L1's subfamilies. By characterizing the 5′-UTR promoters of the 1,464 potentially active L1 elements, we found an average of 5.6 monomers in the G_F_ subfamily, followed by 4.26 and 4.1 monomers for A and T_F_ subfamilies, respectively (**Figure 1B** and **Table S1**). Given that only two monomers are sufficient for L1 promoter activity [21], the presence of such a large number of intact monomers suggests that the majority of these elements may be active within the mouse genome.

### Genomic distribution of potentially active L1 elements

L1 is an insertional mutagen capable of disrupting gene function as well as altering the regulatory properties and expression patterns of neighbouring genes. In humans, the highly active L1 elements residing within or close to known or predicted genes can affect the expression of nearby genes [22], suggesting a correlation between the localization of active L1 elements and nearby gene expression. Thus, identifying the genomic distribution of potentially active mouse L1 elements and their neighboring genes could be biologically informative. To accomplish this, we downloaded the entire list of the 1,464 intact L1 elements from the L1Xplorer database (Ens24.33) and aligned them with the full UCSC mouse genome (freeze March 2006, NCBI Build 35, UCSC mm8,) using the BLAST-like alignment tool (BLAT) to generate the genomic location of all active mouse L1 elements. Because the intact L1 elements were originally predicted by L1Xplorer using an earlier version of the mouse genome, mm5 [16], we realigned all the L1 element sequences to the mm8 version of the mouse genome assembly. This allowed us to avoid any potential discrepancy caused by varying genomic distributions in earlier versions of the mouse genome assembly.

**Figure 2:**
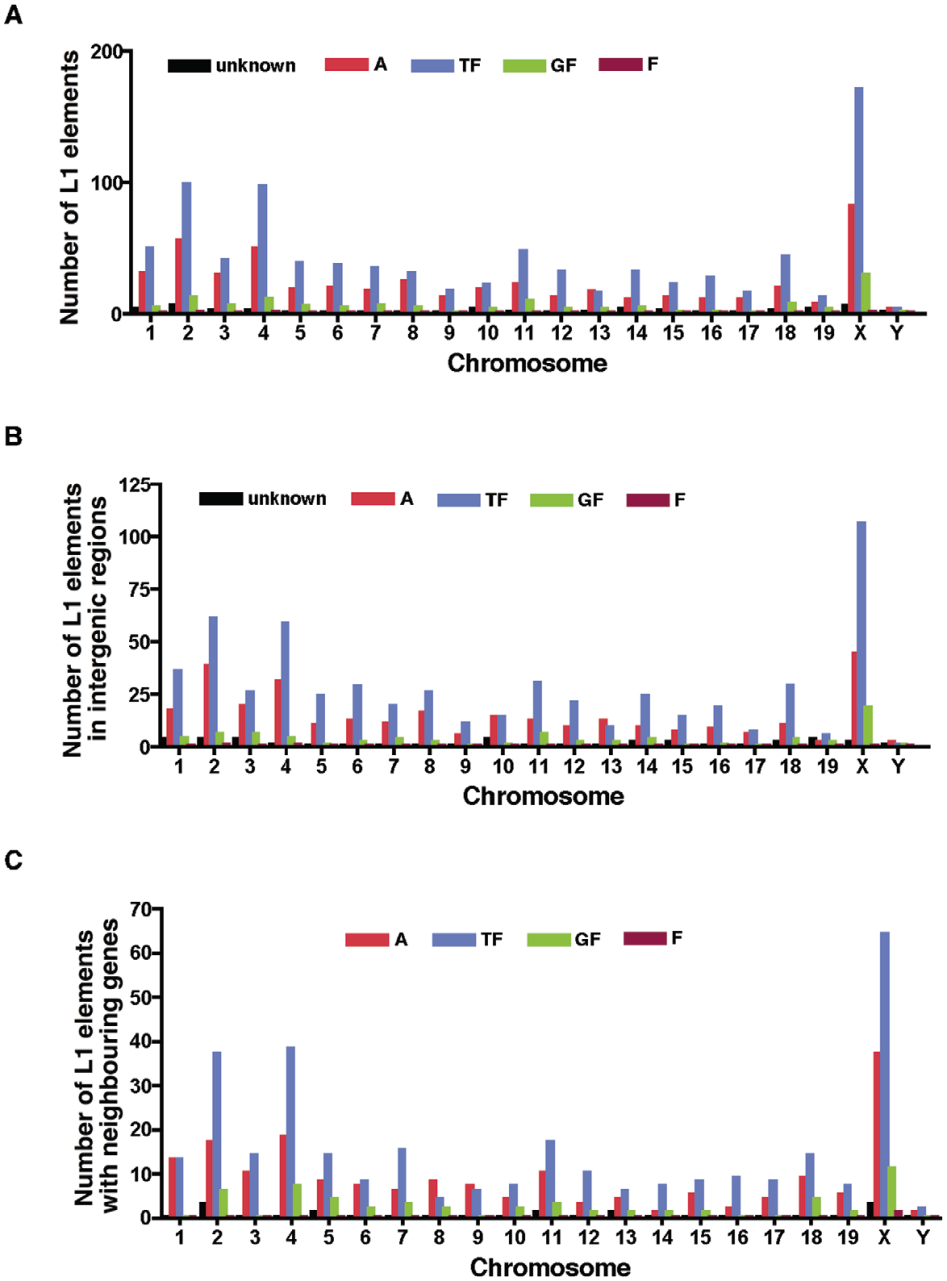
Genomic distributions of L1 elements and subfamilies. (**A**) Number of potentially active L1 elements (n = 1,464) residing on individual mouse chromosomes. (**B**) Distribution profiles of L1 elements among subfamilies (n = 953) located at intergenic regions, and (**C**) gene-rich regions of the mouse chromosomes having annotated neighboring genes in 100-kb windows surrounding the L1 elements (n = 546). The frequency of L1 elements per chromosome was calculated by Chi-square test for trend (χ^2^ = 6.688, dt = 1, *p* = 0.0097).

To identify the density of L1 elements within and near genes, we initially determined how many L1 elements are present on each mouse chromosome. As expected, L1 elements are present on every chromosome and the distributions of L1 subfamilies are shown in **Figure 2A**. Analysing this dataset shows that there is a weak positive correlation between the size of the chromosome and the density of L1 elements on each chromosome (Pearson's correlation coefficient r = 0.588, R^2^ = 0.346, *p* (two-tailed)  = 0.005). Statistical analysis of the distribution of L1 elements on the chromosomes is presented in **Table S2**. Although L1 elements are found in all the chromosomes, their abundance varies considerably. The highest density of potentially active L1 elements resides in the X-chromosome (average ratio 1.81%), whereas the autosomes contain lower densities of L1 elements (with ratios between 0.24% and 1.07%). Consistent with other species, the X-chromosome in mice seems disproportionately enriched for intact L1 elements as compared to autosomes, indicating an L1 insertional bias; however, natural selection may also be the driving force. As suggested by other studies in humans, and *Drosophila* [23], [24], the underrepresentation of L1 in autosomes could reflect strong selection against L1 insertions. Although L1 density shows weak correlation with chromosomes size, the distribution profiles suggest that L1 elements perhaps cluster preferentially in certain genomic regions and are similar to the clustering of the human Ta-1 elements [25]. The clustering of L1 elements on the X-chromosome has been previously reported to serve as a ‘booster’ signal to promote the spread of Xist RNAs for X-inactivation of the genes [26]. Interestingly, genes subjected to monoallelic expression, such as random monoallelic genes and imprinted genes, are also flanked by high densities of intact L1 elements [27], [28]. In contrast, biallelically expressed genes contain a lower density of L1 elements, suggesting that L1 could act as a regulator of neighboring genes.

### Distributions of intact L1 elements with respect to genes

To explore the relationship between the presence of L1 elements and neighboring gene expression, we next looked in more detail at the distribution of L1 elements by locating all elements in the mouse genome relative to annotated genes. Using a Perl script, we estimated how many neighboring genes exist within flanking sequences 100-kb in either direction of potentially active L1 elements. Surprisingly, we found that 64% (n = 953) of the intact L1 elements occupy intergenic regions within the 100-kb regions; that is, they are not within exons, introns, or untranslated regions (**Figure 2B** **and Table S3**). The oldest inactive F subfamily and unclassified L1 elements were also found in intergenic regions. Analyzing these 953 L1 element locations and nucleotide sequences around the L1 insertion sites revealed a significantly high AT-rich content (mean AT = 61.3%; the average AT content of the genome  = 58.6%, *t*-test, p<0.00001). This could partly explain why the intergenic regions of the mouse genome contain lower gene density and higher accumulation of L1 elements that prefer AT-rich regions for their insertion. Activation of L1 elements from the AT-rich intergenic regions might lead to accumulation of L1 elements within this gene-poor region of the genome, but the probability of L1 elements interfering with genes would be quite low. Because the intergenic genomic sequences are mostly bundled into repressive heterochromatin [29], it is reasonable to expect that, with some exceptions, these L1 elements might not be in a fully active state.

Remarkably, for up to 36% of T_F_, G_F_, and A (307, 49, and 180, respectively) subfamilies there are neighboring genes within a 100-kb window of the L1 elements; their chromosomal distributions are indicated in **Figure 2C**. The frequency of L1 elements per chromosome was calculated by Chi-square test for trend (χ^2^ = 6.688, dt = 1, *p* = 0.009). Consistent with previous estimates [28], the X-chromosome has the highest frequency of L1 elements in proximity to genes (116 compared with the expected frequency of 34.19), followed by chromosomes 2, and 4. Surprisingly, chromosomes 9, 10, and 14 have lower L1 frequencies (less than 13) than other autosomes (**Table S4**). This uneven distribution of L1 elements in the proximity of genes prompted us to ask whether the trend in L1 frequency with respect to genes is due to surrounding GC content, or whether genes exert independent effects on the distribution of L1 density. Analysis of the GC-content across 20-kb surrounding regions of L1 elements did not reveal high significant difference from what would be expected by chance in regions surrounded by genes (40.08% mean GC content compared with 38.3% GC content of intergenic regions; *t*-test, p<0.0001). At present, it is unclear why some gene-rich regions, but not others are prone to L1 element insertion.

One possible hypothesis is that the local chromatin environment and associated DNA sequences might influence the density of L1 elements in some genomic regions of mouse chromosomes. Recently, Graham and Boissinot proposed that the transcriptional status at the insertional site could favour the accumulation of L1 inserts near genes [30]. This can be mechanistically explained that transcription is associated with a decondensation of chromatin, which increases the rate of L1 insertion by rendering DNA accessible to the transcriptional machinery and potentially also to enzymes involved in retrotransposition. Although the existence of such insertional sites is yet to be proven in mice, some L1 preferential sites were identified in the vicinity of developmentally regulated genes active in testis and during embryogenesis [31]. In addition, studies on L1 retrotransposition reported a significant number of newly transposed L1 elements choose their insertional places within or near neuron-specific genes [32], which are transcriptionally active during the process of neurogenesis. These and other studies support the hypothesis that a relationship might exist between the transcriptional activation of tissue-specific genes and accumulation of L1 insertions in certain genomic regions of mammals.

**Figure 3:**
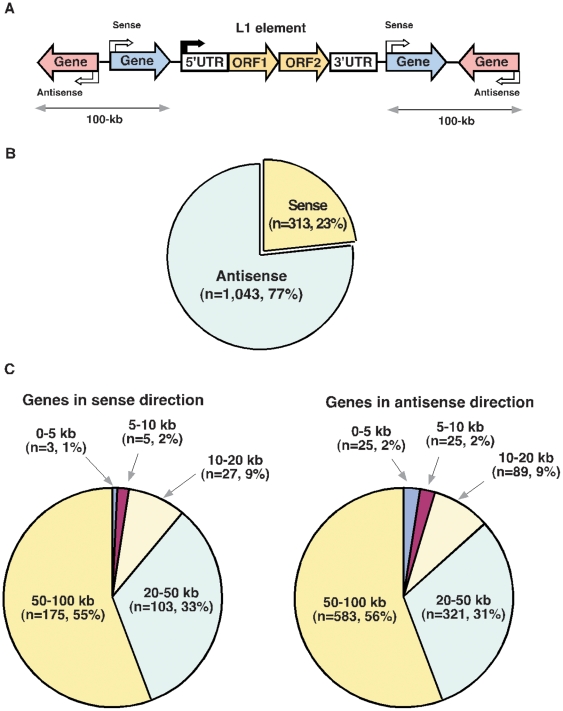
Distributions of annotated neighboring genes. (**A**) Schematic displays of neighboring genes around the 100-kb flanking sequences in both sense and antisense directions of L1 elements are shown (n = 1,356, see **Table S5**). (**B**) Proportion of annotated neighboring genes in the sense or antisense orientation with respect to L1 elements. (**C**) Distribution profiles of neighboring genes located in terms of distance from the L1 elements (0–5 kb, 5–10 kb, 10–20 kb, 20–50 kb, and 50–100 kb) in sense (left panel) and antisense (right panel) orientations. ‘n’ represents the number of L1 elements.

To investigate whether such a relationship exists in the genomic distribution of mouse L1 elements, we determined the orientation and type of the RefSeq genes located upstream and downstream of each L1 subfamily (**Figure 3A and 3B**). A total of 1,356 genes were identified in the 100-kb flanking sequences of L1 elements (**Table S5**). The proportion of genes oriented in the opposite (antisense) transcriptional direction with respect to L1 elements was roughly three times greater than the same (sense) orientation over the 100-kb regions (1,043 and 313 genes, respectively). Of the genes located within 100-kb of an L1 element, the majority (87%, or 1,182 genes) are located more than 20-kb from an L1 element, suggesting that L1 elements are preferentially landed at significant distances from genes (**Figure 3C**). Notably, many of these L1 elements belong to the newly evolved, highly active T_F_ subfamily (307/536, 57%), followed by the A subfamily (180/536, 34%). The overrepresentation of T_F_ L1 elements indicates that these regions are probably prone to L1 integration. Because L1 insertions in these regions are generally more than 20 kb away from genes, we hypothesize that negative selection pressure against deleterious effects of L1 insertion could also play a role in the genomic distribution of L1 elements. Surprisingly, many of the genes identified within 100-kb of an L1 element are tissue-specific genes, expressed mainly in testis, placenta, and brain tissues, as well as in neural progenitor cells (**Table S5 and S6**).

The relationship between mouse L1 elements and the location of nearby genes is not well understood. The L1 elements are generally more active in germ-line cells than in somatic cells. Recently, the Jordan group hypothesized that L1 elements tend to be enriched far from transcriptional start sites of genes and depending upon the kind of repeat, some may recruit epigenetic factors to function as gene regulators to nearby genes by opening or closing the local chromatin to transcription factors [33], [34]. Although this hypothesis is yet to be tested in mouse genome, the evidence presented in other organisms such as *Drosophila*, *Arabidopsis,* and *S. pombe* suggest that the local chromatin environment can be influenced by L1 insertions and that can spread to not only nearby genes but also over long genomic distances [[35] and references therein]. Consistent with this view, a recent study in mouse ES cells using a whole genome ChIP-seq analysis shows that L1 elements and their flanking regions are indeed enriched with specific repressive histone modifications that distinguish LINE-rich chromatin domains from other gene-rich domains [36]. It is, thus, possible that some of the tissue-specific genes residing near the L1 elements are likely to be influenced by the local chromatin or epigenetic nature of L1 elements.

Intriguingly, we found that three genes (1 olfactory gene, 1 neuron receptor, and 1 EST) reside within a 5-kb of an L1 element and are oriented in the same transcriptional direction as the L1 element (**Table S6**). The gene expression and functional genomics dataset (www.ebi.ac.uk/microarray-as/ae/) suggests that these genes are expressed exclusively in testis and brain during development. Because of the presence of potentially active L1 elements near the genes, it is tempting to speculate that the activity of L1 elements may affect the expression patterns of these developmentally transcribed genes. As demonstrated by human transcriptome studies [37], L1 insertions upstream of genes most likely decrease the transcription of nearby genes by disrupting regulatory elements or by functioning as epigenetic regulators. Although the exact mechanisms by which L1 elements affect gene expression are poorly understood, the process could possibly involve an alteration to the methylation status of L1 elements, similar to *Arabidopsis* transposons in which the expression of the flowering-time gene FWA is affected by the methylation status of nearby transposable elements [38]. Taken together, these studies suggest that L1 preferentially inserts near genes that are expressed during development and that L1 might possibly act as a regulator in the mouse genome.

### Transcriptional analysis of potentially active L1 elements

Unlike human L1 elements, mouse L1 elements contain multiple copies of monomers in their 5′-UTR regions. Each monomer functions as a core promoter, increasing L1 promoter activity in an additive manner; i.e. when the number of monomers increases, L1 promoter activity increases. A previous study of mouse L1 promoters proposed that a minimum of two monomers were required for efficient promoter activity [15]. Although the structure and organization of the monomers are well understood, the regulatory mechanisms responsible for monomer activity remain largely unclear. The monomer sequence is believed to contain several different transcription factor binding sites and other regulatory element binding sites that are necessary for tissue-specific activation of the L1 element. In human L1 promoters, because of the lack of a TATA box, the YY1-binding site is required for transcriptional initiation of L1 within the 5′-UTR [9], [12]. YY1 is a zinc finger protein that can function either as a transcriptional repressor or an initiator depending upon its interaction with other transcription factors such as TBP, TAFs, TFIIB, and Sp1 [39]. YY1 can also act as a mediator to recruit the Polycomb group proteins, Suz12 and DNA methyltransferase, to participate in the gene silencing process [40]. In addition, other transcription factor binding sites such as the RUNX3 also play a regulatory function in human L1 elements. Like humans, mouse L1 also has a TATA-less promoter that might require transcription factors binding motifs for its transcriptional initiation. Thus, identifying the transcription factor binding sites such as the RUNX3 and YY1 within the monomer region would presumably allow us to predict if the mouse L1 elements residing near genes are active and thus have effects on neighboring genes.

**Figure 4:**
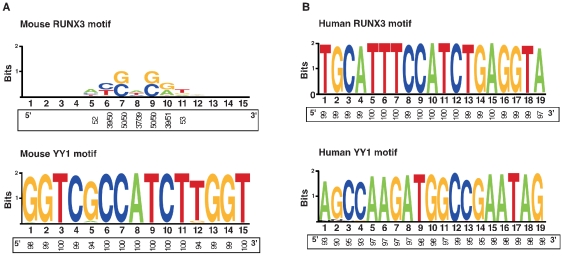
Characterization of the RUNX3 and YY1 transcription binding sites in both mouse and human L1 promoters. (**A**) Graphical representations of the mouse RUNX3 (Top panel) and YY1 (Bottom panel) sequence patterns within a sequence alignment of 819 T_F_ elements containing the 3,152 YY1 binding sites. The height of each stack indicates the sequence conservation (measured in bits) and the relative frequency of the nucleotides is shown on the x-axis. Note: RUNX3 motifs in mouse L1 elements are highly mutated and degenerated. (**B**) Graphical representations of the human RUNX3 (Top panel) and YY1 (Bottom panel) sequence patterns within a sequence alignment of 150 L1 elements containing both the RUNX3 and YY1 binding sites. The relative frequency of each nucleotide within the motifs is shown on the x-axis.

To analyse transcription factor binding motifs, we extracted all the L1 promoter sequences from 1,464 potentially active T_F_, G_F_, and A subfamilies and divided them into two categories based on the presence or absence of neighboring genes (i.e. “with neighboring genes”, or “intergenic L1 elements”, respectively). Using the TRANSFAC database, we searched the repeating monomer region of the 953 L1 promoters representing intergenic L1 elements and the 546 L1 promoters representing L1 elements with neighboring genes for presence of the conserved RUNX3 and YY1 binding sites. A previous study in human L1 promoters identified a potential RUNX3-binding site (5′-TGCATTTCCATCTGAGGTA-3′) starting at base pair +806 to +824 upstream of ATG start site [11], [12]. The mutations in the RUNX3 motifs have been shown to markedly disrupt promoter activity, suggesting a role for RUNX3 in activation of the human L1 expression. Likewise, to identify whether the similar sort of RUNX3 motifs exist in the mouse L1 promoters, we initially searched for RUNX3 or RUNX3-related sequences by scanning against the entire mouse L1 promoter sequences. Surprisingly, none of the mouse L1 promoters contains intact RUNX3 or RUNX3-related sequences. All the predicted RUNX3 binding motifs are heavily mutated or degenerated beyond recognition (**Figure 4A**), suggesting that the RUNX3 transcription factor may not be involved in the activation of the mouse L1 promoter.

**Figure 5:**
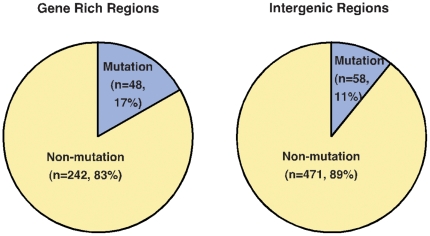
The percentage of mouse L1 promoters found to contain mutations at the YY1 consensus motifs in neighboring genes (left panel; n = 290) and intergenic regions (right panel; n  = 529 L1 elements).

Interestingly, the TRANSFEC analysis of mouse L1 promoters showed potential YY1 binding sites within their monomers. The consensus-binding site for the transcription factor YY1 was identified as 5′-GGTCGCCATCTTGGT-3′. Comparative analysis of both the human and mouse L1 promoters containing putative RUNX3 and YY1 binding sites are shown in **Figure 4A and 4B**. Given that at least two monomers are required for mouse L1 promoter activity, we selected only those promoters containing two or more monomers for the YY1 motif analysis (**Table S7**). There are 819 promoters in the T_F_ subfamily followed by 427 in the A subfamily and 113 in the G_F_ subfamily matching this criteria. Of the 819 T_F_ promoters, 290 have neighboring genes and the remaining 529 represent intergenic regions. By aligning the sequences of T_F_ monomers with the consensus sequence of the YY1 motifs, we found that 17% (48/290) of T_F_ elements from gene-rich regions and 11% of T_F_ elements (58/529) from intergenic regions contain mutations within the putative YY1 binding site of a ‘minimal’ promoter that is composed of only two monomers (**Figure 5**). We assume that these mutated or degenerated YY1 motifs (defined as differing by >20% from the functional motif) may not have promoter activity (**Table S7**). Consistent with this assumption, previous studies of human L1 promoters also identified the YY1-binding site as an important sequence for L1 expression and found that mutations in the putative YY1 motif markedly disrupt promoter activity [11], indicating that functionally intact YY1 motifs are required for transcriptional initiation of human L1 elements. Nevertheless, the data presented in this study suggest that 83% of T_F_ promoters (242/290) reside close to RefSeq genes and contain potentially functional YY1 motifs in an array of more than two monomers on the same promoter, indicating that these elements are potentially capable of influencing the expression of neighboring genes.

Unexpectedly, most L1 promoters in the A and G_F_ subfamilies that have neighboring genes also contain highly degenerated YY1 motifs in all monomers of the promoters (differing by up to 55% from the consensus sequences); we therefore omitted these elements from further analysis. Only a small subset of A and G_F_ elements from intergenic regions (24 elements) still contain putative YY1 motifs, suggesting that only these are capable of activity. This may explain why functionally active A and G_F_ elements are rare in the mouse genome. Consistent with this observation, no G_F_ subfamily of L1 elements have been detected in any *Mus spretus* or *Mus musculus* genomes except the genome of laboratory strain 129/Sv [14]. Based on cloning a limited number (<10) of A and G_F_ subfamily L1 elements from an embryonic stem cell library of strain 129/Sv, it was previously estimated that 900 A and 400 G_F_ elements are active in the mouse genome [14]. However, these experiments were done with altered L1 elements–they were put under the transcriptional control of a CMV promoter rather than being regulated by their own monomers. Because the L1 endogenous promoters did not drive transcription, the data presented in that study might not accurately reflect the number of active A and G_F_ elements in the mouse genome. Nevertheless, at least three L1 elements belonging to the A-type subfamily have recently been shown to be active in mouse vascular smooth muscle cells [41], indicating that some A and G_F_ subfamily L1 elements could be still active in some specialized cell types. Taken together, the data presented in our study suggest that the vast majority of T_F_ promoters contain YY1 motifs within their monomer regions and that this may have implications for transcriptional initiation of the mouse L1 elements.

### YY1 motifs overlap with CpG dinucleotides

The mammalian L1 regulatory sequences, though not homologous, share several features with viral and housekeeping promoters; they contain CpG islands and lack the traditional TATA boxes found in cell-specific PolII promoters [9]. For many housekeeping genes, the presence of CpG islands in their promoter is important for transcriptional regulation; the CpG islands must be unmethylated for gene activation to occur. Conversely, the methylation of CpG sequence can lead to the permanent silencing of genes. Several lines of evidence show that the L1 promoters of humans, chimpanzees, and rats are all GC rich and contain CpG islands in their promoters [42], [43]. Most of these elements are methylated and thus transcriptionally inactive, suggesting that CpG methylation is a mechanism to repress L1 expression in mammalian genomes. However, to date, little is known about the presence of CpG islands in the mouse L1 promoter. This prompted us to investigate whether mouse L1 elements contain any CpG islands in their promoters and, if so, whether any correlation exists between the presence of CpG islands and L1 expression in the context of neighboring genes.

**Figure 6:**
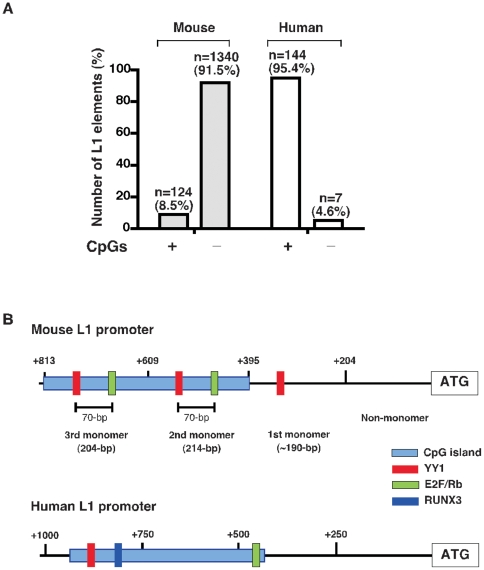
Characterization of the CpG islands in L1 promoter regions. (**A**) Comparative analysis of CpG island distributions in mouse and human L1 elements. ‘n’ represents the number of L1 elements. (**B**) Schematic representations of mouse and human L1 promoter regions. Vertical line above indicates the position and numbering of monomers; the box represents the transcription factors binding sites, YY1 (red), E2F (green), and RUNX3 (blue). The corresponding position of the CpG island in relation to start site ATG is illustrated by the light blue box.

To investigate this, we extracted the sequences of the 1,464 L1 promoters in the mouse genome and searched for CpG Islands using the EMBOSS CpGPlot analysis. CpG islands were defined as DNA sequences longer than 200 bp with >50% GC content and an observed/expected presence of CpG >0.6. By analysing the entire promoter sequences, we identified 124 mouse L1 elements that contain CpG islands in their promoter regions. This represents ∼8.5% of the estimated genomic copies of L1 in the mouse genome, much lower than that of the human and rat L1 elements where the majority contain CpG islands (**Figure 6A**). Analyzing each monomer sequence of these CpG-island-containing L1 promoters revealed that all the monomers are >65% GC-rich and contain sufficient CpG dinucleotides (roughly 16 CpGs) to qualify as CpG islands. The size of the CpG islands varies from 202–885 bp between L1 elements, with an average length of 302 bp, located mainly in the monomers 2 and 3 but not in the monomer 1 or non-monomeric regions (**Figure 6B**). Interestingly, most of the YY1 binding sites are located within these CpG islands, similar to their localization in human L1 promoters. In addition, we also found E2F/Rb binding sites (5′-TTTG/CG/CCGC-3′) within the CpG islands about 70-bp downstream from the YY1 motifs. A recent study in human and mouse L1 elements suggests that E2F/Rb motifs could regulate the transcriptional activation of L1 elements by interacting with histone deacetylases (HDACs) and other repressive histone modifications [18]. Given that both YY1 and E2F binding site motifs are located only in CpG-rich monomers such as monomer 2 or 3, but not in the CpG-poor monomer 1 region, the CpG islands could probably influence the functions of the YY1 and E2F motifs in transcriptional initiation of mouse L1 elements. This may, at least in part, explain why functionally active L1 elements require at least two monomers for promoter activity, as reported previously [15].

**Figure 7:**
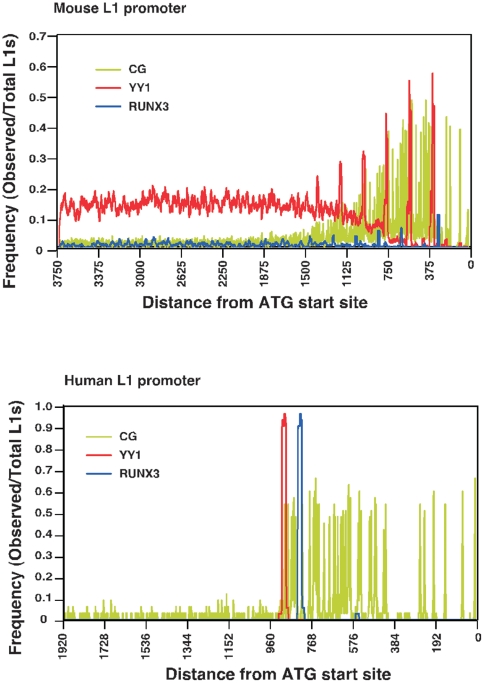
Positional distribution of CpG dinucleotides, the RUNX3 and YY1 motifs relative to start site ATG. The frequency of YY1 and RUNX3 co-occurrence with CpGs was analyzed in the 124 mouse L1 promoters (Top panel) and in the 144 human L1 promoter sequences (Bottom panel).

Intriguingly, we noticed that the YY1 binding site motifs in all the mouse L1 promoters show some level of conservation or positional specificity with respect to the ATG start site: the probability of each YY1 motifs existence peaked between 324–341 bp upstream from the ATG start site (**Figure 7**). To evaluate the positional specificity of YY1 motifs within CpG islands, we used a position frequency matrix (PFM), which allow us to calculate the nucleotide frequencies at each position of the YY1 motif embedded in promoter sequences [44]. For comparison, we also analyzed the 144 human L1 promoters. Comparative analysis of both mouse and human L1 promoters and the positional distribution of YY1 motifs relative to the CpG dinucleotides are shown in **Figure 7**. Analysis of the co-occurrence of YY1 motifs with CpG islands of the 124 mouse L1 promoter sequences shows that YY1 motifs have a significantly higher-than-expected co-occurrence frequency with CpG islands (∼60%), much similar to human L1 promoters (**Figure 7**). In contrast, for the 1,340 non-CpG-island-containing mouse promoters, we did not observe any co-occurrence frequency of YY1 motifs with CpG islands (data not shown). These differential levels of YY1 and CpG islands co-occurrence within the monomers of mouse L1 elements suggest that CpG islands are capable of influencing the ability of YY1 motifs to act in L1 transcriptional initiation.

**Figure 8:**
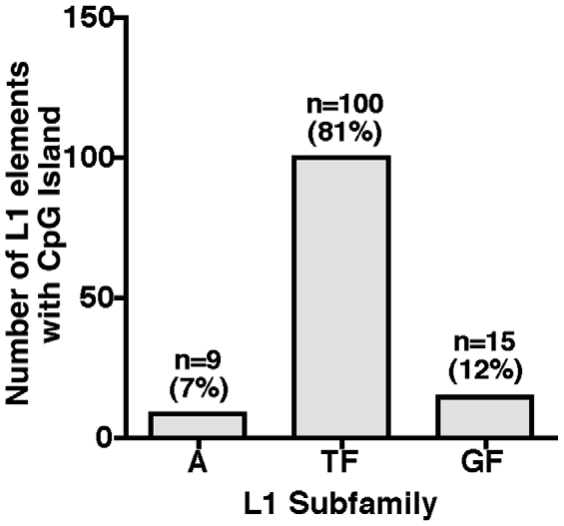
Distribution of CpG islands among the mouse L1s subfamilies (n = 124 L1 promoters).

**Figure 9:**
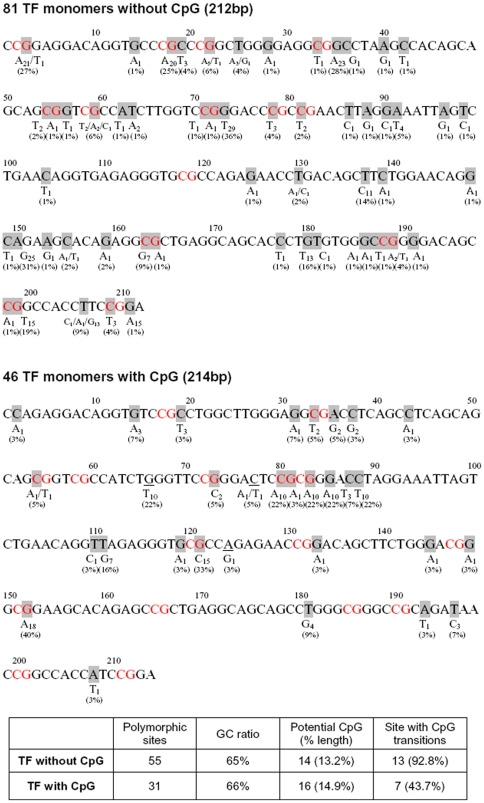
Analysis of polymorphism and CpG transitions of T_F_ monomers. The number and percentage of mismatches, CpG island length, and CpG transition sites were analyzed in T_F_ monomers containing or lacking a CpG island.

Remarkably, 81% of CpG islands are present in the highly active, young T_F_ subfamily (100/124), followed by 12% (15/124) and 7% (9/124) in the older G_F_ and A-subfamilies, respectively (**Figure 8**). Two major conclusions, which are typical of the mammalian L1 elements, are apparent: First, in mammals, CpG is a preferred site of cytosine methylation and the methylated cytosine over time is deaminated at a high frequency to form TpG (or CpA), resulting in loss of CpG islands. It has been recently reported that the older L1 families in humans and chimpanzees contain fewer CpG islands than the younger L1 families and the CpGs missing from the older L1 families are always compensated by gain in TpGs or CpAs mutations [45]. Because the mutation rates vary between families of L1 elements, some CpG islands disappear faster in the older families of mouse L1 elements such as the G_F_ and A subfamilies than in the younger T_F_ family. Second, out of a total 875 T_F_ promoters, we found that 100 have retained their CpG islands while the remaining 775 T_F_ have not. Analyzing the polymorphism and CpG transitions of T_F_ monomers containing with or without a CpG island (**Figure 9**), we observed that many of the differences are transition changes (C to T or G to A), which are the most common found in CpG island mutations. Overall, 71% of the CpG island mutations found in one non-CpG T_F_ monomers were also found in another. Taken together, the data presented in our study shows that the CpG islands are present in the T_F_ subfamily, making these promoters more likely to be influenced by DNA methylation, similar to L1 elements in humans. Given that the presence of CpG-rich L1 elements close to a number of tissue-specific genes, for instance, testis-specific gene (RefSeq NM_001004174) within a distance of 5 kb, it is tempting to speculate that the methylation status of L1 CpG islands might be associated with expression of developmentally transcribed genes during the process of cellular development.

### Biological functions of predicted CpG islands

CpG islands are selectively associated with the regulatory regions of L1 elements and are generally methylated in somatic cells of humans and rats. In fact, their methylation status has been reported to prevent constitutive expression of L1 elements [43]. The role of CpG islands in the expression of mouse L1 elements is unknown, but theoretically, the presence of a CpG island in an L1 element could indicate that the L1 promoter might become methylated, leading to suppression of L1 promoter activity. By contrast, CpG islands may be required for transcription factor binding sites, thus the presence of CpGs could increase promoter activity [15]. To determine which of these opposite effects play a relevant role in mouse L1 expression, we performed promoter analysis of CpG and non-CpG containing T_F_ L1 elements.

**Figure 10:**
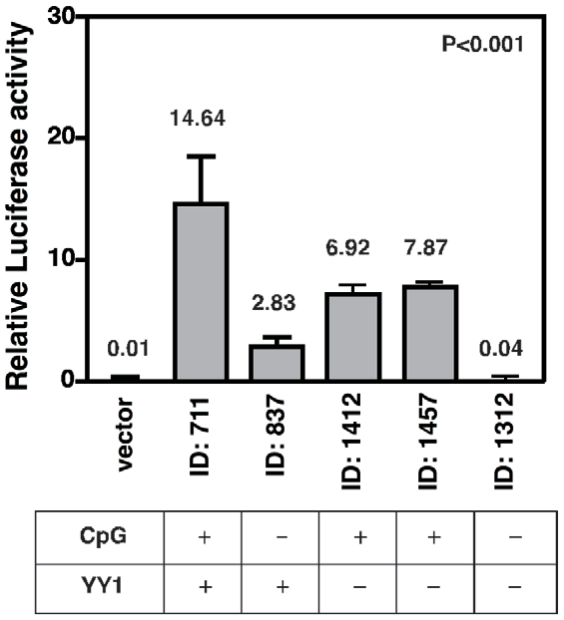
Relative luciferase activity of each T_F_ promoter is shown after normalized for Renilla luciferase reporter. In this assay, the negative control GL3 vector was used. The arbitrary units of each relative luciferase activity are converted into the percentage. Error bars show s.d. of three independent experiments *P<0.001. The bottom panel shows the characteristic features of each TF promoter.

To do this, we isolated the complete sequences of two T_F_ promoters (IDs: 711 and 837) from the X-chromosome and cloned them into the upstream region of the luciferase reporter gene. BLAST analysis of the promoter sequences showed that both promoters were homologous and almost identical in terms of the size, number of T_F_ monomers and the position of YY1 and E2F binding sites, however only one of the promoters contained a CpG island (that is ID: 711). To test whether these promoters were able to initiate transcription, we transfected mouse NIH3T3 cells with luciferase reporter constructs driven by either a CpG or non-CpG L1 promoter and performed a dual-luciferase assay with the Renilla luciferase plasmid (**Figure 10**). Remarkably, the transcript level of the CpG-island-containing L1 promoter was approximately five-fold higher than that of the non-CpG promoter (t test, *p*<0.001). As expected, luciferase expression was barely detectable in vector alone-transfected control cells. This finding suggests that CpG islands in mouse L1 elements are part of a promoter regulatory region that is required for the elevated expression of L1 elements in mouse cells. Consistent with this study, a previous report of rat L1 elements also revealed that L1 activation occurs only when the promoter contains a CpG island at its 5′-UTR region [42].

Because both T_F_ promoters (IDs: 711 and 837) contain intact YY1 binding sites except for the presence or absence of a CpG islands, we next determined whether the presence of the YY1 transcriptional factors binding sites within the CpG islands is required for L1 promoter activity. To do this, we isolated two additional T_F_ promoters (IDs: 1412 and 1457), which contained mutations in the YY1 motifs within a CpG island, and cloned them into luciferase reporter constructs. By measuring the luciferase activities, we found that the promoter activities of these mutated YY1 sites decreased by two-fold (p<0.001) compared with the intact YY1 motifs within the CpG islands (ID: 711). On the other hand, the T_F_ promoter (ID: 1312) that contained both YY1 mutations and an absence of CpG islands did not express any luciferase at all. Together, this data clearly indicates that, like humans, CpG islands in L1 promoters may play an important role in the transcriptional activation of mouse L1 elements and that the presence of CpG islands most likely influences the ability of YY1 motifs to act in transcriptional initiation of promoters. Further studies are required to identify the critical CpG sites and to conform the roles and functional significance of YY1 motifs within a CpG island of mouse L1 promoter. Nonetheless, our study show that only a half of mouse L1 elements (710 out of 1,501) are capable of activity–a significantly lower than we initially estimated. Of the 710 mouse L1 elements, only 124 contain previously unreported CpG islands in their promoters that showed a high level of promoter activity and that there is a difference in the level of expression with and without a CpG island.

## Materials and Methods

### Acquisition of L1 sequences

Sequences and annotation data for mouse L1 elements were retrieved from the L1Base (UCSC mm5, Ensembl version 24.33) database (http://l1base.molgen.mpg.de/). L1 elements devoid of intact 5′-UTRs, ORFs, and 3′-UTR sequences were removed from the dataset to ensure that it contained only full-length, potentially active L1 elements (n = 1467). We mapped the genomic locations of all the L1 elements with the UCSC mouse genome assembly (March 2006, NCBI Build 35, version mm8) using BLAT analysis, with data accuracy greater than 0.98. Classification of L1 subfamilies was carried out using the RepeatMasker program (provided by Ensembl) and a customised version of the monomer search modules of L1Xplorer [16], which uses Matcher from the EMBOSS package and template sequences from published reports.

### Annotation of genes in potentially active L1 elements

We downloaded the RefSeq database of the mouse genome (NCBI Build 36, version mm8) from the UCSC Genome Browser (http://genome.ucsc.edu/) and analyzed the position and location of L1 elements in the RefSeq gene annotations using a Perl script. For each L1 element, we analyzed the genomic regions extending 100-kb upstream of the L1 start position and 100-kb downstream of the L1 end position. Sense and antisense orientation of genes were defined relative to the L1 element being analysed; genes orientated in the same direction as the L1 element are designated “sense” and genes in the opposite direction are designated as “antisense.” The nearest gene adjacent to each L1 element and the distance from the gene to the L1 element was recorded. To confirm the gene annotation, a BLAST query was performed separately against the RefSeq cDNA database. For genes with no available functional annotations, we performed a search against the NCBI non-redundant or FANTOM3 databases to identify any homologous genes with functional annotations. Genes with no functional annotations were classified as “hypothetical genes” if they encoded an unidentified protein with more than 100 amino acids.

### Analysing the promoter regions of L1 elements

The promoter sequences of L1 elements from L1Xplorer or the UCSC genome database were extracted using a suite of Perl scripts that detect and extract the core L1 promoter sequences by performing BLAST searches. The accession numbers of the L1 promoter are provided in **Table S5 and S6**. GC content of the promoters was calculated by dividing the number of GC bases by the total number of bases in each region of analysis. For each of these promoters, the positional specific scoring matrix generated by Perl scripting was used to calculate the frequency of GC dinucleotides. CpG Islands were identified using the EMBOSS CpGPlot analysis (http://www.ebi.ac.uk/Tools/emboss/cpgplot/). For identification of transcriptional factor binding site motifs, each promoter was searched by using TRANSFAC (version 7, 2005) (http://www.gene-regulation.com/pub/databases.html) with a cut-off value >0.9. The distances between the matches and the ATG start site were calculated and the number of matches in every monomer from the ATG site was plotted.

### Promoter Construction and Luciferase assay

A full-length promoter of TF elements was amplified by PCR from mouse genomic DNA and cloned into the reporter plasmid pGL3 (Promega) upstream of the luciferase gene (primers: 711FOR, 5′-TTCCTCGAGCCCAGAATAACAATCATCCA-3′; 837FOR, 5′-TTCCTCGAGGCAGACAACCTTACGTTATG-3′; 1412FOR: 5′-TTCCTCGAGTCCTCTTGGGTGAATTTTCTTC-3′; 1457 FOR: 5′-TTCCTCGAGAGATGGGGACACACACATCC-3′; 1312FOR: 5′-TTCCTCGAGGGGTGTTTAGTAACCATGTCTGG-3′ and REV: 5′-AATAAGCTTCTGGTAATCTCTGGAGTTAGTAGT-3′). All plasmids with correct inserts were confirmed by sequencing from both ends to ensure that the correct sequence was cloned. A Renilla luciferase vector, pRL-CMV (Promega) was used to correct the differences in transfection efficiency. Mouse NIH3T3 fibroblast cells were cotransfected with the modified pGL3 firefly luciferase (under the control of each TF promoter) and the Renilla luciferase reporter plasmid. Firefly and Renilla luciferase assays were measured after 48 h with the Dual-Luciferase Reporter assay system (Promega). Firefly activity was normalized to Renilla activity, as described previously [46]. Data shown are the average of three independent experiments with each experiment performed in triplicate, and analysed using the PRISM (GraphPad, version 5) Software Tool.

### Statistical analysis

Statistical analysis of data and supplementary methods are available in the online version of the paper.

## Supporting Information

Table S1 The statistical analysis of the number of monomers in three L1 subfamilies.
(0.01 MB PDF)

Table S2 The distribution of L1 elements in mouse chromosomes and the statistical analysis of correlations between chromosome size and L1 density.
(0.10 MB PDF)

Table S3 The distribution of L1 elements in intergenic regions in mouse chromosomes.
(0.04 MB PDF)

Table S4 The distribution of L1 elements with neighboring genes in mouse chromosomes.
(0.04 MB PDF)

Table S5 The list of L1s' neighbouring genes and their locations.
(0.09 MB PDF)

Table S6 List of tissue-specific genes in the vicinity of L1 elements within 20-kb distance.
(0.02 MB PDF)

Table S7 The mutation rates at the YY1 transcription binding sites in Tf subfamily.
(0.01 MB PDF)
