# Supplementary material for: The Impact of CpG Island on Defining Transcriptional Activation of the Mouse L1 Retrotransposable Elements
Source: PLoS One. 2010 Jun 29;5(6):e11353. doi: 10.1371/journal.pone.0011353 (PMC2894050; doi:10.1371/journal.pone.0011353)
Supplement: Table S1 — The statistical analysis of the number of monomers in three L1 subfamilies. (0.01 MB PDF) [file pone.0011353.s001.pdf]

Table 1: The statistical analysis of the number of monomers in three subfamilies

	A subfamily	TF subfamily	GF subfamily
Number of values	473	875	116
Minimum	1	1	2
25% Percentile	3	3	4
Median	4	4	5
75% Percentile	5	5	7
Maximum	17	12	15
Mean	4.264	4.114	5.397
Std. Deviation	2.019	1.771	2.303
Std. Error	0.09283	0.05989	0.2138
Lower 95% CI of mean	4.082	3.997	4.973
Upper 95% CI of mean	4.447	4.232	5.82
Sum	2017	3600	626

One sample t test

Theoretical mean	0	0	0
Actual mean	4.264	4.114	5.397
Discrepancy	-4.264	-4.114	-5.397
95% CI of discrepancy	4.082 to 4.446	3.997 to 4.232	4.973 to 5.820
t, df	t=45.94 df=472	t=68.70 df=874	t=25.24 df=115
P value (two tailed)	P<0.0001	P<0.0001	P<0.0001
Significant (alpha=0.05)?	Yes	Yes	Yes
Sum	2017	3600	626

One-way analysis of variance

P value	P<0.0001
P value summary	***
Are means signif. different? (P < 0.05)	Yes
Number of groups	3
F	23.32
R squared	0.03093
Bartlett's test for equal variances	
Bartlett's statistic (corrected)	21.48
P value	P<0.0001
P value summary	***
Do the variances differ signif. (P < 0.05)	Yes

**ANOVA Table**	**SS**	**df**	**MS**
Treatment (between columns)	168.4	2	84.2
Residual (within columns)	5276	1461	3.611
Total	5445	1463	
