# Supplementary material for: The Impact of CpG Island on Defining Transcriptional Activation of the Mouse L1 Retrotransposable Elements
Source: PLoS One. 2010 Jun 29;5(6):e11353. doi: 10.1371/journal.pone.0011353 (PMC2894050; doi:10.1371/journal.pone.0011353)
Supplement: Table S2 — The distribution of L1 elements in mouse chromosomes and the statistical analysis of correlations between chromosome size and L1 density. (0.10 MB PDF) [file pone.0011353.s002.pdf]

Table 2: The distribution of L1 elements in mouse chromosomes and the statistical analysis of correlations between chromosome size and L1 density.

Chromosome	Chromosome Size (bp)	L1 size (bp)	Ratio (%)	L1 number
1	197069962	898963	0.46	86
2	181976762	1792916	0.99	172
3	159872112	812727	0.51	77
4	155029701	1663515	1.07	159
5	152003063	639855	0.42	61
6	149525685	617732	0.41	59
7	145134094	597276	0.41	57
8	132085098	604507	0.46	58
9	124000669	302175	0.24	29
10	129959148	470071	0.36	45
11	121798632	826095	0.68	79
12	120463159	479947	0.40	46
13	120614378	378225	0.31	35
14	123978870	510995	0.41	48
15	103492577	386611	0.37	37
16	98252459	396399	0.40	38
17	95177420	260458	0.27	25
18	90736837	745848	0.82	71
19	61321190	260551	0.42	25
X	165556469	2996914	1.81	286
Y	16029404	83565	0.52	8
Total	2644077689	15725345	0.59	1501

Cross correlation coefficient test

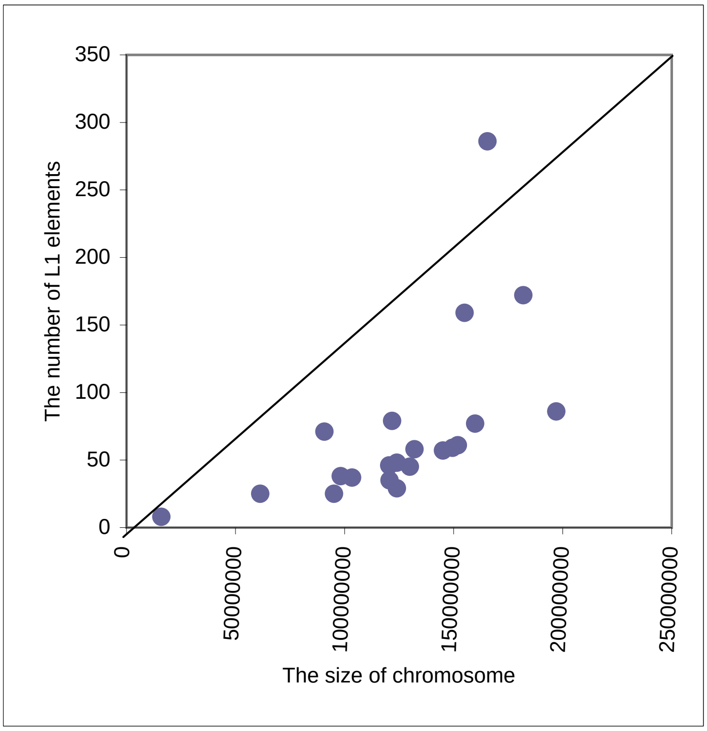

Number of XY Pairs	21
Pearson r (correlation)	0.5885
95% confidence interval	0.2102 to 0.8136
P value (two-tailed)	0.005
P value summary	**
Correlation significant? (alpha=0.05)	Yes
R squared	0.3464
**Chi-square**	
Chi-square, df	637.7, 20
P value	P<0.0001
P value summary	***
One- or two-sided	NA
Statistically significant? (alpha<0.05)	Yes

Supplementary Method

Statistical analysis

The correlation between size of the chromosome and density of L1 elements on each individual chromosome was analyzed by cross correlation coefficient test (Pearson's test).

$$Correl(X,Y) = \frac{\sum(x-\bar{x})(y-\bar{y})}{\sqrt{\sum(x-\bar{x})^2\sum(y-\bar{y})^2}}$$

(X and Y represent the average size of chromosome and L1 elements, respectively).

To test the statistical analysis of correlation between the number of L1 elements and expected frequency of L1s per chromosome, the determination coefficient (R^2) test and Chi-square test were used.

Determination coefficient:

$$R^2 = 1 - \frac{\sum_{i=1}^{n}(real_i - obs_i)^2}{\sum_{i=1}^{n}(real_i - real_i)^2}$$

(The 'real' represents the actual frequency of L1 elements. The 'obs' indicates the expected frequency of L1 elements; n= number of chromosome)

Chi-square test:

$$X^2 = \sum_{i=1}^{r}\sum_{j=1}^{c}\frac{(A_{ij} - E_{ij})^2}{E_{ij}}$$

(Aij representing the actual frequency of L1 elements; Eij is the expected frequency of L1 elements (r) and c is the number of chromosome)
