# Supplementary material for: The Impact of CpG Island on Defining Transcriptional Activation of the Mouse L1 Retrotransposable Elements
Source: PLoS One. 2010 Jun 29;5(6):e11353. doi: 10.1371/journal.pone.0011353 (PMC2894050; doi:10.1371/journal.pone.0011353)
Supplement: Table S3 — The distribution of L1 elements in intergenic regions in mouse chromosomes. (0.04 MB PDF) [file pone.0011353.s003.pdf]

Table 3: The distribution of L1 elements in intergenic regions in mouse chromosomes

Chromosome	Size (bp)	The size of intergenic L1s (bp)	Ratio	L1 number	Expected frequency *
1	197069962	627440	0.32%	60	71.18
2	181976762	1135569	0.62%	109	65.73
3	159872112	562346	0.35%	53	57.74
4	155029701	1002718	0.65%	96	55.99
5	152003063	357762	0.24%	34	54.90
6	149525685	440190	0.29%	42	54.01
7	145134094	346393	0.24%	33	52.42
8	132085098	458440	0.35%	44	47.71
9	124000669	166674	0.13%	16	44.79
10	129959148	332878	0.26%	32	46.94
11	121798632	502511	0.41%	48	43.99
12	120463159	333804	0.28%	32	43.51
13	120614378	252983	0.21%	23	43.56
14	123978870	416948	0.34%	39	44.78
15	103492577	240477	0.23%	23	37.38
16	98252459	281559	0.29%	27	35.49
17	95177420	135203	0.14%	13	34.38
18	90736837	464309	0.51%	44	32.77
19	61321190	125080	0.20%	12	22.15
X	165556469	1781034	1.08%	170	59.80
Y	16029404	52242	0.33%	5	5.79
Total	2644077689	10016560	0.38%	955	955

* Expected frequency of L1 per chromosome
(= Each chromosome size/total chromosome size x total L1 munbers)

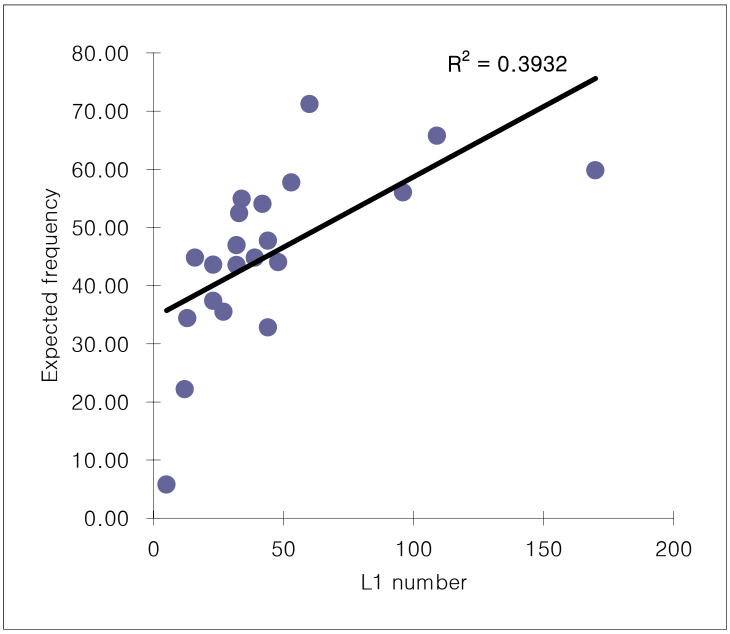

Chi-square, df	3.339, 1
P value	0.0677
P value summary	ns
One- or two-sided	NA
Statistically significant? (alpha<0.05)	No
Data analyzed	
Number of rows	21
Number of columns	2
