# Supplementary material for: The Impact of CpG Island on Defining Transcriptional Activation of the Mouse L1 Retrotransposable Elements
Source: PLoS One. 2010 Jun 29;5(6):e11353. doi: 10.1371/journal.pone.0011353 (PMC2894050; doi:10.1371/journal.pone.0011353)
Supplement: Table S4 — The distribution of L1 elements with neighboring genes in mouse chromosomes. (0.04 MB PDF) [file pone.0011353.s004.pdf]

Table 4: The distribution of L1 elements with neighbouring genes in mouse chromosomes

Chromosome	Size (bp)	The size of intergenic L1s (bp)	Ratio	L1 number	Expected frequency *
1	197069962	271027	0.14%	26	40.69
2	181976762	656287	0.36%	63	37.58
3	159872112	249848	0.16%	24	33.01
4	155029701	660067	0.43%	63	32.01
5	152003063	281741	0.19%	27	31.39
6	149525685	177326	0.12%	17	30.88
7	145134094	250477	0.17%	24	29.97
8	132085098	145539	0.11%	14	27.28
9	124000669	135312	0.11%	13	25.61
10	129959148	137114	0.11%	13	26.84
11	121798632	325375	0.27%	31	25.15
12	120463159	146170	0.12%	14	24.88
13	120614378	125145	0.10%	12	24.91
14	123978870	93968	0.08%	9	25.60
15	103492577	146025	0.14%	14	21.37
16	98252459	114769	0.12%	11	20.29
17	95177420	125164	0.13%	12	19.65
18	90736837	284036	0.31%	27	18.74
19	61321190	135390	0.22%	13	12.66
X	165556469	1218526	0.74%	116	34.19
Y	16029404	31323	0.20%	3	3.31
Total	2644077689	5710629	0.22%	546	546

* Expected frequency of L1 per chromosome
(= Each chromosome size/total chromosome size x total L1 munbers)

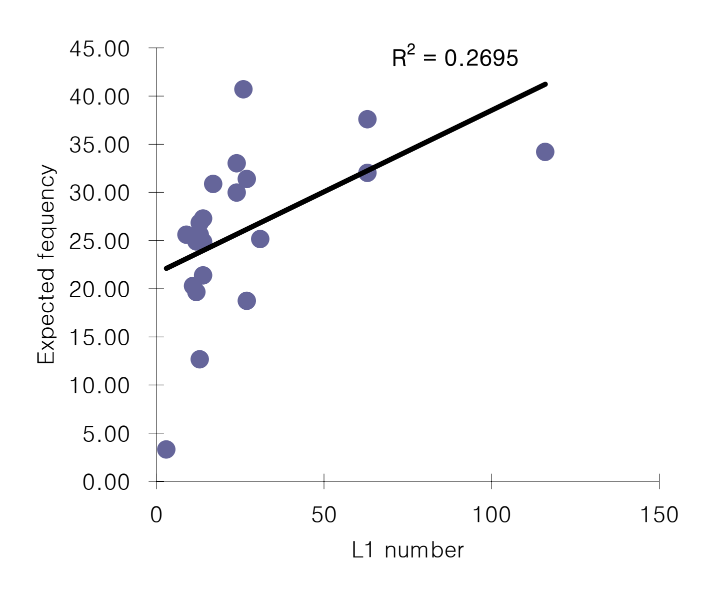

Chi-square, df	6.688, 1
P value	0.0097
P value summary	**
One- or two-sided	NA
Statistically significant? (alpha<0.05)	Yes
Data analyzed	
Number of rows	21
Number of columns	2
