# Supplementary material for: The Impact of CpG Island on Defining Transcriptional Activation of the Mouse L1 Retrotransposable Elements
Source: PLoS One. 2010 Jun 29;5(6):e11353. doi: 10.1371/journal.pone.0011353 (PMC2894050; doi:10.1371/journal.pone.0011353)
Supplement: Table S5 — The list of L1s' neighbouring genes and their locations. (0.09 MB PDF) [file pone.0011353.s005.pdf]

Table 5: The list of L1s' neighbouring genes and their locations

No	LINE ID	chr number	refseq ID	LINE start	LINE end	refseq start	refseq end	LINE strand	Distance	refseq strand	Orientation	Refseq Difinition
1	UID2	chr18	NM_026092	4219421	4229862	4165829	4182234	+	-100k	+	S	lysozyme-like 1 precursor
2	UID4	chr18	NM_001100449	14872664	14883105	14926262	15043377	+	+100k	+	AS	TAF4B RNA polymerase II, TATA box binding
3	UID4	chr18	NM_001163609	14872664	14883105	14849168	14905317	+	-50k	+	S	proteasome alpha 8 subunit
4	UID10	chr18	NM_001033532	25353995	25364027	25312029	25610331	+	-50k	+	S	hypothetical protein LOC225289
5	UID10	chr18	NM_001142697	25353995	25364027	25279055	25308975	+	-100k	-	AS	tubulin polyglutamylase complex subunit 2
6	UID10	chr18	NM_001142698	25353995	25364027	25270232	25311887	+	-100k	-	AS	tubulin polyglutamylase complex subunit 2
7	UID10	chr18	NM_001004361	25353995	25364027	25279055	25311887	+	-100k	-	AS	tubulin polyglutamylase complex subunit 2
8	UID11	chr18	NM_001109988	33516959	33527400	33561829	33588840	+	+50k	-	AS	neuronal protein 3.1
9	UID11	chr18	NM_053078	33516959	33527400	33561829	33588445	+	+50k	-	AS	neuronal protein 3.1
10	UID11	chr18	NM_001109990	33516959	33527400	33561829	33588232	+	+50k	-	AS	neuronal protein 3.1
11	UID11	chr18	NM_001109989	33516959	33527400	33561829	33588558	+	+50k	-	AS	neuronal protein 3.1
12	UID13	chr18	NM_011901	37753607	37764050	37767995	37770121	+	+20k	-	AS	
13	UID13	chr18	NM_033587	37753607	37764050	37811373	37967844	+	+100k	+	AS	protocadherin gamma subfamily A, 4
14	UID13	chr18	NM_033589	37753607	37764050	37833202	37967844	+	+100k	+	AS	protocadherin gamma subfamily A, 6
15	UID13	chr18	NM_033575	37753607	37764050	37815832	37967846	+	+100k	+	AS	protocadherin gamma subfamily B, 2
16	UID13	chr18	NM_033586	37753607	37764050	37800308	37967846	+	+50k	+	AS	protocadherin gamma subfamily A, 3
17	UID13	chr18	NM_033588	37753607	37764050	37820474	37967844	+	+100k	+	AS	protocadherin gamma subfamily A, 5
18	UID13	chr18	NM_175770	37753607	37764050	37766464	37770178	+	+20k	-	AS	TAF7 RNA polymerase II, TATA box binding protein
19	UID13	chr18	NM_033590	37753607	37764050	37840807	37967846	+	+100k	+	AS	protocadherin gamma subfamily A, 7
20	UID13	chr18	NM_033585	37753607	37764050	37795078	37967844	+	+50k	+	AS	protocadherin gamma subfamily A, 2
21	UID13	chr18	NM_033576	37753607	37764050	37846527	37967844	+	+100k	+	AS	protocadherin gamma subfamily B, 4
22	UID13	chr18	NM_033584	37753607	37764050	37787918	37967844	+	+50k	+	AS	protocadherin gamma subfamily A, 1
23	UID13	chr18	NM_033574	37753607	37764050	37806431	37967844	+	+100k	+	AS	protocadherin gamma subfamily B, 1
24	UID13	chr18	NM_033591	37753607	37764050	37851679	37967846	+	+100k	+	AS	protocadherin gamma subfamily A, 8
25	UID13	chr18	NM_001159275	37753607	37764050	37763351	37764697	+	+10k	-	AS	mutant ornithine transporter 2
26	UID15	chr18	NM_001004061	50375808	50386206	50403698	50408349	+	+50k	+	AS	family with sequence similarity 170, member A
27	UID19	chr18	NM_001001979	57336493	57346879	57258843	57422835	+	-100k	+	S	MEGF10 protein precursor
28	UID21	chr18	NM_016977	67062625	67075740	66983076	66985841	+	-100k	-	AS	melanocortin 4 receptor
29	UID22	chr18	NM_029019	70632240	70642681	70597913	70626432	+	-50k	+	S	START domain containing protein 6
30	UID22	chr18	NM_172967	70632240	70642681	70578508	70597849	+	-100k	-	AS	hypothetical protein LOC269033 precursor
31	UID22	chr18	NM_010773	70632240	70642681	70693660	70751500	+	+100k	+	AS	methyl-CpG binding domain protein 2
32	UID22	chr18	NM_001136090	70632240	70642681	70634049	70655507	+	+5k	-	AS	DNA polymerase iota isoform 1
33	UID22	chr18	NM_011972	70632240	70642681	70634049	70655690	+	+5k	-	AS	DNA polymerase iota isoform 2
34	UID27	chr18	NM_001109743	76987662	76998098	77060854	77104792	+	+100k	+	AS	functional Smad suppressor element on chromosome
35	UID28	chr18	NM_053099	78856552	78866993	78912458	79271470	+	+100k	-	AS	SET binding protein 1
36	UID30	chr18	NM_026295	80664777	80675218	80570017	80631726	+	-100k	-	AS	CTD (carboxy-terminal domain, RNA polymerase II
37	UID35	chr18	NM_178687	89268102	89278542	89331619	89404520	+	+100k	+	AS	platelet and T cell activation antigen 1 isoform

**38**	UID35	chr18	NM_001039149	89268102	89278542	89331619	89404520	+	+100k	+	AS	platelet and T cell activation antigen 1 isoform
**39**	UID37	chr18	NM_018821	89073018	89083416	89002072	89028400	-	-100k	-	AS	suppressor of cytokine signaling 6
**40**	UID37	chr18	NM_175542	89073018	89083416	89105982	89265207	-	+50k	+	AS	rotatin
**41**	UID45	chr18	NM_023149	84737021	84747419	84801661	84819826	-	+100k	-	S	CNDP dipeptidase 2
**42**	UID45	chr18	NM_177450	84737021	84747419	84744701	84784288	-	+10k	-	S	carnosine dipeptidase 1
**43**	UID46	chr18	NM_178280	81041243	81051726	81128432	81148637	-	+100k	-	S	sal-like protein 3
**44**	UID47	chr18	NM_145356	76008492	76018936	75945542	76273929	-	-100k	+	AS	zinc finger and BTB domain containing 7C
**45**	UID50	chr18	NM_008560	68599604	68610050	68532275	68554628	-	-100k	-	AS	melanocortin 2 receptor
**46**	UID52	chr18	NM_001001803	62744025	62754423	62717782	62721633	-	-50k	-	AS	serine peptidase inhibitor, Kazal type 7
**47**	UID52	chr18	NM_177829	62744025	62754423	62674279	62786757	-	-100k	+	AS	serine protease inhibitor Kazal type 10
**48**	UID53	chr18	NM_008313	62410752	62421193	62449572	62593171	-	+50k	+	AS	serotonin 5-HT4 receptor
**49**	UID54	chr18	NM_001033281	53684849	53695289	53589914	53701226	-	-100k	+	AS	PR domain containing 6
**50**	UID55	chr18	NM_026108	50656147	50666545	50692840	50693884	-	+50k	-	S	haloacid dehalogenase-like hydrolase domain
**51**	UID56	chr18	NM_001004061	50386305	50396746	50403698	50408349	-	+20k	+	AS	family with sequence similarity 170, member A
**52**	UID61	chr18	NM_198117	37156790	37167231	37065178	37313632	-	-100k	+	AS	protocadherin alpha 2
**53**	UID61	chr18	NM_138662	37156790	37167231	37072180	37313631	-	-100k	+	AS	protocadherin alpha 3
**54**	UID61	chr18	NM_007766	37156790	37167231	37078662	37313631	-	-100k	+	AS	protocadherin alpha 4 precursor
**55**	UID61	chr18	NM_009959	37156790	37167231	37086413	37313631	-	-100k	+	AS	protocadherin alpha 5
**56**	UID61	chr18	NM_007767	37156790	37167231	37093729	37313631	-	-100k	+	AS	protocadherin alpha 6
**57**	UID61	chr18	NM_009957	37156790	37167231	37099897	37313631	-	-100k	+	AS	protocadherin alpha 7
**58**	UID61	chr18	NM_201243	37156790	37167231	37118440	37313631	-	-50k	+	AS	protocadherin alpha 8
**59**	UID61	chr18	NM_138661	37156790	37167231	37123853	37313631	-	-50k	+	AS	protocadherin alpha 9
**60**	UID61	chr18	NM_009961	37156790	37167231	37131293	37313631	-	-50k	+	AS	protocadherin alpha 10
**61**	UID61	chr18	NM_138663	37156790	37167231	37146203	37313631	-	-20k	+	AS	protocadherin alpha 12
**62**	UID61	chr18	NM_009960	37156790	37167231	37136831	37313631	-	-20k	+	AS	protocadherin alpha 11
**63**	UID61	chr18	NM_001003671	37156790	37167231	37216109	37313631	-	+100k	+	AS	protocadherin alpha subfamily C, 1
**64**	UID62	chr18	NM_001042768	32185574	32196060	32266135	32281215	-	+100k	-	S	protein C
**65**	UID62	chr18	NM_001042767	32185574	32196060	32266135	32282580	-	+100k	-	S	protein C
**66**	UID62	chr18	NM_008934	32185574	32196060	32266135	32281215	-	+100k	-	S	protein C
**67**	UID62	chr18	NM_032394	32185574	32196060	32102243	32179969	-	-100k	-	AS	myosin VIIB
**68**	UID62	chr18	NM_173441	32185574	32196060	32210743	32247341	-	+50k	+	AS	IWS1 homolog
**69**	UID62	chr18	NM_001025381	32185574	32196060	32086023	32092646	-	-100k	-	AS	G protein-coupled receptor 17
**70**	UID63	chr18	NM_026529	24711884	24722327	24613880	24620777	-	-100k	+	AS	hypothetical protein LOC68046
**71**	UID63	chr18	NM_021448	24711884	24722327	24746970	24781840	-	+50k	+	AS	signal transducer and activator of transcription
**72**	UID63	chr18	NM_144861	24711884	24722327	24627971	24673214	-	-100k	-	AS	cDNA sequence BC021395
**73**	UID63	chr18	NM_139143	24711884	24722327	24722890	24746827	-	+20k	-	S	solute carrier family 39 (metal ion
**74**	UID63	chr18	NM_026779	24711884	24722327	24796700	24844566	-	+100k	+	AS	molybdenum cofactor sulfurase
**75**	UID64	chr18	NM_001162942	23930358	23940841	23895342	24036871	-	-50k	+	AS	microtubule-associated protein, RP/EB family
**76**	UID64	chr18	NM_001162941	23930358	23940841	23896734	24036871	-	-50k	+	AS	microtubule-associated protein, RP/EB family
**77**	UID64	chr18	NM_153058	23930358	23940841	23946979	24036871	-	+20k	+	AS	microtubule-associated protein, RP/EB family
**78**	UID75	chr3	NM_008862	7291215	7301656	7349676	7428438	+	+100k	+	AS	protein kinase inhibitor, alpha
**79**	UID89	chr3	NM_153384	58937494	58947892	58933143	58973141	+	-5k	-	AS	Usher syndrome 3A homolog isoform 1
**80**	UID89	chr3	NM_153385	58937494	58947892	58933143	58973141	+	-5k	-	AS	Usher syndrome 3A homolog isoform 2

**81**	UID89	chr3	NM_153386	58937494	58947892	58933143	58973141	+	-5k	-	AS	Usher syndrome 3A homolog isoform 3
**82**	UID92	chr3	NM_028712	61529543	61539984	61452435	61456632	+	-100k	+	S	RAP2B, member of RAS oncogene family precursor
**83**	UID94	chr3	NM_001037923	65840901	65851134	65754163	65774108	+	-100k	+	S	hypothetical protein LOC624866
**84**	UID95	chr3	NM_145820	66156511	66166909	66142756	66384748	+	-20k	-	AS	VEPH isoform A
**85**	UID95	chr3	NM_028357	66156511	66166909	66142756	66224367	+	-20k	-	AS	VEPH isoform B
**86**	UID101	chr3	NM_001039195	80825590	80836031	80777418	80888718	+	-50k	-	AS	glutamate receptor, ionotropic, AMPA 2 isoform
**87**	UID101	chr3	NM_001083806	80825590	80836031	80770862	80888718	+	-100k	-	AS	glutamate receptor, ionotropic, AMPA 2 isoform
**88**	UID101	chr3	NM_013540	80825590	80836031	80770862	80888718	+	-100k	-	AS	glutamate receptor, ionotropic, AMPA 2 isoform
**89**	UID102	chr3	NM_025416	94351032	94361473	94427502	94432754	+	+100k	+	AS	thioesterase superfamily member 5
**90**	UID102	chr3	NM_029431	94351032	94361473	94395535	94417936	+	+50k	+	AS	thioesterase superfamily member 4
**91**	UID102	chr3	NM_001136117	94351032	94361473	94447847	94449970	+	+100k	+	AS	hypothetical protein LOC271944
**92**	UID102	chr3	NM_001146107	94351032	94361473	94259815	94263597	+	-100k	-	AS	TD and POZ domain containing-like
**93**	UID103	chr3	NM_011656	94632343	94642784	94698162	94744210	+	+100k	-	AS	tuftelin 1
**94**	UID103	chr3	NM_001082484	94632343	94642784	94586083	94668106	+	-50k	-	AS	sorting nexin family member 27 isoform 1
**95**	UID103	chr3	NM_172434	94632343	94642784	94564234	94577597	+	-100k	+	S	CUG-BP and ETR-3 like factor 3
**96**	UID103	chr3	NM_029721	94632343	94642784	94582947	94668106	+	-50k	-	AS	sorting nexin family member 27 isoform 2
**97**	UID103	chr3	NM_025506	94632343	94642784	94550388	94558995	+	-100k	-	AS	RIIa domain-containing protein
**98**	UID104	chr3	NM_011516	102958973	102969411	102947559	103065161	+	-20k	-	AS	synaptonemal complex protein 1
**99**	UID104	chr3	NM_009432	102958973	102969411	102906458	102911775	+	-100k	-	AS	thyroid stimulating hormone, beta subunit
**100**	UID104	chr3	NM_027533	102958973	102969411	102863830	102901353	+	-100k	+	S	tetraspan 2
**101**	UID105	chr3	NM_027193	115905527	115915968	115880186	115921327	+	-50k	+	S	diphthine synthase
**102**	UID105	chr3	NM_021388	115905527	115915968	115999452	116021020	+	+100k	+	AS	exostoses-like 2 isoform a
**103**	UID105	chr3	NM_001163514	115905527	115915968	116000300	116021020	+	+100k	+	AS	exostoses-like 2 isoform a
**104**	UID105	chr3	NM_023214	115905527	115915968	115937948	115999383	+	+50k	-	AS	zinc transporter like 2
**105**	UID105	chr3	NM_001163515	115905527	115915968	115999452	116021020	+	+100k	+	AS	exostoses-like 2 isoform b
**106**	UID105	chr3	NR_015487	115905527	115915968	115873582	115880134	+	-50k	-	AS	
**107**	UID105	chr3	NR_027894	115905527	115915968	115873582	115880063	+	-50k	-	AS	
**108**	UID105	chr3	NR_027895	115905527	115915968	115879151	115880124	+	-50k	-	AS	
**109**	UID110	chr3	NM_021382	134816385	134826826	134766393	134871959	+	-50k	+	S	tachykinin receptor 3
**110**	UID112	chr3	NM_030601	144763777	144774218	144734469	144756863	+	-50k	-	AS	chloride channel calcium activated 2
**111**	UID112	chr3	NM_001039222	144763777	144774218	144816710	144833397	+	+100k	-	AS	hypothetical protein LOC622139
**112**	UID112	chr3	NM_139148	144763777	144774218	144760009	144786688	+	-5k	-	AS	chloride channel calcium activated 4
**113**	UID112	chr3	NM_009899	144763777	144774218	144667701	144698125	+	-100k	-	AS	chloride channel calcium activated 1
**114**	UID112	chr3	NM_001033199	144763777	144774218	144848306	144869915	+	+100k	-	AS	calcium activated chloride channel
**115**	UID117	chr3	NM_001081263	153959825	153970259	153910821	154207013	-	-50k	+	AS	solute carrier family 44, member 5
**116**	UID118	chr3	NM_133871	151608945	151619386	151668308	151687345	-	+100k	-	S	interferon-induced protein 44
**117**	UID118	chr3	NM_031367	151608945	151619386	151696122	151700277	-	+100k	-	S	histocompatibility 28
**118**	UID124	chr3	NM_146139	109739860	109750258	109702062	109813750	-	-50k	+	AS	vav 3 oncogene isoform 2
**119**	UID127	chr3	NM_011478	92631281	92641679	92542221	92542938	-	-100k	-	AS	small proline-rich protein 3
**120**	UID127	chr3	NM_009264	92631281	92641679	92569356	92571284	-	-100k	-	AS	small proline-rich protein 1A
**121**	UID127	chr3	NM_173070	92631281	92641679	92585665	92585896	-	-50k	-	AS	small proline-rich protein 4
**122**	UID127	chr3	NM_008574	92631281	92641679	92669268	92674427	-	+50k	-	S	sperm mitochondria-associated cysteine-rich
**123**	UID127	chr3	NM_008412	92631281	92641679	92656304	92659118	-	+50k	-	S	involucrin

**124**	UID129	chr3	NM_133187	80044402	80054843	79971856	80032204	-	-100k	+	AS	expressed in nerve and epithelium during
**125**	UID134	chr3	NM_001162884	59481278	59491719	59404663	59432185	-	-100k	-	AS	immunoglobulin superfamily, member 10 precursor
**126**	UID135	chr3	NM_028808	59366813	59377211	59295824	59298811	-	-100k	-	AS	purinergic receptor P2Y13
**127**	UID135	chr3	NM_032399	59366813	59377211	59266851	59268014	-	-100k	-	AS	G protein-coupled receptor 87
**128**	UID135	chr3	NM_027571	59366813	59377211	59304199	59350760	-	-100k	-	AS	purinergic receptor P2Y12
**129**	UID135	chr3	NM_001162884	59366813	59377211	59404663	59432185	-	+50k	-	S	immunoglobulin superfamily, member 10 precursor
**130**	UID137	chr3	NM_026225	52997788	53008186	53070051	53105152	-	+100k	-	S	component of oligomeric golgi complex 6
**131**	UID142	chr3	NM_172679	37037752	37047974	37054654	37244582	-	+20k	+	AS	fragile site-associated protein
**132**	UID145	chr3	NM_007963	30056212	30066653	30142376	30204277	-	+100k	-	S	MDS1 and EVI1 complex locus isoform 1
**133**	UID147	chr3	NM_138666	25662948	25673346	25622913	26522982	-	-50k	-	AS	neuroligin 1 isoform 1
**134**	UID147	chr3	NM_001163387	25662948	25673346	25622913	26344380	-	-50k	-	AS	neuroligin 1 isoform 2
**135**	UID149	chr3	NM_001081687	16113715	16124158	16140220	16198483	-	+50k	-	S	hypothetical protein LOC381484
**136**	UID152	chr11	NM_015785	11185074	11195515	11182515	11362411	+	-5k	-	AS	zona pellucida binding protein
**137**	UID158	chr11	NM_011023	21804003	21814444	21894766	21901654	+	+100k	-	AS	orthodenticle 1
**138**	UID160	chr11	NM_025923	26323933	26334374	26287083	26371876	+	-50k	+	S	Fanconi anemia, complementation group L
**139**	UID160	chr11	NM_027260	26323933	26334374	26371401	26493920	+	+50k	-	AS	vaccinia related kinase 2
**140**	UID161	chr11	NR_002890	26629847	26642710	26685107	26687859	+	+100k	+	AS	
**141**	UID162	chr11	NM_133767	29335712	29346156	29426456	29445255	+	+100k	+	AS	mitochondrial translational initiation factor 2
**142**	UID162	chr11	NM_176841	29335712	29346156	29273774	29410811	+	-100k	+	S	coiled coil domain containing 88A
**143**	UID162	chr11	NM_001163454	29335712	29346156	29412876	29415033	+	+100k	-	AS	PrdX-deacylase domain 1 isoform 1
**144**	UID162	chr11	NM_026465	29335712	29346156	29413054	29415033	+	+100k	-	AS	PrdX-deacylase domain 1 isoform 2
**145**	UID167	chr11	NM_013552	40474329	40484770	40544810	40576860	+	+100k	-	AS	hyaluronan mediated motility receptor (RHAMM)
**146**	UID167	chr11	NM_134017	40474329	40484770	40522736	40536036	+	+50k	-	AS	methionine adenosyltransferase II, beta
**147**	UID168	chr11	NM_008073	41729980	41740466	41755279	41844101	+	+50k	-	AS	gamma-aminobutyric acid (GABA) A receptor,
**148**	UID168	chr11	NM_177408	41729980	41740466	41753617	41843742	+	+50k	-	AS	gamma-aminobutyric acid (GABA) A receptor,
**149**	UID172	chr11	NM_001045520	45611028	45621469	45695386	45754048	+	+100k	+	AS	clathrin interactor 1
**150**	UID175	chr11	NM_146471	49183485	49193919	49123572	49124508	+	-100k	+	S	olfactory receptor 1393
**151**	UID175	chr11	NM_146470	49183485	49193919	49136745	49137681	+	-50k	+	S	olfactory receptor 1392
**152**	UID175	chr11	NM_206822	49183485	49193919	49160970	49161906	+	-50k	+	S	olfactory receptor 10
**153**	UID175	chr11	NM_146468	49183485	49193919	49170835	49171771	+	-20k	+	S	olfactory receptor 1391
**154**	UID175	chr11	NM_147065	49183485	49193919	49183956	49184892	+	+5k	+	AS	olfactory receptor 1390
**155**	UID175	chr11	NM_147066	49183485	49193919	49273900	49274836	+	+100k	+	AS	olfactory receptor 1389
**156**	UID175	chr11	NM_010794	49183485	49193919	49087613	49106447	+	-100k	+	S	mannoside acetylglucosaminyltransferase 1
**157**	UID175	chr11	NM_001110148	49183485	49193919	49087613	49106447	+	-100k	+	S	mannoside acetylglucosaminyltransferase 1
**158**	UID175	chr11	NM_001110150	49183485	49193919	49093974	49106447	+	-100k	+	S	mannoside acetylglucosaminyltransferase 1
**159**	UID175	chr11	NM_001110149	49183485	49193919	49090899	49106447	+	-100k	+	S	mannoside acetylglucosaminyltransferase 1
**160**	UID183	chr11	NM_001011863	74120033	74130474	74085585	74086557	+	-50k	+	S	olfactory receptor 406
**161**	UID183	chr11	NM_146707	74120033	74130474	74150476	74151424	+	+50k	-	AS	olfactory receptor 410
**162**	UID183	chr11	NM_146709	74120033	74130474	74162844	74163777	+	+50k	-	AS	olfactory receptor 411
**163**	UID183	chr11	NM_001011851	74120033	74130474	74180865	74181804	+	+100k	+	AS	olfactory receptor 412
**164**	UID183	chr11	NM_146711	74120033	74130474	74022392	74023484	+	-100k	-	AS	olfactory receptor 43
**165**	UID183	chr11	NM_011002	74120033	74130474	74100003	74105841	+	-50k	+	S	olfactory receptor 59
**166**	UID183	chr11	NM_001015046	74120033	74130474	74199677	74406353	+	+100k	-	AS	RAP1 GTPase activating protein 2

**167**	UID185	chr11	NM_026433	89773707	89784148	89846437	89852595	+	+100k	+	AS	transmembrane protein 100
**168**	UID185	chr11	NM_008796	89773707	89784148	89799506	89818984	+	+50k	-	AS	phosphatidylcholine transfer protein
**169**	UID190	chr11	NM_010858	104464304	104474702	104366752	104403309	-	-100k	+	AS	myosin, light polypeptide 4
**170**	UID190	chr11	NM_016780	104464304	104474702	104424089	104486561	-	-50k	+	AS	integrin beta 3 precursor
**171**	UID191	chr11	NM_001100614	99697964	99708363	99631065	99631756	-	-100k	-	AS	hypothetical protein LOC670496
**172**	UID191	chr11	NM_027568	99697964	99708363	99724009	99724980	-	+50k	+	AS	keratin associated protein 31-1
**173**	UID191	chr11	NM_015741	99697964	99708363	99689529	99690090	-	-10k	+	AS	keratin associated protein 9-1
**174**	UID191	chr11	NM_001101613	99697964	99708363	99695276	99696319	-	-5k	+	AS	hypothetical protein LOC670533
**175**	UID191	chr11	NM_027087	99697964	99708363	99614162	99620536	-	-100k	-	AS	keratin associated protein 4-13
**176**	UID191	chr11	NM_001025244	99697964	99708363	99752380	99753315	-	+100k	+	AS	keratin associated protein 31-2
**177**	UID191	chr11	NM_001099313	99697964	99708363	99614275	99625982	-	-100k	-	AS	keratin associated protein 4 family member
**178**	UID191	chr11	NM_001099313	99697964	99708363	99619759	99620536	-	-100k	-	AS	keratin associated protein 4 family member
**179**	UID191	chr11	NM_027087	99697964	99708363	99625167	99625982	-	-100k	-	AS	keratin associated protein 4-13
**180**	UID191	chr11	NM_001085548	99697964	99708363	99601289	99602347	-	-100k	+	AS	keratin associated protein 4 family member
**181**	UID191	chr11	NM_001099312	99697964	99708363	99619637	99620536	-	-100k	-	AS	keratin associated protein 4 family member
**182**	UID191	chr11	NM_001099311	99697964	99708363	99608764	99609382	-	-100k	-	AS	keratin associated protein 4 family member
**183**	UID191	chr11	NM_001085527	99697964	99708363	99764564	99765641	-	+100k	+	AS	keratin associated protein 9-5
**184**	UID191	chr11	NM_001013823	99697964	99708363	99666744	99667695	-	-50k	-	AS	keratin associated protein 4-16
**185**	UID191	chr11	NM_001126323	99697964	99708363	99730840	99731761	-	+50k	+	AS	hypothetical protein LOC670550
**186**	UID192	chr11	NM_028547	91451985	91462382	91391375	91393645	-	-100k	-	AS	kinesin family member 2B
**187**	UID193	chr11	NM_199008	90394219	90404660	90454273	90462067	-	+100k	+	AS	COX11 homolog, cytochrome c oxidase assembly
**188**	UID193	chr11	NM_028011	90394219	90404660	90461780	90503691	-	+100k	-	S	target of myb1-like 1
**189**	UID194	chr11	NM_008796	89719144	89729585	89799506	89818984	-	+100k	-	S	phosphatidylcholine transfer protein
**190**	UID195	chr11	NM_146443	73425952	73436350	73332453	73333392	-	-100k	-	AS	olfactory receptor 382
**191**	UID195	chr11	NM_147023	73425952	73436350	73404992	73405931	-	-50k	-	AS	olfactory receptor 385
**192**	UID195	chr11	NM_207224	73425952	73436350	73418776	73419715	-	-10k	+	AS	olfactory receptor 386
**193**	UID195	chr11	NM_001126489	73425952	73436350	73504972	73530002	-	+100k	+	AS	zinc finger protein 735
**194**	UID197	chr11	NM_025496	62783523	62793921	62767387	62809290	-	-20k	+	AS	CMT1A duplicated region transcript 4
**195**	UID197	chr11	NM_026210	62783523	62793921	62695684	62711379	-	-100k	+	AS	hypothetical protein LOC67510
**196**	UID197	chr11	NM_027660	62783523	62793921	62877853	62911155	-	+100k	+	AS	tektin 3
**197**	UID198	chr11	NM_001083884	58284135	58294515	58195226	58206736	-	-100k	+	AS	RIKEN cDNA 2210415F13 precursor
**198**	UID198	chr11	NM_027339	58284135	58294515	58195226	58206736	-	-100k	+	AS	RIKEN cDNA 2210415F13 precursor
**199**	UID198	chr11	NM_027239	58284135	58294515	58237305	58242218	-	-50k	+	AS	hypothetical protein LOC69864
**200**	UID198	chr11	NM_207527	58284135	58294515	58262343	58269118	-	-50k	-	AS	hypothetical protein LOC403200
**201**	UID198	chr11	NM_146878	58284135	58294515	58271194	58272142	-	-20k	-	AS	olfactory receptor 30
**202**	UID198	chr11	NM_001011770	58284135	58294515	58305943	58306948	-	+50k	-	S	olfactory receptor 332
**203**	UID198	chr11	NM_001011861	58284135	58294515	58317792	58318749	-	+50k	-	S	olfactory receptor 331
**204**	UID198	chr11	NM_146879	58284135	58294515	58345231	58346179	-	+100k	-	S	olfactory receptor 330
**205**	UID198	chr11	NM_001011531	58284135	58294515	58358712	58359651	-	+100k	-	S	olfactory receptor 329
**206**	UID198	chr11	NM_146502	58284135	58294515	58367499	58368432	-	+100k	-	S	olfactory receptor 328
**207**	UID198	chr11	NM_001045542	58284135	58294515	58248753	58257817	-	-50k	+	AS	hypothetical protein LOC624860
**208**	UID203	chr11	NM_173753	54223832	54234273	54281611	54361659	-	+100k	+	AS	folliculin interacting protein 1
**209**	UID203	chr11	NM_029105	54223832	54234273	54214074	54270178	-	-10k	+	AS	hypothetical protein LOC74847

**210**	UID203	chr11	NM_144823	54223832	54234273	54147626	54204962	-	-100k	+	AS	acyl-CoA synthetase long-chain family member 6
**211**	UID203	chr11	NM_001033597	54223832	54234273	54147626	54204962	-	-100k	+	AS	acyl-CoA synthetase long-chain family member 6
**212**	UID203	chr11	NM_001033598	54223832	54234273	54147246	54204962	-	-100k	+	AS	acyl-CoA synthetase long-chain family member 6
**213**	UID203	chr11	NM_001033599	54223832	54234273	54158325	54204962	-	-100k	+	AS	acyl-CoA synthetase long-chain family member 6
**214**	UID204	chr11	NM_013744	50860637	50871045	50765208	50775058	-	-100k	-	AS	zinc finger protein 354B
**215**	UID204	chr11	NM_146910	50860637	50871045	50812442	50813390	-	-50k	+	AS	olfactory receptor 1378
**216**	UID204	chr11	NM_146911	50860637	50871045	50828125	50829049	-	-50k	+	AS	olfactory receptor 1377
**217**	UID204	chr11	NM_146909	50860637	50871045	50850396	50851320	-	-20k	+	AS	olfactory receptor 51
**218**	UID204	chr11	NM_010997	50860637	50871045	50870426	50871368	-	+10k	+	AS	olfactory receptor 54
**219**	UID204	chr11	NM_009329	50860637	50871045	50902679	50916222	-	+50k	+	AS	zinc finger protein 354A
**220**	UID204	chr11	NM_008936	50860637	50871045	50794228	50797055	-	-100k	-	AS	paired like homeodomain factor 1
**221**	UID205	chr11	NM_001163672	49747832	49757864	49690173	49729844	-	-100k	+	AS	mitogen activated protein kinase 9 isoform
**222**	UID205	chr11	NM_029004	49747832	49757864	49745257	49823646	-	-5k	+	AS	RasGEF domain family, member 1C
**223**	UID205	chr11	NM_016961	49747832	49757864	49690173	49729844	-	-100k	+	AS	mitogen activated protein kinase 9 isoform
**224**	UID205	chr11	NM_207692	49747832	49757864	49690173	49729844	-	-100k	+	AS	mitogen activated protein kinase 9 isoform
**225**	UID205	chr11	NM_001163671	49747832	49757864	49690173	49729844	-	-100k	+	AS	mitogen activated protein kinase 9 isoform
**226**	UID206	chr11	NM_001161356	46602189	46612630	46512382	46533780	-	-100k	-	AS	T-cell immunoglobulin and mucin domain
**227**	UID206	chr11	NM_001161355	46602189	46612630	46512382	46542253	-	-100k	-	AS	T-cell immunoglobulin and mucin domain
**228**	UID206	chr11	NM_178759	46602189	46612630	46654221	46687756	-	+100k	+	AS	T-cell immunoglobulin and mucin domain
**229**	UID206	chr11	NM_134248	46602189	46612630	46594599	46622036	-	-10k	+	AS	hepatitis A virus cellular receptor 1 precursor
**230**	UID206	chr11	NM_134249	46602189	46612630	46512382	46550527	-	-100k	-	AS	T-cell immunoglobulin and mucin domain
**231**	UID208	chr11	NM_008073	41720465	41730906	41755279	41844101	-	+50k	-	S	gamma-aminobutyric acid (GABA) A receptor,
**232**	UID208	chr11	NM_177408	41720465	41730906	41753617	41843742	-	+50k	-	S	gamma-aminobutyric acid (GABA) A receptor,
**233**	UID209	chr11	NM_009831	40681174	40691572	40591974	40598709	-	-100k	-	AS	cyclin G1
**234**	UID210	chr11	NM_026023	40646544	40657030	40577064	40583469	-	-100k	+	AS	NudC domain containing 2
**235**	UID210	chr11	NM_009831	40646544	40657030	40591974	40598709	-	-100k	-	AS	cyclin G1
**236**	UID214	chr11	NM_176841	29224874	29235360	29273774	29410811	-	+50k	+	AS	coiled coil domain containing 88A
**237**	UID215	chr11	NM_181577	28246392	28256833	28288814	28484296	-	+50k	-	S	coiled-coil domain containing 85A
**238**	UID219	chr11	NM_009044	23733755	23744196	23641728	23670970	-	-100k	-	AS	reticuloendotheliosis oncogene
**239**	UID219	chr11	NM_172555	23733755	23744196	23762646	23795270	-	+50k	-	S	poly(A) polymerase gamma
**240**	UID223	chr11	NM_207655	16587902	16598299	16652205	16813910	-	+100k	+	AS	epidermal growth factor receptor isoform 1
**241**	UID223	chr11	NM_007912	16587902	16598299	16652205	16787661	-	+100k	+	AS	epidermal growth factor receptor isoform 2
**242**	UID225	chr11	NM_172496	12038497	12048894	12136678	12364963	-	+100k	-	S	cordon-bleu protein
**243**	UID228	chr2	NR_027890	6754503	6764944	6839973	6845994	+	+100k	-	AS	
**244**	UID232	chr2	NM_008859	11036920	11047361	11090234	11219079	+	+100k	+	AS	protein kinase C, theta
**245**	UID233	chr2	NM_011701	13573292	13583733	13492163	13500679	+	-100k	+	S	vimentin
**246**	UID233	chr2	NM_145838	13573292	13583733	13572790	13711274	+	-5k	-	AS	sialyltransferase 8 F
**247**	UID237	chr2	NM_001001297	21161384	21171617	21123477	21132763	+	-50k	+	S	threonine synthase-like 1 isoform b
**248**	UID237	chr2	NM_177588	21161384	21171617	21123477	21132763	+	-50k	+	S	threonine synthase-like 1 isoform a
**249**	UID237	chr2	NM_027728	21161384	21171617	21098484	21123119	+	-100k	-	AS	enkurin
**250**	UID239	chr2	NM_080462	23758958	23769399	23824921	23871388	+	+100k	-	AS	histamine N-methyltransferase
**251**	UID240	chr2	NM_080462	23764451	23774892	23824921	23871388	+	+100k	-	AS	histamine N-methyltransferase
**252**	UID241	chr2	NM_080462	23769945	23779785	23824921	23871388	+	+100k	-	AS	histamine N-methyltransferase

**253**	UID242	chr2	NM_146622	36927581	36938022	36890315	36891269	+	-50k	+	S	olfactory receptor 360
**254**	UID242	chr2	NM_146368	36927581	36938022	36906786	36907755	+	-50k	-	AS	olfactory receptor 361
**255**	UID242	chr2	NM_147051	36927581	36938022	36926703	36927657	+	-5k	-	AS	olfactory receptor 362
**256**	UID242	chr2	NM_146662	36927581	36938022	37023251	37024190	+	+100k	+	AS	olfactory receptor 365
**257**	UID246	chr2	NM_015753	44755033	44765519	44805642	44935288	+	+100k	-	AS	zinc finger homeobox 1b isoform 2
**258**	UID249	chr2	NM_173030	54197901	54208342	54251772	54933130	+	+100k	+	AS	UDP-N-acetyl-alpha-D-galactosamine:polypeptide
**259**	UID252	chr2	NM_008426	55304291	55314732	55252541	55410911	+	-100k	+	S	potassium inwardly-rectifying channel, subfamily
**260**	UID255	chr2	NM_001111030	58069146	58079544	58082838	58173144	+	+20k	-	AS	activin A receptor, type IC isoform 1
**261**	UID255	chr2	NM_001033369	58069146	58079544	58082838	58140193	+	+20k	-	AS	activin A receptor, type IC isoform 2
**262**	UID259	chr2	NM_153409	65572926	65583367	65646605	65824323	+	+100k	+	AS	cysteine-serine-rich nuclear protein 3
**263**	UID259	chr2	NM_178634	65572926	65583367	65646605	65824323	+	+100k	+	AS	cysteine-serine-rich nuclear protein 3
**264**	UID260	chr2	NM_172856	68568187	68578628	68662395	68912129	+	+100k	+	AS	LAG1 homolog, ceramide synthase 6
**265**	UID265	chr2	NM_024181	80049187	80059628	80116304	80154894	+	+100k	+	AS	DnaJ (Hsp40) homolog, subfamily C, member 10
**266**	UID265	chr2	NM_177256	80049187	80059628	80093310	80096195	+	+50k	+	AS	peroxiredoxin 5 related sequence 1 protein
**267**	UID268	chr2	NM_146847	86640979	86651377	86554633	86555575	+	-100k	-	AS	olfactory receptor 1090
**268**	UID268	chr2	NM_146366	86640979	86651377	86586570	86587539	+	-100k	+	S	olfactory receptor 1093
**269**	UID268	chr2	NM_181818	86640979	86651377	86606859	86607836	+	-50k	-	AS	olfactory receptor 141
**270**	UID268	chr2	NM_146365	86640979	86651377	86629592	86630585	+	-20k	+	S	olfactory receptor 1094
**271**	UID268	chr2	NM_146730	86640979	86651377	86651608	86652535	+	+20k	-	AS	olfactory receptor 1095
**272**	UID268	chr2	NM_146843	86640979	86651377	86691064	86692012	+	+100k	-	AS	olfactory receptor 1097
**273**	UID268	chr2	NM_146845	86640979	86651377	86723421	86724369	+	+100k	-	AS	olfactory receptor 1098
**274**	UID269	chr2	NM_146294	88039049	88049493	87949905	87950856	+	-100k	-	AS	olfactory receptor 1167
**275**	UID269	chr2	NM_146531	88039049	88049493	87985717	87986656	+	-100k	+	S	olfactory receptor 1168
**276**	UID269	chr2	NM_146532	88039049	88049493	88024918	88025869	+	-20k	-	AS	olfactory receptor 1170
**277**	UID269	chr2	NM_207566	88039049	88049493	88074947	88075886	+	+50k	-	AS	olfactory receptor 1173
**278**	UID270	chr2	NM_021368	89885795	89896236	89821976	89822903	+	-100k	-	AS	olfactory receptor 1264
**279**	UID270	chr2	NM_020515	89885795	89896236	89852252	89853161	+	-50k	-	AS	olfactory receptor 140
**280**	UID270	chr2	NM_146474	89885795	89896236	89794233	89795154	+	-100k	+	S	olfactory receptor 1261
**281**	UID270	chr2	NM_146974	89885795	89896236	89803246	89804161	+	-100k	+	S	olfactory receptor 1262
**282**	UID270	chr2	NM_146794	89885795	89896236	89815770	89816691	+	-100k	+	S	olfactory receptor 1263
**283**	UID270	chr2	NM_146343	89885795	89896236	89837759	89838689	+	-50k	+	S	olfactory receptor 1265
**284**	UID270	chr2	NM_146342	89885795	89896236	89919505	89920435	+	+50k	-	AS	olfactory receptor 1269
**285**	UID270	chr2	NM_010980	89885795	89896236	89939049	89939976	+	+100k	-	AS	olfactory receptor 32
**286**	UID270	chr2	NM_146985	89885795	89896236	89949928	89950843	+	+100k	-	AS	olfactory receptor 1270
**287**	UID271	chr2	NM_146263	90171336	90181777	90201501	90202431	+	+50k	+	AS	olfactory receptor 1274
**288**	UID271	chr2	NM_146980	90171336	90181777	90082485	90083412	+	-100k	-	AS	olfactory receptor 1272
**289**	UID271	chr2	NM_146975	90171336	90181777	90096774	90097698	+	-100k	-	AS	olfactory receptor 1273
**290**	UID271	chr2	NM_146265	90171336	90181777	90125035	90125965	+	-50k	+	S	olfactory receptor 1506
**291**	UID271	chr2	NM_008982	90171336	90181777	90233728	90381486	+	+100k	-	AS	protein tyrosine phosphatase, receptor type, J
**292**	UID271	chr2	NM_001135657	90171336	90181777	90230594	90280013	+	+100k	-	AS	protein tyrosine phosphatase, receptor type, J
**293**	UID276	chr2	NM_026592	101279367	101289808	101361619	101429825	+	+100k	-	AS	hypothetical protein LOC68170
**294**	UID277	chr2	NM_028260	105656743	105666975	105705476	105766397	+	+50k	+	AS	IMP1 inner mitochondrial membrane
**295**	UID279	chr2	NM_026613	109902431	109912663	109818655	109846162	+	-100k	+	S	coiled-coil domain containing 34

**296**	UID280	chr2	NM_130452	110035030	110045471	110065921	110106564	+	+50k	-	AS	gamma-butyrobetaine dioxygenase
**297**	UID281	chr2	NM_001003915	110343277	110353718	110398136	110448608	+	+100k	+	AS	solute carrier family 5 (sodium/glucose
**298**	UID282	chr2	NM_025675	114185997	114196438	114207858	114346369	+	+50k	-	AS	ATP binding domain 4
**299**	UID285	chr2	NM_001004174	122332210	122342651	122329327	122332517	+	-5k	+	S	normal mucosa of esophagus specific 1
**300**	UID285	chr2	NM_019788	122332210	122342651	122429945	122440928	+	+100k	+	AS	pallidin
**301**	UID285	chr2	NM_025961	122332210	122342651	122285913	122302718	+	-50k	-	AS	L-arginine:glycine amidinotransferase precursor
**302**	UID285	chr2	NM_011774	122332210	122342651	122372679	122394104	+	+50k	-	AS	solute carrier family 30 (zinc transporter),
**303**	UID291	chr2	NM_009378	148149069	148159512	148095911	148099629	+	-100k	-	AS	thrombomodulin precursor
**304**	UID291	chr2	NM_010740	148149069	148159512	148128091	148134976	+	-50k	-	AS	CD93 antigen
**305**	UID291	chr2	NM_009219	148149069	148159512	148086817	148088205	+	-100k	+	S	somatostatin receptor 4
**306**	UID293	chr2	NM_175631	171696111	171706552	171727540	171734671	+	+50k	-	AS	cerebellin 4 precursor
**307**	UID294	chr2	NM_001034900	150129373	150139814	150162431	150176504	+	+50k	-	AS	zinc finger protein 345
**308**	UID295	chr2	NM_008953	153776377	153786818	153699716	153707514	+	-100k	+	S	parotid secretory protein precursor
**309**	UID295	chr2	NM_028528	153776377	153786818	153821787	153829796	+	+50k	+	AS	Splunc3
**310**	UID295	chr2	NM_011126	153776377	153786818	153834320	153840657	+	+100k	+	AS	palate, lung, and nasal epithelium clone
**311**	UID295	chr2	NM_025990	153776377	153786818	153854048	153859884	+	+100k	+	AS	RIKEN cDNA 2310021H06 precursor
**312**	UID303	chr2	NM_175631	171696111	171706552	171727540	171734671	+	+50k	-	AS	cerebellin 4 precursor
**313**	UID304	chr2	NM_009236	181668181	181678579	181599242	181601046	-	-100k	-	AS	SRY-box containing gene 18
**314**	UID304	chr2	NM_009326	181668181	181678579	181609715	181617457	-	-100k	+	AS	transcription elongation factor A protein 2
**315**	UID304	chr2	NM_008665	181668181	181678579	181696447	181757174	-	+50k	+	AS	myelin transcription factor 1
**316**	UID304	chr2	NM_011012	181668181	181678579	181644449	181650378	-	-50k	+	AS	opioid receptor-like 1
**317**	UID304	chr2	NM_026446	181668181	181678579	181617828	181623330	-	-100k	-	AS	regulator of G-protein signaling 19
**318**	UID304	chr2	NM_199023	181668181	181678579	181626200	181627848	-	-50k	-	AS	hypothetical protein LOC277496
**319**	UID304	chr2	NM_153594	181668181	181678579	181767308	181786856	-	+100k	+	AS	protein-L-isoaspartate (D-aspartate)
**320**	UID305	chr2	NM_177191	178370657	178381091	178274703	178337066	-	-100k	-	AS	synaptonemal complex protein 2
**321**	UID305	chr2	NM_001081289	178370657	178381091	178343941	178351569	-	-50k	+	AS	hypothetical protein LOC71532
**322**	UID305	chr2	NM_198656	178370657	178381091	178360014	178416774	-	-20k	+	AS	cadherin-like 26 precursor
**323**	UID305	chr2	NM_001085501	178370657	178381091	178340613	178343880	-	-50k	-	AS	protein phosphatase 1, regulatory subunit 3D
**324**	UID306	chr2	NM_053195	144926715	144937147	144934051	145333380	-	+10k	+	AS	solute carrier family 24
**325**	UID308	chr2	NM_178382	140423731	140434129	140349641	140362910	-	-100k	-	AS	fibronectin leucine rich transmembrane protein
**326**	UID312	chr2	NM_028201	136554143	136564626	136582658	136761090	-	+50k	+	AS	hypothetical protein LOC74243 isoform 2
**327**	UID312	chr2	NM_028834	136554143	136564626	136583703	136759834	-	+50k	+	AS	hypothetical protein LOC74243 isoform 1
**328**	UID312	chr2	NM_001038641	136554143	136564626	136590791	136761219	-	+50k	+	AS	hypothetical protein LOC74243 isoform 1
**329**	UID312	chr2	NM_021527	136554143	136564626	136565220	136582847	-	+20k	-	S	McKusick-Kaufman syndrome protein
**330**	UID312	chr2	NM_001141946	136554143	136564626	136565220	136582847	-	+20k	-	S	McKusick-Kaufman syndrome protein
**331**	UID322	chr2	NM_026271	110228876	110239317	110161763	110163832	-	-100k	-	AS	fin bud initiation factor homolog precursor
**332**	UID324	chr2	NM_001048141	109429182	109439580	109493274	109527882	-	+100k	+	AS	brain-derived neurotrophic factor isoform 2
**333**	UID324	chr2	NM_007540	109429182	109439580	109475538	109527882	-	+50k	+	AS	brain-derived neurotrophic factor isoform 1
**334**	UID324	chr2	NM_001048142	109429182	109439580	109494401	109527882	-	+100k	+	AS	brain-derived neurotrophic factor isoform 2
**335**	UID324	chr2	NM_001048139	109429182	109439580	109476735	109527882	-	+50k	+	AS	brain-derived neurotrophic factor isoform 2
**336**	UID325	chr2	NM_029790	108897617	108908013	108893138	109079129	-	-5k	-	AS	methyltransferase 5 domain containing 1
**337**	UID328	chr2	NM_008045	106933914	106944312	106856979	106860490	-	-100k	-	AS	follicle stimulating hormone beta precursor
**338**	UID329	chr2	NM_008045	106900694	106911092	106856979	106860490	-	-50k	-	AS	follicle stimulating hormone beta precursor

**339**	UID330	chr2	NM_001111060	103809041	103819482	103896639	103916190	-	+100k	+	AS	CD59a antigen precursor
**340**	UID330	chr2	NM_181858	103809041	103819482	103871848	103885796	-	+100k	+	AS	CD59b antigen precursor
**341**	UID330	chr2	NM_008505	103809041	103819482	103771125	103782716	-	-50k	+	AS	LIM domain only 2 isoform 1
**342**	UID330	chr2	NM_020593	103809041	103819482	103828637	103855966	-	+20k	+	AS	F-box only protein 3 isoform 2
**343**	UID330	chr2	NM_212433	103809041	103819482	103828637	103864076	-	+20k	+	AS	F-box only protein 3 isoform 1
**344**	UID330	chr2	NM_007652	103809041	103819482	103896639	103916190	-	+100k	+	AS	CD59a antigen precursor
**345**	UID330	chr2	NM_001142336	103809041	103819482	103770395	103782716	-	-50k	+	AS	LIM domain only 2 isoform 3
**346**	UID330	chr2	NM_001142335	103809041	103819482	103771125	103782716	-	-50k	+	AS	LIM domain only 2 isoform 2
**347**	UID330	chr2	NM_001142337	103809041	103819482	103758833	103782716	-	-100k	+	AS	LIM domain only 2 isoform 3
**348**	UID339	chr2	NM_146985	90042544	90052985	89949928	89950843	-	-100k	-	AS	olfactory receptor 1270
**349**	UID339	chr2	NM_146265	90042544	90052985	90022405	90023335	-	-50k	-	AS	olfactory receptor 1506
**350**	UID339	chr2	NM_146984	90042544	90052985	90052907	90053825	-	+20k	-	S	olfactory receptor 142
**351**	UID339	chr2	NM_146793	90042544	90052985	90066349	90067267	-	+50k	-	S	olfactory receptor 1271
**352**	UID339	chr2	NM_146980	90042544	90052985	90082485	90083412	-	+50k	-	S	olfactory receptor 1272
**353**	UID339	chr2	NM_146975	90042544	90052985	90096774	90097698	-	+100k	-	S	olfactory receptor 1273
**354**	UID339	chr2	NM_146265	90042544	90052985	90125035	90125965	-	+100k	+	AS	olfactory receptor 1506
**355**	UID340	chr2	NM_146342	89966593	89977034	89919505	89920435	-	-50k	-	AS	olfactory receptor 1269
**356**	UID340	chr2	NM_010980	89966593	89977034	89939049	89939976	-	-50k	-	AS	olfactory receptor 32
**357**	UID340	chr2	NM_146985	89966593	89977034	89949928	89950843	-	-20k	-	AS	olfactory receptor 1270
**358**	UID340	chr2	NM_146265	89966593	89977034	90022405	90023335	-	+100k	-	S	olfactory receptor 1506
**359**	UID340	chr2	NM_146984	89966593	89977034	90052907	90053825	-	+100k	-	S	olfactory receptor 142
**360**	UID340	chr2	NM_146793	89966593	89977034	90066349	90067267	-	+100k	-	S	olfactory receptor 1271
**361**	UID341	chr2	NM_146977	89692920	89703364	89617166	89618099	-	-100k	+	AS	olfactory receptor 1255
**362**	UID341	chr2	NM_146983	89692920	89703364	89635861	89636782	-	-100k	-	AS	olfactory receptor 1256
**363**	UID341	chr2	NM_010990	89692920	89703364	89644904	89645810	-	-50k	-	AS	olfactory receptor 48
**364**	UID341	chr2	NM_146982	89692920	89703364	89681666	89682596	-	-20k	+	AS	olfactory receptor 1257
**365**	UID341	chr2	NM_146978	89692920	89703364	89730649	89731585	-	+50k	+	AS	olfactory receptor 1258
**366**	UID341	chr2	NM_146341	89692920	89703364	89744022	89744952	-	+100k	-	S	olfactory receptor 1259
**367**	UID341	chr2	NM_146981	89692920	89703364	89778618	89779551	-	+100k	+	AS	olfactory receptor 1260
**368**	UID342	chr2	NM_001011517	88504897	88515338	88478695	88479652	-	-50k	+	AS	olfactory receptor 1193
**369**	UID342	chr2	NM_146753	88504897	88515338	88483642	88484569	-	-50k	-	AS	olfactory receptor 1195
**370**	UID342	chr2	NM_146464	88504897	88515338	88501221	88502166	-	-5k	-	AS	olfactory receptor 1196
**371**	UID342	chr2	NM_001005225	88504897	88515338	88529491	88530436	-	+50k	-	S	olfactory receptor 1197
**372**	UID342	chr2	NM_207567	88504897	88515338	88546798	88547725	-	+50k	-	S	olfactory receptor 1198
**373**	UID342	chr2	NM_146458	88504897	88515338	88556579	88557512	-	+100k	-	S	olfactory receptor 1199
**374**	UID342	chr2	NM_001005227	88504897	88515338	88568141	88569152	-	+100k	-	S	olfactory receptor 1200
**375**	UID342	chr2	NM_146895	88504897	88515338	88595222	88596146	-	+100k	+	AS	olfactory receptor 1201
**376**	UID343	chr2	NM_146772	88463997	88474438	88392644	88393565	-	-100k	+	AS	olfactory receptor 1189
**377**	UID343	chr2	NM_001011517	88463997	88474438	88478695	88479652	-	+20k	+	AS	olfactory receptor 1193
**378**	UID343	chr2	NM_146753	88463997	88474438	88483642	88484569	-	+20k	-	S	olfactory receptor 1195
**379**	UID343	chr2	NM_146464	88463997	88474438	88501221	88502166	-	+50k	-	S	olfactory receptor 1196
**380**	UID343	chr2	NM_001005225	88463997	88474438	88529491	88530436	-	+100k	-	S	olfactory receptor 1197
**381**	UID343	chr2	NM_207567	88463997	88474438	88546798	88547725	-	+100k	-	S	olfactory receptor 1198

**382**	UID343	chr2	NM_146458	88463997	88474438	88556579	88557512	-	+100k	-	S	olfactory receptor 1199
**383**	UID344	chr2	NM_001011785	85094053	85104536	85131805	85132735	-	+50k	-	S	olfactory receptor 987
**384**	UID344	chr2	NM_001011534	85094053	85104536	85153833	85154763	-	+100k	-	S	olfactory receptor 988
**385**	UID345	chr2	NM_145526	84878368	84888851	84797390	84836673	-	-100k	-	AS	purinergic receptor P2X3
**386**	UID345	chr2	NM_008920	84878368	84888851	84781299	84784471	-	-100k	+	AS	proteoglycan 2, bone marrow precursor
**387**	UID345	chr2	NM_016914	84878368	84888851	84789053	84794725	-	-100k	+	AS	proteoglycan 3 precursor
**388**	UID345	chr2	NM_011784	84878368	84888851	84937198	84940761	-	+100k	+	AS	apelin receptor
**389**	UID345	chr2	NM_182990	84878368	84888851	84838039	84847950	-	-50k	+	AS	structure specific recognition protein 1 isoform
**390**	UID345	chr2	NM_001081260	84878368	84888851	84851298	84873887	-	-50k	+	AS	tankyrase 1 binding protein 1
**391**	UID345	chr2	NM_001136081	84878368	84888851	84838289	84847945	-	-50k	+	AS	structure specific recognition protein 1 isoform
**392**	UID346	chr2	NM_175514	83686760	83697154	83613566	83682197	-	-100k	+	AS	family with sequence similarity 171, member B
**393**	UID346	chr2	NM_027964	83686760	83697154	83715917	83742065	-	+50k	-	S	zinc finger, SWIM domain containing 2
**394**	UID353	chr2	NM_177843	77940007	77950405	78038385	78103069	-	+100k	+	AS	hypothetical protein LOC329436
**395**	UID354	chr2	NM_001033477	66883411	66893897	66974672	66987051	-	+100k	+	AS	hypothetical protein LOC383709
**396**	UID360	chr2	NM_029692	58499883	58510324	58570570	58606054	-	+100k	+	AS	uridine phosphorylase 2
**397**	UID368	chr2	NM_172663	49296330	49306771	49273494	49373618	-	-50k	+	AS	enhancer of polycomb homolog 2
**398**	UID381	chr2	NM_024209	39061532	39071973	39018806	39048347	-	-50k	-	AS	protein phosphatase 6, catalytic subunit
**399**	UID382	chr2	NM_146951	36363814	36374212	36274595	36275534	-	-100k	+	AS	olfactory receptor 340
**400**	UID382	chr2	NM_146950	36363814	36374212	36301195	36302137	-	-100k	-	AS	olfactory receptor 341
**401**	UID382	chr2	NM_146948	36363814	36374212	36349422	36350361	-	-20k	+	AS	olfactory receptor 342
**402**	UID382	chr2	NM_146628	36363814	36374212	36390608	36391538	-	+50k	+	AS	olfactory receptor 344
**403**	UID382	chr2	NM_146945	36363814	36374212	36462049	36462985	-	+100k	+	AS	olfactory receptor 345
**404**	UID383	chr2	NM_146947	36258293	36268691	36198786	36199707	-	-100k	+	AS	olfactory receptor 338
**405**	UID383	chr2	NM_146949	36258293	36268691	36243408	36244338	-	-20k	+	AS	olfactory receptor 339
**406**	UID383	chr2	NM_146951	36258293	36268691	36274595	36275534	-	+20k	+	AS	olfactory receptor 340
**407**	UID383	chr2	NM_146950	36258293	36268691	36301195	36302137	-	+50k	-	S	olfactory receptor 341
**408**	UID383	chr2	NM_146948	36258293	36268691	36349422	36350361	-	+100k	+	AS	olfactory receptor 342
**409**	UID384	chr2	NM_026896	29119327	29129768	29168844	29346797	-	+50k	+	AS	transcriptional co-activator CRSP8 homolog
**410**	UID385	chr2	NM_148413	22075192	22085636	22158821	22469894	-	+100k	+	AS	myosin IIIA
**411**	UID391	chr2	NM_026162	16348513	16358954	16274075	16669869	-	-100k	+	AS	plexin domain containing 2 precursor
**412**	UID392	chr2	NM_026162	16291976	16302417	16274075	16669869	-	-20k	+	AS	plexin domain containing 2 precursor
**413**	UID394	chr2	NM_008625	14198956	14209397	14147167	14249776	-	-100k	+	AS	mannose receptor, C type 1 precursor
**414**	UID395	chr2	NM_001012396	14035575	14046058	13944584	13973789	-	-100k	-	AS	protein tyrosine phosphatase-like (proline
**415**	UID395	chr2	NM_013935	14035575	14046058	13944584	13973789	-	-100k	-	AS	protein tyrosine phosphatase-like (proline
**416**	UID395	chr2	NM_011484	14035575	14046058	13991865	14066084	-	-50k	+	AS	signal transducing adaptor molecule (SH3 domain)
**417**	UID395	chr2	NM_001081310	14035575	14046058	14092277	14139747	-	+100k	+	AS	hypothetical protein LOC625286
**418**	UID401	chr15	NM_199476	37801959	37812400	37868541	37905644	+	+100k	-	AS	ribonucleotide reductase M2 B (TP53 inducible)
**419**	UID405	chr15	NM_001081391	47375922	47386405	47410711	48622063	+	+50k	-	AS	CUB and Sushi multiple domains 3
**420**	UID412	chr15	NM_133766	65542630	65553030	65616713	65703485	+	+100k	+	AS	RIKEN cDNA C920006C10
**421**	UID413	chr15	NM_133766	65564653	65575094	65616713	65703485	+	+100k	+	AS	RIKEN cDNA C920006C10
**422**	UID416	chr15	NM_026817	89484602	89494999	89410293	89419690	+	-100k	-	AS	RAB, member of RAS oncogene family-like 2A
**423**	UID416	chr15	NM_013455	89484602	89494999	89396092	89402351	+	-100k	+	S	acrosin precursor
**424**	UID417	chr15	NM_001033441	90017674	90028114	90052077	90058321	+	+50k	+	AS	expressed sequence AU045404

**425**	UID418	chr15	NM_001003670	101480729	101491170	101452061	101456222	+	-50k	-	AS	type II keratin Kb14
**426**	UID418	chr15	NM_010669	101480729	101491170	101504056	101508323	+	+50k	-	AS	keratin complex 2, basic, gene 6b
**427**	UID418	chr15	NM_008476	101480729	101491170	101517961	101522339	+	+50k	-	AS	keratin 6A
**428**	UID418	chr15	NM_019956	101480729	101491170	101561982	101571131	+	+100k	-	AS	keratin 71
**429**	UID418	chr15	NM_027011	101480729	101491170	101535103	101540925	+	+100k	-	AS	keratin 5
**430**	UID418	chr15	NM_133357	101480729	101491170	101391376	101401938	+	-100k	-	AS	keratin 75
**431**	UID418	chr15	NR_002869	101480729	101491170	101436907	101438513	+	-50k	+	S	
**432**	UID418	chr15	NR_003960	101480729	101491170	101471053	101475414	+	-10k	-	AS	
**433**	UID418	chr15	NR_002868	101480729	101491170	101415350	101416606	+	-100k	-	AS	
**434**	UID419	chr15	NM_013866	103237149	103247590	103141928	103168120	+	-100k	-	AS	zinc finger protein 385A
**435**	UID419	chr15	NM_008800	103237149	103247590	103331331	103358086	+	+100k	+	AS	phosphodiesterase 1B
**436**	UID419	chr15	NM_153505	103237149	103247590	103281858	103326834	+	+50k	+	AS	NCK associated protein 1 like
**437**	UID419	chr15	NM_177631	103237149	103247590	103266993	103276493	+	+50k	-	AS	hypothetical protein LOC223927
**438**	UID419	chr15	NM_010577	103237149	103247590	103172322	103194782	+	-100k	-	AS	integrin alpha 5 preproprotein
**439**	UID419	chr15	NM_028797	103237149	103247590	103230494	103258461	+	-10k	-	AS	gametocyte specific factor 1
**440**	UID421	chr15	NM_177431	94122605	94132999	94098342	94232438	-	-50k	-	AS	a disintegrin-like and metalloprotease with
**441**	UID423	chr15	NM_177820	77324726	77335152	77411412	77423380	-	+100k	-	S	apolipoprotein L 10b
**442**	UID423	chr15	NM_001024848	77324726	77335152	77249463	77274715	-	-100k	-	AS	apolipoprotein L 7b
**443**	UID423	chr15	NM_177744	77324726	77335152	77304301	77318324	-	-50k	+	AS	apolipoprotein L 10a
**444**	UID423	chr15	NM_175391	77324726	77335152	77352121	77360570	-	+50k	-	S	apolipoprotein L 7c
**445**	UID423	chr15	NM_001162883	77324726	77335152	77231044	77238335	-	-100k	-	AS	apolipoprotein L 9a
**446**	UID423	chr15	NM_173786	77324726	77335152	77231044	77238289	-	-100k	-	AS	apolipoprotein L 9a
**447**	UID424	chr15	NM_027668	59887578	59897976	59844546	59846299	-	-50k	-	AS	hypothetical protein LOC71088
**448**	UID425	chr15	NM_008764	54033784	54044225	54080701	54108567	-	+50k	-	S	tumor necrosis factor receptor superfamily,
**449**	UID431	chr15	NM_001085421	33682447	33692888	33628463	33632472	-	-100k	-	AS	testis-specific protein, Y-encoded-like 5
**450**	UID436	chr15	NM_177355	4362922	4373363	4325490	4525579	-	-50k	+	AS	phosphatidylinositol-specific phospholipase C, X
**451**	UID437	chr1	NM_010342	5807250	5817691	5903788	5907479	+	+100k	-	AS	G protein-coupled receptor 7
**452**	UID441	chr1	NM_175642	25061569	25072007	25012021	25774253	+	-50k	-	AS	brain-specific angiogenesis inhibitor 3
**453**	UID449	chr1	NM_144953	52881512	52891953	52869671	52897385	+	-20k	-	AS	hypothetical protein LOC67080
**454**	UID449	chr1	NM_146108	52881512	52891953	52789590	52865405	+	-100k	+	S	3-hydroxyisobutyryl-Coenzyme A hydrolase
**455**	UID450	chr1	NM_001033194	54328823	54339264	54342125	54383516	+	+20k	-	AS	general transcription factor IIIC, polypeptide
**456**	UID450	chr1	NM_001163314	54328823	54339264	54425250	54502227	+	+100k	-	AS	GPI deacylase
**457**	UID451	chr1	NM_007642	60629144	60639587	60690933	60717905	+	+100k	+	AS	CD28 antigen precursor
**458**	UID453	chr1	NM_001081050	61602207	61612605	61573099	62576560	+	-50k	+	S	par-3 partitioning defective 3 homolog B
**459**	UID458	chr1	NM_175210	71180548	71190985	71176297	71348118	+	-5k	-	AS	ATP-binding cassette, sub-family A (ABC1),
**460**	UID459	chr1	NM_009129	79256909	79267350	79313692	79319114	+	+100k	-	AS	secretogranin II precursor
**461**	UID462	chr1	NM_152915	84327845	84338243	84248863	84575246	+	-100k	-	AS	delta/notch-like EGF-related receptor precursor
**462**	UID466	chr1	NM_029349	107392524	107402965	107491407	107582678	+	+100k	+	AS	hypothetical protein LOC227446 isoform 2
**463**	UID466	chr1	NM_013784	107392524	107402965	107348686	107491223	+	-50k	-	AS	phosphatidylinositol glycan anchor biosynthesis,
**464**	UID466	chr1	NM_173187	107392524	107402965	107491407	107582678	+	+100k	+	AS	hypothetical protein LOC227446 isoform 1
**465**	UID470	chr1	NM_019933	121568923	121579364	121485638	121664617	+	-100k	-	AS	protein tyrosine phosphatase, non-receptor type
**466**	UID473	chr1	NM_177243	133505111	133515552	133571567	133597951	+	+100k	+	AS	solute carrier family 26, member 9
**467**	UID473	chr1	NM_145509	133505111	133515552	133516240	133541010	+	+20k	+	AS	RAB7B, member RAS oncogene family

**468**	UID473	chr1	NM_007799	133505111	133515552	133465859	133503053	+	-50k	+	S	cathepsin E preproprotein
**469**	UID473	chr1	NM_011924	133505111	133515552	133426659	133439546	+	-100k	+	S	arginine vasopressin receptor 1B
**470**	UID475	chr1	NM_001159866	145621532	145631973	145539495	145569684	+	-100k	+	S	ubiquitin carboxyl-terminal hydrolase L5 isoform
**471**	UID475	chr1	NM_019562	145621532	145631973	145539495	145569684	+	-100k	+	S	ubiquitin carboxyl-terminal hydrolase L5 isoform
**472**	UID476	chr1	NM_009061	145824468	145834869	145761555	145766367	+	-100k	-	AS	regulator of G-protein signaling 2
**473**	UID476	chr1	NM_153171	145824468	145834869	145900884	145939590	+	+100k	-	AS	regulator of G-protein signaling 13
**474**	UID477	chr1	NM_022881	146566112	146576510	146515058	146537639	+	-100k	-	AS	regulator of G-protein signaling 18
**475**	UID478	chr1	NM_153539	148204686	148215081	148257883	148664690	+	+100k	+	AS	family with sequence similarity 5, member C
**476**	UID478	chr1	NM_001145807	148204686	148215081	148259904	148664690	+	+100k	+	AS	family with sequence similarity 5, member C
**477**	UID482	chr1	NM_024458	151992226	152002666	152081634	152096123	+	+100k	+	AS	phosducin
**478**	UID482	chr1	NM_001159730	151992226	152002666	152081634	152096123	+	+100k	+	AS	phosducin
**479**	UID487	chr1	NM_010778	196749422	196759820	196742623	196792972	-	-10k	-	AS	CD46 antigen, complement regulatory protein
**480**	UID487	chr1	NM_007758	196749422	196759820	196837534	196877439	-	+100k	-	S	complement receptor 2
**481**	UID487	chr1	NM_013499	196749422	196759820	196804511	196832294	-	+100k	-	S	complement receptor related protein
**482**	UID488	chr1	NM_146106	187803943	187814384	187788519	187818098	-	-20k	-	AS	lysophospholipase-like 1
**483**	UID494	chr1	NM_001029977	141459541	141469941	141514329	141597650	-	+100k	-	S	complement factor H-related protein C isoform 1
**484**	UID494	chr1	NM_015780	141459541	141469941	141363474	141376633	-	-100k	-	AS	complement factor H-related 1
**485**	UID494	chr1	NM_001160303	141459541	141469941	141514329	141597650	-	+100k	-	S	complement factor H-related protein C isoform 2
**486**	UID494	chr1	NM_001160304	141459541	141469941	141514329	141597650	-	+100k	-	S	complement factor H-related protein C isoform 3
**487**	UID498	chr1	NM_027971	108675396	108685837	108761995	108784625	-	+100k	+	AS	serine (or cysteine) proteinase inhibitor, clade
**488**	UID498	chr1	NM_009257	108675396	108685837	108688726	108710895	-	+20k	+	AS	serine (or cysteine) proteinase inhibitor, clade
**489**	UID498	chr1	NM_009190	108675396	108685837	108598334	108624272	-	-100k	-	AS	vacuolar protein sorting 4b
**490**	UID506	chr1	NM_172422	63763229	63773627	63664900	63687661	-	-100k	+	AS	FAST kinase domains 2
**491**	UID509	chr1	NM_001077406	62604615	62615056	62637592	62731367	-	+50k	+	AS	neuropilin 2 isoform 5 precursor
**492**	UID509	chr1	NM_001077404	62604615	62615056	62637592	62752968	-	+50k	+	AS	neuropilin 2 isoform 2 precursor
**493**	UID509	chr1	NM_001077405	62604615	62615056	62637592	62752968	-	+50k	+	AS	neuropilin 2 isoform 4 precursor
**494**	UID509	chr1	NM_010939	62604615	62615056	62637592	62752968	-	+50k	+	AS	neuropilin 2 isoform 3 precursor
**495**	UID509	chr1	NM_001077407	62604615	62615056	62637592	62731367	-	+50k	+	AS	neuropilin 2 isoform 6 precursor
**496**	UID509	chr1	NM_001077403	62604615	62615056	62637592	62752968	-	+50k	+	AS	neuropilin 2 isoform 1 precursor
**497**	UID512	chr1	NM_144953	52831261	52841697	52869671	52897385	-	+50k	-	S	hypothetical protein LOC67080
**498**	UID512	chr1	NM_146108	52831261	52841697	52789590	52865405	-	-50k	+	AS	3-hydroxyisobutyryl-Coenzyme A hydrolase
**499**	UID512	chr1	NM_008384	52831261	52841697	52733964	52762233	-	-100k	-	AS	inositol polyphosphate-1-phosphatase
**500**	UID520	chr1	NM_001025305	19109931	19120329	19197267	19223948	-	+100k	+	AS	transcription factor AP-2 beta isoform 2
**501**	UID520	chr1	NM_009334	19109931	19120329	19194127	19223948	-	+100k	+	AS	transcription factor AP-2 beta isoform 1
**502**	UID520	chr1	NM_153154	19109931	19120329	19088235	19151546	-	-50k	+	AS	transcription factor AP-2, delta
**503**	UID522	chr1	NM_009352	15720779	15731177	15790871	15828643	-	+100k	+	AS	telomeric repeat binding factor 1
**504**	UID523	chr19	NM_026640	12566893	12577334	12640569	12656741	+	+100k	+	AS	hypothetical protein LOC107373
**505**	UID523	chr19	NM_146683	12566893	12577334	12489355	12490312	+	-100k	+	S	olfactory receptor 1441
**506**	UID523	chr19	NM_019540	12566893	12577334	12494949	12499155	+	-100k	+	S	pore forming protein-like
**507**	UID523	chr19	NM_010821	12566893	12577334	12527823	12532330	+	-50k	+	S	macrophage expressed gene 1
**508**	UID523	chr19	NM_172442	12566893	12577334	12533380	12569041	+	-50k	-	AS	deltex 4 homolog
**509**	UID524	chr19	NM_146345	13834768	13845209	13771847	13772807	+	-100k	+	S	olfactory receptor 1491
**510**	UID524	chr19	NM_146990	13834768	13845209	13816126	13817074	+	-20k	+	S	olfactory receptor 1494

**511**	UID524	chr19	NM_146344	13834768	13845209	13835362	13836322	+	+5k	+	AS	olfactory receptor 1495
**512**	UID524	chr19	NM_146989	13834768	13845209	13847632	13848586	+	+20k	+	AS	olfactory receptor 1496
**513**	UID524	chr19	NM_146741	13834768	13845209	13861683	13862628	+	+50k	-	AS	olfactory receptor 1497
**514**	UID524	chr19	NM_146796	13834768	13845209	13881662	13882607	+	+50k	-	AS	olfactory receptor 1499
**515**	UID524	chr19	NM_001011831	13834768	13845209	13894477	13895413	+	+100k	-	AS	olfactory receptor 1500
**516**	UID524	chr19	NM_146633	13834768	13845209	13905242	13906190	+	+100k	-	AS	olfactory receptor 1501
**517**	UID524	chr19	NM_146797	13834768	13845209	13928813	13929764	+	+100k	+	AS	olfactory receptor 1502
**518**	UID525	chr19	NM_146796	13963910	13974351	13881662	13882607	+	-100k	-	AS	olfactory receptor 1499
**519**	UID525	chr19	NM_001011831	13963910	13974351	13894477	13895413	+	-100k	-	AS	olfactory receptor 1500
**520**	UID525	chr19	NM_146633	13963910	13974351	13905242	13906190	+	-100k	-	AS	olfactory receptor 1501
**521**	UID525	chr19	NM_146797	13963910	13974351	13928813	13929764	+	-50k	+	S	olfactory receptor 1502
**522**	UID525	chr19	NM_146634	13963910	13974351	13954279	13955227	+	-10k	-	AS	olfactory receptor 1504
**523**	UID525	chr19	NM_001011850	13963910	13974351	13986040	13986991	+	+50k	+	AS	olfactory receptor 1505
**524**	UID526	chr19	NM_001011850	14064026	14074258	13986040	13986991	+	-100k	+	S	olfactory receptor 1505
**525**	UID529	chr19	NM_009551	21282478	21292918	21339374	21349386	+	+100k	+	AS	zinc finger, AN1-type domain 5
**526**	UID530	chr19	NM_009551	21301487	21311973	21339374	21349386	+	+50k	+	AS	zinc finger, AN1-type domain 5
**527**	UID534	chr19	NM_016861	40197212	40207647	40275549	40324927	+	+100k	-	AS	carboxyl terminal LIM domain protein 1
**528**	UID534	chr19	NM_145499	40197212	40207647	40206671	40240582	+	+10k	-	AS	cytochrome P450, family 2, subfamily c,
**529**	UID534	chr19	NM_134144	40197212	40207647	40143006	40167262	+	-100k	+	S	cytochrome P450, family 2, subfamily c,
**530**	UID539	chr19	NM_001033172	59867731	59878130	59958358	59998027	-	+100k	-	S	Rab11-family interacting protein 2 isoform 1
**531**	UID541	chr19	NM_025696	48278105	48288544	48259335	48858816	-	-20k	+	AS	sortilin-related VPS10 domain containing
**532**	UID543	chr19	NM_146097	24893139	24903537	24987523	25028655	-	+100k	-	S	COBW domain containing 1
**533**	UID543	chr19	NM_008022	24893139	24903537	24966061	24968406	-	+100k	-	S	forkhead box D4
**534**	UID544	chr19	NM_001035244	22710364	22720762	22759715	22993586	-	+50k	+	AS	transient receptor potential cation channel,
**535**	UID544	chr19	NM_001035246	22710364	22720762	22759715	22836477	-	+50k	+	AS	transient receptor potential cation channel,
**536**	UID544	chr19	NM_001035245	22710364	22720762	22759715	22839476	-	+50k	+	AS	transient receptor potential cation channel,
**537**	UID545	chr19	NM_028953	20815020	20825461	20850554	21021299	-	+50k	-	S	transmembrane channel-like gene family 1
**538**	UID545	chr19	NM_011921	20815020	20825461	20760049	20794653	-	-100k	-	AS	aldehyde dehydrogenase family 1, subfamily A7
**539**	UID547	chr19	NM_025731	7740731	7751130	7679613	7706283	-	-100k	+	AS	HRAS-like suppressor family, member 5
**540**	UID547	chr19	NM_019516	7740731	7751130	7663705	7674221	-	-100k	-	AS	lectin, galactose binding, soluble 12
**541**	UID547	chr19	NM_144785	7740731	7751130	7740120	7778298	-	-5k	-	AS	solute carrier family 22 (organic anion
**542**	UID549	chr13	NM_172445	8805838	8816222	8802213	8870288	+	-5k	-	AS	WD repeat domain 37 isoform a
**543**	UID549	chr13	NM_001039388	8805838	8816222	8802213	8870976	+	-5k	-	AS	WD repeat domain 37 isoform a
**544**	UID549	chr13	NM_001039389	8805838	8816222	8849724	8870288	+	+50k	-	AS	WD repeat domain 37 isoform b
**545**	UID549	chr13	NM_177960	8805838	8816222	8885219	8891614	+	+100k	+	AS	isopentenyl-diphosphate delta isomerase isoform
**546**	UID549	chr13	NM_145360	8805838	8816222	8885230	8891616	+	+100k	+	AS	isopentenyl-diphosphate delta isomerase isoform
**547**	UID550	chr13	NM_134244	22427901	22438342	22335375	22336272	+	-100k	+	S	vomeronasal 1 receptor, H21
**548**	UID550	chr13	NM_134220	22427901	22438342	22361810	22362713	+	-100k	+	S	vomeronasal 1 receptor, I3
**549**	UID550	chr13	NM_134213	22427901	22438342	22390002	22391106	+	-50k	+	S	vomeronasal 1 receptor, H4
**550**	UID550	chr13	NM_134212	22427901	22438342	22402493	22403432	+	-50k	+	S	vomeronasal 1 receptor, H3
**551**	UID550	chr13	NM_134221	22427901	22438342	22482082	22482985	+	+100k	+	AS	vomeronasal 1 receptor, I4
**552**	UID550	chr13	NM_134224	22427901	22438342	22508801	22509710	+	+100k	-	AS	vomeronasal 1 receptor, I7
**553**	UID551	chr13	NM_175663	24032958	24043399	23941237	23941621	+	-100k	-	AS	histone cluster 1, H2ba

**554**	UID551	chr13	NM_175658	24032958	24043399	23941926	23942316	+	-100k	+	S	histone cluster 1, H2aa
**555**	UID551	chr13	NM_145399	24032958	24043399	23960921	23998679	+	-100k	-	AS	secretagogin, EF-hand calcium binding protein
**556**	UID551	chr13	NM_026825	24032958	24043399	24019948	24288255	+	-20k	-	AS	leucine rich repeat containing 16A
**557**	UID556	chr13	NM_011455	33477008	33487449	33492254	33503463	+	+20k	+	AS	serine (or cysteine) proteinase inhibitor, clade
**558**	UID558	chr13	NM_001122948	40634228	40644626	40726648	40741417	+	+100k	-	AS	transcription factor AP-2, alpha isoform b
**559**	UID558	chr13	NM_011547	40634228	40644626	40726648	40744797	+	+100k	-	AS	transcription factor AP-2, alpha isoform a
**560**	UID559	chr13	NM_031166	48306418	48316859	48273363	48275973	+	-50k	+	S	inhibitor of DNA binding 4
**561**	UID561	chr13	NM_008957	63443664	63454105	63520754	63574742	+	+100k	-	AS	patched
**562**	UID565	chr13	NM_026173	97457507	97467896	97488992	97516286	-	+50k	+	AS	proteome of centriole 5
**563**	UID565	chr13	NM_001042714	97457507	97467896	97516832	97571859	-	+100k	-	S	ankyrin repeat and death domain containing 1B
**564**	UID571	chr13	NM_183197	33424882	33435323	33331541	33342831	-	-100k	+	AS	serine (or cysteine) proteinase inhibitor, clade
**565**	UID571	chr13	NM_011455	33424882	33435323	33492254	33503463	-	+100k	+	AS	serine (or cysteine) proteinase inhibitor, clade
**566**	UID572	chr13	NM_144536	29255505	29265949	29332770	29863053	-	+100k	-	S	CDK5 regulatory subunit associated protein
**567**	UID577	chr13	NM_134221	22549637	22560035	22482082	22482985	-	-100k	+	AS	vomeronasal 1 receptor, I4
**568**	UID577	chr13	NM_134224	22549637	22560035	22508801	22509710	-	-50k	-	AS	vomeronasal 1 receptor, I7
**569**	UID577	chr13	NM_134236	22549637	22560035	22531515	22532451	-	-20k	+	AS	vomeronasal 1 receptor, H11
**570**	UID577	chr13	NM_134217	22549637	22560035	22599444	22600395	-	+50k	-	S	vomeronasal 1 receptor, H8
**571**	UID577	chr13	NM_134216	22549637	22560035	22627561	22628500	-	+100k	-	S	vomeronasal 1 receptor, H7
**572**	UID577	chr13	NM_001045544	22549637	22560035	22563665	22564574	-	+20k	+	AS	vomeronasal receptor-like
**573**	UID578	chr13	NM_145846	22211378	22221864	22151985	22152990	-	-100k	-	AS	vomeronasal 1 receptor, I8
**574**	UID578	chr13	NM_145845	22211378	22221864	22194610	22195513	-	-20k	-	AS	vomeronasal 1 receptor, I1
**575**	UID578	chr13	NM_134225	22211378	22221864	22226325	22227285	-	+20k	-	S	vomeronasal 1 receptor, I9
**576**	UID578	chr13	NM_134223	22211378	22221864	22285826	22286777	-	+100k	+	AS	vomeronasal 1 receptor, I6
**577**	UID578	chr13	NM_001080972	22211378	22221864	22251679	22252570	-	+50k	+	AS	vomeronasal 1 receptor-like
**578**	UID583	chrX	NM_001085537	8543202	8553688	8507557	8508096	+	-50k	+	S	novel protein similar to histone H2a
**579**	UID583	chrX	NM_007807	8543202	8553688	8592210	8626283	+	+50k	-	AS	cytochrome b-245, beta polypeptide
**580**	UID584	chrX	NM_019634	9678816	9689257	9642074	9753563	+	-50k	+	S	tetraspanin 7
**581**	UID586	chrX	NM_001034100	13345061	13355544	13368106	13368616	+	+50k	+	AS	hypothetical protein LOC385328
**582**	UID594	chrX	NM_001081666	23128866	23139264	23114660	23115893	+	-20k	-	AS	hypothetical protein LOC245376
**583**	UID594	chrX	NM_001034864	23128866	23139264	23049721	23064509	+	-100k	+	S	Tes-like protein
**584**	UID595	chrX	NM_001100446	23675106	23685547	23709208	23731854	+	+50k	-	AS	hypothetical protein LOC100043216
**585**	UID595	chrX	NM_001100444	23675106	23685547	23631266	23657719	+	-50k	-	AS	hypothetical protein LOC546272
**586**	UID598	chrX	NM_001009947	32251489	32261930	32320276	32508007	+	+100k	+	AS	dedicator of cytokinesis 11
**587**	UID599	chrX	NM_001009947	32289440	32300838	32320276	32508007	+	+50k	+	AS	dedicator of cytokinesis 11
**588**	UID600	chrX	NM_023894	34151561	34162002	34143723	34146397	+	-10k	-	AS	reproductive homeobox on X chromosome, 9
**589**	UID600	chrX	NM_001025086	34151561	34162002	34076312	34085798	+	-100k	+	S	reproductive homeobox 7
**590**	UID600	chrX	NM_008955	34151561	34162002	34071681	34074484	+	-100k	+	S	reproductive homeobox on X chromosome, 6
**591**	UID600	chrX	NM_001004193	34151561	34162002	34119402	34123571	+	-50k	-	AS	reproductive homeobox on X chromosome, 8
**592**	UID605	chrX	NM_001033422	37985916	37996356	38039620	38156528	+	+100k	-	AS	THO complex subunit 2
**593**	UID606	chrX	NM_011855	38874332	38884773	38777019	39519413	+	-100k	-	AS	odd Oz/ten-m homolog 1
**594**	UID610	chrX	NM_175539	40628796	40639194	40610083	40612964	+	-20k	-	AS	WD repeat domain 40C
**595**	UID612	chrX	NM_028514	42514326	42524724	42573633	42574972	+	+100k	+	AS	actin-related protein T1
**596**	UID615	chrX	NM_053123	44031125	44041559	44053996	44137146	+	+50k	-	AS	SWI/SNF related, matrix associated, actin

**597**	UID616	chrX	NM_053123	44108861	44119302	44053996	44137146	+	-100k	-	AS	SWI/SNF related, matrix associated, actin
**598**	UID616	chrX	NM_177215	44108861	44119302	44157082	44210493	+	+50k	+	AS	phosphatidylinositol polyphosphate
**599**	UID619	chrX	NM_146390	46325619	46336102	46253932	46254862	+	-100k	-	AS	olfactory receptor 1323
**600**	UID622	chrX	NM_015819	47678305	47688703	47631838	47925104	+	-50k	-	AS	heparan sulfate 6-O-sulfotransferase 2 isoform
**601**	UID622	chrX	NM_001077202	47678305	47688703	47631838	47926229	+	-50k	-	AS	heparan sulfate 6-O-sulfotransferase 2 isoform
**602**	UID623	chrX	NM_031388	47970946	47983679	47998585	48042380	+	+50k	-	AS	ubiquitin specific peptidase 26
**603**	UID624	chrX	NM_027510	52425594	52435990	52483355	52500280	+	+100k	+	AS	Xlr-like
**604**	UID625	chrX	NM_001033360	53887439	53897837	53843451	53850501	+	-50k	-	AS	G protein-coupled receptor 101
**605**	UID633	chrX	NM_027512	60383449	60393890	60434226	60439443	+	+100k	+	AS	hypothetical protein LOC70696
**606**	UID637	chrX	NM_001161431	62866501	62876899	62909982	62922066	+	+50k	+	AS	SLIT and NTRK-like family, member 2 precursor
**607**	UID637	chrX	NM_198863	62866501	62876899	62913823	62922066	+	+50k	+	AS	SLIT and NTRK-like family, member 2 precursor
**608**	UID641	chrX	NM_010498	66522155	66532553	66603734	66625640	+	+100k	-	AS	iduronate-2-sulfatase isoform a
**609**	UID641	chrX	NM_001038991	66522155	66532553	66603734	66625640	+	+100k	-	AS	iduronate-2-sulfatase isoform b
**610**	UID641	chrX	NM_001038990	66522155	66532553	66603734	66625640	+	+100k	-	AS	iduronate-2-sulfatase isoform b
**611**	UID642	chrX	NM_020280	68393346	68403784	68474736	68475683	+	+100k	+	AS	melanoma antigen family A, 4
**612**	UID644	chrX	NM_001081670	71115017	71125500	71089915	71096075	+	-50k	+	S	hypothetical protein LOC546325
**613**	UID645	chrX	NM_181584	71182549	71192991	71241269	71337625	+	+100k	-	AS	growth factor receptor bound protein
**614**	UID645	chrX	NM_001081670	71182549	71192991	71089915	71096075	+	-100k	+	S	hypothetical protein LOC546325
**615**	UID646	chrX	NM_026126	71553429	71563870	71635141	71648836	+	+100k	+	AS	FUN14 domain containing 2
**616**	UID647	chrX	NM_001013760	72486155	72496601	72517024	72520392	+	+50k	+	AS	hypothetical protein LOC238829
**617**	UID648	chrX	NM_001013760	72612631	72623069	72517024	72520392	+	-100k	+	S	hypothetical protein LOC238829
**618**	UID648	chrX	NM_001033799	72612631	72623069	72646068	72650698	+	+50k	+	AS	hypothetical protein LOC619294
**619**	UID658	chrX	NM_173435	77662868	77673266	77672293	77710841	+	+10k	-	AS	hypothetical protein LOC245492
**620**	UID661	chrX	NM_001160403	83071903	83082300	82999961	84368342	+	-100k	-	AS	interleukin 1 receptor accessory protein-like 1
**621**	UID663	chrX	NM_008821	85671989	85682387	85656566	85662408	+	-20k	-	AS	plasmacytoma expressed transcript 2
**622**	UID664	chrX	NM_008821	85738006	85748447	85656566	85662408	+	-100k	-	AS	plasmacytoma expressed transcript 2
**623**	UID668	chrX	NM_029590	91943877	91954275	91935308	91935980	+	-10k	-	AS	hypothetical protein LOC76386
**624**	UID668	chrX	NM_001034907	91943877	91954275	91914396	92130689	+	-50k	+	S	zinc finger CCCH-type containing 12B
**625**	UID670	chrX	NM_001159627	92578862	92589303	92659064	92777201	+	+100k	+	AS	hephaestin isoform 1
**626**	UID670	chrX	NM_010417	92578862	92589303	92658154	92777201	+	+100k	+	AS	hephaestin isoform 1
**627**	UID670	chrX	NM_172931	92578862	92589303	92509585	92559959	+	-100k	-	AS	hypothetical protein LOC245525
**628**	UID670	chrX	NM_181273	92578862	92589303	92659064	92734142	+	+100k	+	AS	hephaestin isoform 2
**629**	UID670	chrX	NM_001159628	92578862	92589303	92658154	92777201	+	+100k	+	AS	hephaestin isoform 3
**630**	UID676	chrX	NM_173021	98760622	98771020	98716693	98846965	+	-50k	-	AS	phosphorylase kinase alpha 1 isoform 2
**631**	UID676	chrX	NM_008832	98760622	98771020	98716693	98846965	+	-50k	-	AS	phosphorylase kinase alpha 1 isoform 1
**632**	UID677	chrX	NM_001077354	100367420	100377861	100280154	100403836	+	-100k	-	AS	hypothetical protein LOC245555
**633**	UID678	chrX	NM_175358	100822324	100832765	100741268	100873522	+	-100k	-	AS	zinc finger, DHHC domain containing 15
**634**	UID679	chrX	NM_175358	100838663	100849097	100741268	100873522	+	-100k	-	AS	zinc finger, DHHC domain containing 15
**635**	UID680	chrX	NM_053206	100997922	101008363	101057670	101059986	+	+100k	-	AS	melanoma antigen, family E, 2
**636**	UID682	chrX	NM_026312	101219972	101230463	101282493	101287905	+	+100k	+	AS	hypothetical protein LOC67683
**637**	UID683	chrX	NM_009530	101936168	101946609	102000335	102132091	+	+100k	-	AS	alpha thalassemia/mental retardation syndrome
**638**	UID683	chrX	NM_030614	101936168	101946609	101967195	101979251	+	+50k	+	AS	fibroblast growth factor 16
**639**	UID684	chrX	NM_009530	101939900	101950299	102000335	102132091	+	+100k	-	AS	alpha thalassemia/mental retardation syndrome

**640**	UID684	chrX	NM_030614	101939900	101950299	101967195	101979251	+	+50k	+	AS	fibroblast growth factor 16
**641**	UID685	chrX	NM_001033251	103529933	103540374	103458746	103498026	+	-100k	+	S	G protein-coupled receptor 174
**642**	UID685	chrX	NM_008409	103529933	103540374	103599913	103606079	+	+100k	-	AS	integral membrane protein 2A
**643**	UID689	chrX	NM_033605	109465788	109477995	109415199	109953330	+	-100k	+	S	dachshund 2 isoform 2
**644**	UID689	chrX	NM_001142570	109465788	109477995	109415199	109952035	+	-100k	+	S	dachshund 2 isoform 1
**645**	UID691	chrX	NM_001081661	111115236	111125677	111024525	111025454	+	-100k	+	S	hypothetical protein LOC75097
**646**	UID691	chrX	NR_003645	111115236	111125677	111021955	111022885	+	-100k	+	S	
**647**	UID692	chrX	NM_001102665	113203371	113213854	113131700	113132048	+	-100k	+	S	predicted gene 14920
**648**	UID694	chrX	NM_001081385	116499515	116509953	116407270	117023394	+	-100k	+	S	protocadherin 11 X-linked
**649**	UID700	chrX	NM_001111144	122286109	122296504	122373175	122375212	+	+100k	-	AS	hypothetical protein LOC214308
**650**	UID700	chrX	NM_177705	122286109	122296504	122280056	122283460	+	-10k	-	AS	Myb-like DNA-binding domain containing protein
**651**	UID703	chrX	NM_031259	130235289	130245688	130290894	130311118	+	+100k	-	AS	nuclear RNA export factor 2
**652**	UID703	chrX	NM_175446	130235289	130245688	130317736	130355573	+	+100k	-	AS	zinc finger, matrin type 1
**653**	UID703	chrX	NM_026139	130235289	130245688	130150510	130155358	+	-100k	-	AS	armadillo repeat containing, X-linked 2
**654**	UID704	chrX	NM_031390	130737689	130748979	130648675	130659000	+	-100k	+	S	preferentially expressed antigen in
**655**	UID704	chrX	NM_183320	130737689	130748979	130719647	130729757	+	-20k	+	S	hypothetical protein LOC331529
**656**	UID704	chrX	NM_183320	130737689	130748979	130780198	130790361	+	+50k	+	AS	hypothetical protein LOC331529
**657**	UID704	chrX	NM_177918	130798287	130809578	130840951	130850958	+	+50k	+	AS	hypothetical protein LOC331531
**658**	UID704	chrX	NM_001099321	130798287	130809578	130840951	130850958	+	+50k	+	AS	hypothetical protein LOC100040635
**659**	UID704	chrX	NM_183320	130798287	130809578	130719647	130729757	+	-100k	+	S	hypothetical protein LOC331529
**660**	UID704	chrX	NM_183320	130798287	130809578	130780198	130790361	+	-20k	+	S	hypothetical protein LOC331529
**661**	UID705	chrX	NM_177918	130798288	130809578	130840951	130850958	+	+50k	+	AS	hypothetical protein LOC331531
**662**	UID705	chrX	NM_001099321	130798288	130809578	130840951	130850958	+	+50k	+	AS	hypothetical protein LOC100040635
**663**	UID705	chrX	NM_183320	130798288	130809578	130719647	130729757	+	-100k	+	S	hypothetical protein LOC331529
**664**	UID705	chrX	NM_183320	130798288	130809578	130780198	130790361	+	-20k	+	S	hypothetical protein LOC331529
**665**	UID705	chrX	NM_031390	130737690	130748979	130648675	130659000	+	-100k	+	S	preferentially expressed antigen in
**666**	UID705	chrX	NM_183320	130737690	130748979	130719647	130729757	+	-20k	+	S	hypothetical protein LOC331529
**667**	UID705	chrX	NM_183320	130737690	130748979	130780198	130790361	+	+50k	+	AS	hypothetical protein LOC331529
**668**	UID707	chrX	NM_011635	134726288	134736729	134680046	134684529	+	-50k	+	S	tumor rejection antigen P1A
**669**	UID708	chrX	NM_019496	138106027	138116468	138099841	138213096	+	-10k	-	AS	AMMECR1 protein
**670**	UID711	chrX	NM_008824	145867580	145878021	145930636	145984576	+	+100k	+	AS	6-phosphofructo-2-kinase/fructose-2,
**671**	UID711	chrX	NM_029943	145867580	145878021	145912222	145928865	+	+50k	-	AS	apurinic/apyrimidinic endonuclease 2
**672**	UID711	chrX	NM_001102446	145867580	145878021	145888132	145911338	+	+50k	+	AS	5-aminolevulinate synthase 2 isoform b
**673**	UID711	chrX	NM_009653	145867580	145878021	145888132	145911338	+	+50k	+	AS	5-aminolevulinate synthase 2 isoform a
**674**	UID713	chrX	NM_177201	146839563	146849961	146861387	146966549	+	+50k	+	AS	PHD finger protein 8 isoform a
**675**	UID713	chrX	NM_001113354	146839563	146849961	146861387	146974573	+	+50k	+	AS	PHD finger protein 8 isoform b
**676**	UID714	chrX	NM_021523	147081291	147091734	147143997	147276133	+	+100k	+	AS	HECT, UBA and WWE domain containing 1
**677**	UID715	chrX	NM_027543	147777164	147787562	147685361	147708827	+	-100k	-	AS	G-protein coupled receptor 173
**678**	UID715	chrX	NM_029836	147777164	147787562	147677567	147683200	+	-100k	-	AS	nucleolar TGF-beta1 target protein
**679**	UID716	chrX	NM_172441	147861759	147872200	147950224	148110177	+	+100k	-	AS	shroom family member 2
**680**	UID717	chrX	NM_029199	149838496	149848729	149822717	149825398	+	-20k	+	S	hypothetical protein LOC75185
**681**	UID717	chrX	NM_001037167	149838496	149848729	149915943	149920046	+	+100k	-	AS	hypothetical protein LOC434881
**682**	UID726	chrX	NM_009031	158010254	158020652	158104476	158123195	+	+100k	+	AS	retinoblastoma binding protein 7

**683**	UID728	chrX	NM_183427	160429873	160440314	160473121	160671086	+	+50k	-	AS	glycine receptor, alpha 2 subunit precursor
**684**	UID729	chrX	NM_133211	162554890	162565331	162649035	162674633	+	+100k	-	AS	toll-like receptor 7 precursor
**685**	UID729	chrX	NM_021278	162554890	162565331	162551198	162553323	+	-5k	-	AS	thymosin, beta 4, X chromosome
**686**	UID729	chrX	NM_133212	162554890	162565331	162586836	162607893	+	+50k	-	AS	toll-like receptor 8 precursor
**687**	UID730	chrX	NM_001081978	164501103	164511543	164520218	164531314	+	+20k	-	AS	amelogenin X chromosome isoform 1
**688**	UID730	chrX	NM_009666	164501103	164511543	164524125	164531314	+	+50k	-	AS	amelogenin X chromosome isoform 2
**689**	UID731	chrX	NM_008222	164626521	164638971	164655634	164664448	+	+50k	-	AS	holocytochrome c synthase
**690**	UID732	chrX	NM_008222	164752956	164763391	164655634	164664448	+	-100k	-	AS	holocytochrome c synthase
**691**	UID733	chrX	NM_010797	165081605	165092046	165029351	165334902	+	-100k	+	S	midline 1
**692**	UID733	chrX	NR_003635	165081605	165092046	165120482	165123740	+	+50k	-	AS	
**693**	UID734	chrX	NM_027027	159905773	159916214	159842007	159883857	-	-100k	+	AS	ankyrin repeat and SOCS box-containing 9
**694**	UID735	chrX	NM_010216	159694299	159704740	159717652	159746752	-	+50k	+	AS	c-fos induced growth factor precursor
**695**	UID735	chrX	NM_026853	159694299	159704740	159782205	159803205	-	+100k	+	AS	ankyrin repeat and SOCS box-containing 11
**696**	UID735	chrX	NM_011081	159694299	159704740	159763891	159778020	-	+100k	+	AS	phosphatidylinositol glycan, class A
**697**	UID735	chrX	NM_027153	159694299	159704740	159613541	159717010	-	-100k	+	AS	pirin
**698**	UID736	chrX	NM_198409	157049270	157059668	157061657	157123599	-	+20k	+	AS	retinoic acid induced 2
**699**	UID736	chrX	NM_001103367	157049270	157059668	157061140	157123599	-	+20k	+	AS	retinoic acid induced 2
**700**	UID739	chrX	NM_011147	155996464	156006905	155967199	156064077	-	-50k	-	AS	protein phosphatase with EF hand calcium-binding
**701**	UID740	chrX	NM_172783	155877732	155888131	155846794	155935827	-	-50k	+	AS	phosphorylase kinase alpha 2
**702**	UID740	chrX	NM_011147	155877732	155888131	155967199	156064077	-	+100k	-	S	protein phosphatase with EF hand calcium-binding
**703**	UID742	chrX	NM_001081667	153106281	153116722	153162539	153164474	-	+100k	+	AS	kelch-like 34
**704**	UID742	chrX	NM_177751	153106281	153116722	153165677	153387215	-	+100k	-	S	connector enhancer of kinase suppressor of Ras
**705**	UID742	chrX	NM_025357	153106281	153116722	153043222	153096694	-	-100k	+	AS	small muscle protein, X-linked
**706**	UID745	chrX	NM_001081671	150561023	150571464	150465696	150488565	-	-100k	-	AS	hypothetical protein LOC73347
**707**	UID745	chrX	NM_019736	150561023	150571464	150603215	150638223	-	+50k	+	AS	acyl-CoA thioesterase 9
**708**	UID745	chrX	NM_009121	150561023	150571464	150553854	150557125	-	-10k	-	AS	diamine N-acetyltransferase 1
**709**	UID746	chrX	NM_030700	146201329	146211689	146147136	146154998	-	-100k	-	AS	melanoma antigen family D, 2
**710**	UID746	chrX	NR_003597	146201329	146211689	146168404	146197658	-	-50k	+	AS	
**711**	UID747	chrX	NM_019548	145954100	145964498	145986018	145998152	-	+50k	-	S	trophinin isoform 3
**712**	UID747	chrX	NM_008824	145954100	145964498	145930636	145984576	-	-50k	+	AS	6-phosphofructo-2-kinase/fructose-2,
**713**	UID747	chrX	NM_029943	145954100	145964498	145912222	145928865	-	-50k	-	AS	apurinic/apyrimidinic endonuclease 2
**714**	UID747	chrX	NM_001102446	145954100	145964498	145888132	145911338	-	-100k	+	AS	5-aminolevulinate synthase 2 isoform b
**715**	UID747	chrX	NM_009653	145954100	145964498	145888132	145911338	-	-100k	+	AS	5-aminolevulinate synthase 2 isoform a
**716**	UID747	chrX	NM_001002272	145954100	145964498	145986018	145998152	-	+50k	-	S	trophinin isoform 1
**717**	UID748	chrX	NM_178358	140479341	140489741	140536726	140595262	-	+100k	-	S	lipoma HMGIC fusion partner-like 1 precursor
**718**	UID749	chrX	NM_026247	139467596	139478038	139564390	139571556	-	+100k	+	AS	glycosyltransferase 28 domain containing 1
**719**	UID750	chrX	NM_031258	138501851	138512294	138543525	138640630	-	+50k	-	S	chordin-like 1 2
**720**	UID750	chrX	NM_001114385	138501851	138512294	138532041	138640630	-	+50k	-	S	chordin-like 1 1
**721**	UID751	chrX	NM_177592	137954205	137964646	137927785	138084606	-	-50k	+	AS	transmembrane protein 164
**722**	UID753	chrX	NM_010572	136986733	136997174	136957365	136971585	-	-50k	-	AS	insulin receptor substrate 4
**723**	UID754	chrX	NM_001163155	136806493	136816936	136721786	136935603	-	-100k	+	AS	collagen, type IV, alpha 5
**724**	UID754	chrX	NM_007736	136806493	136816936	136721786	136935603	-	-100k	+	AS	collagen, type IV, alpha 5
**725**	UID755	chrX	NM_013757	129220484	129230882	129282734	129328133	-	+100k	-	S	synaptotagmin-like 4

**726**	UID755	chrX	NM_001083895	129220484	129230882	129254775	129278800	-	+50k	+	AS	sushi-repeat containing protein precursor
**727**	UID755	chrX	NM_026838	129220484	129230882	129254775	129278800	-	+50k	+	AS	sushi-repeat containing protein precursor
**728**	UID755	chrX	NM_019656	129220484	129230882	129237419	129244779	-	+20k	-	S	tetraspanin 6
**729**	UID755	chrX	NM_022322	129220484	129230882	129197357	129211927	-	-50k	+	AS	tenomodulin
**730**	UID765	chrX	NM_001033241	122591316	122601757	122507566	122531581	-	-100k	+	AS	hypothetical protein LOC211208
**731**	UID774	chrX	NM_031382	108260980	108271463	108210463	108244270	-	-100k	+	AS	testis expressed gene 16
**732**	UID779	chrX	NM_145224	103932616	103943014	103870682	103891694	-	-100k	+	AS	T-box 22 isoform 1
**733**	UID779	chrX	NM_025921	103932616	103943014	103985472	104019053	-	+100k	-	S	hypothetical protein LOC67028
**734**	UID779	chrX	NM_181319	103932616	103943014	103881681	103891696	-	-100k	+	AS	T-box 22 isoform 2
**735**	UID779	chrX	NM_001163104	103932616	103943014	104019290	104075628	-	+100k	+	AS	hypothetical protein LOC213449
**736**	UID780	chrX	NM_001122595	103252805	103263246	103352172	103376380	-	+100k	+	AS	purinergic receptor P2Y G-protein coupled
**737**	UID780	chrX	NM_001122596	103252805	103263246	103351645	103376380	-	+100k	+	AS	purinergic receptor P2Y G-protein coupled
**738**	UID780	chrX	NM_172435	103252805	103263246	103292053	103307689	-	+50k	+	AS	purinergic receptor P2Y, G-protein coupled 10
**739**	UID780	chrX	NM_175442	103252805	103263246	103352333	103376380	-	+100k	+	AS	purinergic receptor P2Y G-protein coupled
**740**	UID781	chrX	NM_172435	103249186	103259584	103292053	103307689	-	+50k	+	AS	purinergic receptor P2Y, G-protein coupled 10
**741**	UID782	chrX	NM_009592	100468286	100478684	100483283	100616565	-	+20k	-	S	ATP-binding cassette, sub-family B (MDR/TAP),
**742**	UID783	chrX	NM_031384	97060755	97071196	97041382	97262356	-	-20k	-	AS	testis expressed gene 11
**743**	UID783	chrX	NM_016747	97060755	97071196	96970465	97021127	-	-100k	+	AS	synapse-associated protein 102
**744**	UID784	chrX	NM_031384	96960751	96971195	97041382	97262356	-	+100k	-	S	testis expressed gene 11
**745**	UID784	chrX	NM_023608	96960751	96971195	96932565	96941618	-	-50k	+	AS	osteoblast differentiation promoting factor
**746**	UID784	chrX	NM_016747	96960751	96971195	96970465	97021127	-	+10k	+	AS	synapse-associated protein 102
**747**	UID789	chrX	NM_001003916	91895002	91905443	91841915	91861151	-	-100k	-	AS	zinc finger, C4H2 domain containing
**748**	UID789	chrX	NM_029590	91895002	91905443	91935308	91935980	-	+50k	-	S	hypothetical protein LOC76386
**749**	UID789	chrX	NM_001034907	91895002	91905443	91914396	92130689	-	+20k	+	AS	zinc finger CCCH-type containing 12B
**750**	UID790	chrX	NM_080858	91614141	91624539	91672917	91680848	-	+100k	-	S	ankyrin repeat and SOCS box-containing 12
**751**	UID790	chrX	NM_175179	91614141	91624539	91623032	91647559	-	+10k	-	S	RIKEN cDNA 2810002O09
**752**	UID793	chrX	NM_001101450	87090743	87101184	87144860	87145851	-	+100k	+	AS	hypothetical protein LOC212952
**753**	UID800	chrX	NM_001033492	82469542	82479941	82502974	82508938	-	+50k	-	S	hypothetical protein LOC434903
**754**	UID800	chrX	NM_007430	82469542	82479941	82444493	82448663	-	-50k	+	AS	nuclear receptor subfamily 0, group B, member 1
**755**	UID807	chrX	NM_001113734	75963409	75973850	75875949	75888301	-	-100k	-	AS	melanoma antigen family B, 16
**756**	UID807	chrX	NM_028025	75963409	75973850	75875949	75924154	-	-100k	-	AS	melanoma antigen family B, 16
**757**	UID809	chrX	NM_008621	71446004	71456445	71362452	71383669	-	-100k	-	AS	membrane protein, palmitoylated
**758**	UID809	chrX	NM_001030307	71446004	71456445	71348635	71362490	-	-100k	+	AS	dyskerin
**759**	UID809	chrX	NM_001161373	71446004	71456445	71425438	71632761	-	-50k	-	AS	coagulation factor VIII isoform 2
**760**	UID809	chrX	NM_001161374	71446004	71456445	71425438	71632761	-	-50k	-	AS	coagulation factor VIII isoform 3
**761**	UID809	chrX	NM_007977	71446004	71456445	71425438	71632761	-	-50k	-	AS	coagulation factor VIII isoform 1
**762**	UID809	chrX	NM_001033786	71446004	71456445	71398776	71416471	-	-50k	-	AS	hypothetical protein LOC434800 precursor
**763**	UID810	chrX	NM_001099302	70867532	70877973	70891838	70898355	-	+50k	-	S	hypothetical protein LOC434797
**764**	UID810	chrX	NM_146398	70867532	70877973	70847047	70847995	-	-50k	+	AS	olfactory receptor 1325
**765**	UID811	chrX	NM_021365	69551720	69562203	69467084	69475173	-	-100k	+	AS	X-linked lymphocyte-regulated 4B
**766**	UID811	chrX	NM_007978	69551720	69562203	69481025	69483515	-	-100k	+	AS	factor 8-associated gene A
**767**	UID811	chrX	NM_183094	69551720	69562203	69486795	69495850	-	-100k	-	AS	X-linked lymphocyte-regulated 4C
**768**	UID811	chrX	NM_011727	69551720	69562203	69507259	69518110	-	-50k	-	AS	X-linked lymphocyte-regulated 3C

**769**	UID811	chrX	NM_031493	69551720	69562203	69537916	69543235	-	-20k	-	AS	X-linked lymphocyte-regulated 5C
**770**	UID811	chrX	NM_031494	69551720	69562203	69595339	69611799	-	+50k	+	AS	Zinc finger protein 275 isoform 1
**771**	UID811	chrX	NM_001160229	69551720	69562203	69595340	69611799	-	+50k	+	AS	Zinc finger protein 275 isoform 2
**772**	UID812	chrX	NM_009549	69183886	69194269	69240058	69284263	-	+100k	+	AS	zinc finger protein 185 isoform a
**773**	UID812	chrX	NM_001109043	69183886	69194269	69240058	69284263	-	+100k	+	AS	zinc finger protein 185 isoform b
**774**	UID812	chrX	NM_019405	69183886	69194269	69166284	69171064	-	-20k	-	AS	centrin 2
**775**	UID812	chrX	NM_010941	69183886	69194269	69171240	69211248	-	-20k	+	AS	NAD(P) dependent steroid dehydrogenase-like
**776**	UID812	chrX	NM_181855	69183886	69194269	69122086	69122818	-	-100k	+	AS	melanoma antigen family A, 9
**777**	UID816	chrX	NM_007724	68331045	68341443	68244594	68262938	-	-100k	+	AS	cyclic nucleotide gated channel alpha 2
**778**	UID837	chrX	NM_028375	49852137	49862578	49802548	49803761	-	-50k	+	AS	CAAX box 1 homolog C
**779**	UID837	chrX	NM_001018063	49852137	49862578	49863803	49865035	-	+20k	-	S	CAAX box 1 homolog B
**780**	UID837	chrX	NM_029142	49852137	49862578	49919457	49932849	-	+100k	+	AS	hypothetical protein LOC75013
**781**	UID837	chrX	NM_024170	49852137	49862578	49837117	49838390	-	-20k	-	AS	mammalian retrotransposon derived 8b
**782**	UID839	chrX	NM_207240	45851717	45862158	45928130	45929090	-	+100k	+	AS	olfactory receptor 1320
**783**	UID840	chrX	NM_001081123	45804763	45815204	45715076	45744877	-	-100k	+	AS	hypothetical protein LOC75404
**784**	UID841	chrX	NM_181751	44999254	45009694	44912605	44919105	-	-100k	-	AS	G-protein coupled receptor 119
**785**	UID841	chrX	NM_173376	44999254	45009694	44939630	44955346	-	-100k	+	AS	RNA binding motif protein, X-linked 2
**786**	UID841	chrX	NM_001085499	44999254	45009694	45086092	45122340	-	+100k	-	S	hypothetical protein LOC209005
**787**	UID842	chrX	NM_181751	44942595	44953036	44912605	44919105	-	-50k	-	AS	G-protein coupled receptor 119
**788**	UID842	chrX	NM_011398	44942595	44953036	44868204	44906921	-	-100k	+	AS	solute carrier family 25 (mitochondrial carrier)
**789**	UID842	chrX	NM_173376	44942595	44953036	44939630	44955346	-	-5k	+	AS	RNA binding motif protein, X-linked 2
**790**	UID846	chrX	NM_011364	38810394	38820835	38747234	38766702	-	-100k	+	AS	SH2 domain protein 1A
**791**	UID846	chrX	NM_011855	38810394	38820835	38777019	39519413	-	-50k	-	AS	odd Oz/ten-m homolog 1
**792**	UID847	chrX	NM_009688	38232653	38243094	38312462	38354291	-	+100k	+	AS	baculoviral IAP repeat-containing 4
**793**	UID848	chrX	NM_001033422	38014257	38024697	38039620	38156528	-	+50k	-	S	THO complex subunit 2
**794**	UID849	chrX	NM_016886	37548703	37559144	37645930	37923228	-	+100k	+	AS	glutamate receptor, ionotropic, AMPA3 precursor
**795**	UID854	chrX	NM_029707	21668758	21679155	21618590	21619357	-	-100k	+	AS	hypothetical protein LOC76705
**796**	UID855	chrX	NM_029707	21656263	21666661	21618590	21619357	-	-50k	+	AS	hypothetical protein LOC76705
**797**	UID856	chrX	NM_007429	20559183	20569627	20641584	20645790	-	+100k	+	AS	angiotensin II receptor, type 2
**798**	UID856	chrX	NM_198633	20559183	20569627	20517823	20521581	-	-50k	+	AS	hypothetical protein LOC331392
**799**	UID861	chrX	NM_175228	17517318	17527801	17575385	17618233	-	+100k	-	S	hypothetical protein LOC75905 precursor
**800**	UID864	chrX	NM_019634	9559001	9569487	9642074	9753563	-	+100k	+	AS	tetraspanin 7
**801**	UID865	chrX	NM_001085517	8659076	8669512	8728918	8729350	-	+100k	-	S	hypothetical protein LOC333452
**802**	UID865	chrX	NM_007807	8659076	8669512	8592210	8626283	-	-100k	-	AS	cytochrome b-245, beta polypeptide
**803**	UID866	chrX	NM_001085537	8583236	8593634	8507557	8508096	-	-100k	+	AS	novel protein similar to histone H2a
**804**	UID866	chrX	NM_007807	8583236	8593634	8592210	8626283	-	+10k	-	S	cytochrome b-245, beta polypeptide
**805**	UID869	chr4	NM_145711	6573715	6584156	6614532	6917870	+	+50k	-	AS	thymocyte selection-associated high mobility
**806**	UID870	chr4	NM_007592	8115690	8126131	8068639	8166188	+	-50k	-	AS	carbonic anhydrase-like
**807**	UID871	chr4	NM_133723	9510417	9520867	9548056	9570864	+	+50k	-	AS	aspartate beta-hydroxylase isoform 2
**808**	UID872	chr4	NM_026558	12137097	12147494	12080868	12099162	+	-100k	-	AS	hypothetical protein LOC68099
**809**	UID872	chr4	NM_028226	12137097	12147494	12067263	12073892	+	-100k	+	S	RNA binding motif protein 12B
**810**	UID875	chr4	NM_178617	14978824	14989265	14879392	15076278	+	-100k	-	AS	N-terminal EF-hand calcium binding protein 1
**811**	UID876	chr4	NM_013752	15982683	15993124	15885113	15919736	+	-100k	+	S	nibrin

**812**	UID876	chr4	NM_138952	15982683	15993124	16050521	16090645	+	+100k	-	AS	receptor-interacting serine-threonine kinase 2
**813**	UID876	chr4	NM_145950	15982683	15993124	15924267	15941024	+	-100k	-	AS	oxidative stress induced growth inhibitor family
**814**	UID885	chr4	NM_013927	19114350	19124791	19207996	19437770	+	+100k	+	AS	cyclic nucleotide gated channel beta 3
**815**	UID888	chr4	NM_001080771	21738950	21749391	21768314	21776798	+	+50k	-	AS	PR domain containing 13
**816**	UID888	chr4	NM_016746	21738950	21749391	21818537	21841381	+	+100k	+	AS	cyclin C isoform 1
**817**	UID888	chr4	NM_001122982	21738950	21749391	21818537	21838797	+	+100k	+	AS	cyclin C isoform 2
**818**	UID894	chr4	NM_025545	40783085	40793526	40870748	40891587	+	+100k	-	AS	aprataxin isoform a
**819**	UID894	chr4	NM_001025444	40783085	40793526	40870748	40891858	+	+100k	-	AS	aprataxin isoform b
**820**	UID895	chr4	NM_023231	43141302	43151743	43048789	43052484	+	-100k	-	AS	stomatin-like protein 2
**821**	UID895	chr4	NM_172691	43141302	43151743	43053609	43067303	+	-100k	-	AS	hypothetical protein LOC230088
**822**	UID895	chr4	NM_001081413	43141302	43151743	43080083	43285973	+	-100k	+	S	unc-13 homolog B
**823**	UID897	chr4	NM_145368	49347483	49357881	49400944	49429251	+	+100k	-	AS	acyl-coenzyme A amino acid N-acyltransferase 2
**824**	UID898	chr4	NM_001033351	49750172	49760570	49686325	49866181	+	-100k	-	AS	glutamate receptor ionotropic, NMDA3A
**825**	UID898	chr4	NM_001004025	49750172	49760570	49699846	49703083	+	-100k	-	AS	protein phosphatase 3, regulatory subunit B,
**826**	UID898	chr4	NM_001163263	49750172	49760570	49653159	49677986	+	-100k	+	S	ring finger protein 20
**827**	UID898	chr4	NM_182999	49750172	49760570	49653159	49677986	+	-100k	+	S	ring finger protein 20
**828**	UID899	chr4	NM_001162865	51317589	51327987	51237777	51251028	+	-100k	+	S	cylicin, basic protein of sperm head
**829**	UID901	chr4	NM_139309	53687574	53698015	53735281	53784371	+	+50k	+	AS	fukutin
**830**	UID901	chr4	NM_176966	53687574	53698015	53652654	53672719	+	-50k	+	S	cystatin and DUF19 domain-containing protein 1
**831**	UID906	chr4	NM_021297	66258684	66269125	66314171	66329157	+	+100k	+	AS	toll-like receptor 4 precursor
**832**	UID917	chr4	NM_031202	80281032	80291473	80305460	80322949	+	+50k	+	AS	tyrosinase-related protein 1 precursor
**833**	UID920	chr4	NM_001081096	85937186	85947621	85915928	86029534	+	-50k	-	AS	hypothetical protein LOC75811
**834**	UID927	chr4	NM_027089	94380760	94391201	94399310	94420828	+	+20k	-	AS	acrosome formation associated factor precursor
**835**	UID927	chr4	NM_177239	94380760	94391201	94434058	94471118	+	+100k	-	AS	myb-like, SWIRM and MPN domains 1
**836**	UID928	chr4	NM_001113412	94999593	95009991	95049524	95418957	+	+50k	+	AS	FGGY carbohydrate kinase domain containing
**837**	UID928	chr4	NM_029347	94999593	95009991	95071438	95313587	+	+100k	+	AS	FGGY carbohydrate kinase domain containing
**838**	UID929	chr4	NM_010425	99220394	99230835	99148316	99150689	+	-100k	+	S	forkhead box D3
**839**	UID929	chr4	NM_001081264	99220394	99230835	99207647	99255478	+	-20k	+	S	dolichyl pyrophosphate Man9GlcNAc2
**840**	UID929	chr4	NM_026348	99220394	99230835	99257419	99321136	+	+50k	-	AS	integrin beta 3 binding protein
**841**	UID930	chr4	NM_177732	102768241	102778682	102669649	102712816	+	-100k	-	AS	solute carrier family 35 (UDP-glucuronic
**842**	UID930	chr4	NM_025909	102768241	102778682	102811777	102864360	+	+50k	+	AS	OMA1 homolog, zinc metallopeptidase precursor
**843**	UID930	chr4	NM_001163494	102768241	102778682	102728379	102788795	+	-50k	-	AS	hypothetical protein LOC70941 isoform 2
**844**	UID930	chr4	NM_027612	102768241	102778682	102728379	102788795	+	-50k	-	AS	hypothetical protein LOC70941 isoform 1
**845**	UID934	chr4	NM_183300	108195547	108206033	108137190	108221808	+	-100k	-	AS	zinc finger, FYVE domain containing 9
**846**	UID934	chr4	NM_177045	108195547	108206033	108117887	108132054	+	-100k	+	S	coiled-coil and C2 domain containing 1B
**847**	UID935	chr4	NM_013876	109033031	109043429	108950788	108974437	+	-100k	-	AS	ring finger protein 11
**848**	UID935	chr4	NM_026291	109033031	109043429	109003306	109029229	+	-50k	-	AS	hypothetical protein LOC67646
**849**	UID936	chr4	NM_001038698	109681895	109692339	109701668	109785443	+	+20k	-	AS	ELAV-like 4 isoform b
**850**	UID936	chr4	NM_001163399	109681895	109692339	109701669	109784766	+	+20k	-	AS	ELAV-like 4 isoform d
**851**	UID936	chr4	NM_010488	109681895	109692339	109701669	109784766	+	+20k	-	AS	ELAV-like 4 isoform a
**852**	UID936	chr4	NM_001163397	109681895	109692339	109701669	109849843	+	+20k	-	AS	ELAV-like 4 isoform c
**853**	UID937	chr4	NM_177406	114832135	114842576	114796977	114830747	+	-50k	+	S	cytochrome P450, family 4, subfamily a
**854**	UID937	chr4	NM_172306	114832135	114842576	114909555	114936966	+	+100k	+	AS	cytochrome P450, family 4, subfamily a family

**855**	UID937	chr4	NM_001100183	114832135	114842576	114740015	114752489	+	-100k	+	S	cytochrome P450, family 4, subfamily a
**856**	UID939	chr4	NM_001039595	142848670	142859153	142778029	142784518	+	-100k	+	S	pramel family member
**857**	UID939	chr4	NM_001113736	142848670	142859153	142802640	142809158	+	-50k	+	S	pramel family member
**858**	UID939	chr4	NM_001113735	142848670	142859153	142802640	142809158	+	-50k	+	S	pramel family member
**859**	UID939	chr4	NM_001113736	142848670	142859153	142777998	142784520	+	-100k	+	S	pramel family member
**860**	UID939	chr4	NM_001113735	142848670	142859153	142818386	142824923	+	-50k	+	S	pramel family member
**861**	UID939	chr4	NM_001113735	142848670	142859153	142777998	142784520	+	-100k	+	S	pramel family member
**862**	UID939	chr4	NM_001007579	142848670	142859153	142834153	142840485	+	-20k	+	S	hypothetical protein LOC329986
**863**	UID939	chr4	NM_001126325	142848670	142859153	142920446	142923933	+	+100k	-	AS	hypothetical protein LOC277668
**864**	UID939	chr4	NM_001126324	142848670	142859153	142881689	142885282	+	+50k	+	AS	hypothetical protein LOC279185
**865**	UID939	chr4	NM_001113736	142848670	142859153	142818386	142824923	+	-50k	+	S	pramel family member
**866**	UID940	chr4	NM_001085419	143435801	143448866	143366566	143376788	+	-100k	+	S	hypothetical protein LOC100038764
**867**	UID940	chr4	NM_198661	143435801	143448866	143457416	143463621	+	+50k	+	AS	oogenesin 2
**868**	UID940	chr4	NM_201258	143435801	143448866	143424243	143429338	+	-20k	-	AS	oogenesin 3
**869**	UID941	chr4	NM_001081248	143864956	143875439	143880393	143890085	+	+20k	+	AS	predicted gene 13177
**870**	UID941	chr4	NM_001085504	143864956	143875439	143936623	143953055	+	+100k	-	AS	hypothetical protein LOC230890
**871**	UID941	chr4	NM_001085542	143864956	143875439	143821686	143831821	+	-50k	-	AS	hypothetical protein LOC627085
**872**	UID941	chr4	NM_198662	143864956	143875439	143786508	143796040	+	-100k	+	S	arylacetamide deacetylase-like
**873**	UID942	chr4	NM_001126316	144080479	144090920	144043890	144053270	+	-50k	-	AS	hypothetical protein LOC329993
**874**	UID942	chr4	NM_011303	144080479	144090920	144159763	144194896	+	+100k	+	AS	dehydrogenase/reductase (SDR family) member 3
**875**	UID943	chr4	NM_001081248	143912016	143922499	143880393	143890085	-	-50k	+	AS	predicted gene 13177
**876**	UID943	chr4	NM_001085504	143912016	143922499	143936623	143953055	-	+50k	-	S	hypothetical protein LOC230890
**877**	UID943	chr4	NM_001085542	143912016	143922499	143821686	143831821	-	-100k	-	AS	hypothetical protein LOC627085
**878**	UID943	chr4	NM_001085536	143912016	143922499	143969877	143988091	-	+100k	-	S	hypothetical protein LOC546849
**879**	UID944	chr4	NM_001039595	142793390	142803831	142778029	142784518	-	-20k	+	AS	pramel family member
**880**	UID944	chr4	NM_001113736	142793390	142803831	142802640	142809158	-	+10k	+	AS	pramel family member
**881**	UID944	chr4	NM_001113735	142793390	142803831	142802640	142809158	-	+10k	+	AS	pramel family member
**882**	UID944	chr4	NM_173773	142793390	142803831	142703850	142716989	-	-100k	-	AS	oogenesin 4
**883**	UID944	chr4	NM_001113736	142793390	142803831	142777998	142784520	-	-20k	+	AS	pramel family member
**884**	UID944	chr4	NM_001113735	142793390	142803831	142818386	142824923	-	+50k	+	AS	pramel family member
**885**	UID944	chr4	NM_001113735	142793390	142803831	142777998	142784520	-	-20k	+	AS	pramel family member
**886**	UID944	chr4	NM_001007579	142793390	142803831	142834153	142840485	-	+50k	+	AS	hypothetical protein LOC329986
**887**	UID944	chr4	NM_001126324	142793390	142803831	142881689	142885282	-	+100k	+	AS	hypothetical protein LOC279185
**888**	UID944	chr4	NM_001113736	142793390	142803831	142818386	142824923	-	+50k	+	AS	pramel family member
**889**	UID946	chr4	NM_023865	122207800	122218244	122191561	122207438	-	-20k	+	AS	palmitoyl-protein thioesterase-like protein
**890**	UID947	chr4	NM_001033791	120745156	120755639	120813950	120822552	-	+100k	-	S	hypothetical protein LOC545677
**891**	UID948	chr4	NM_177406	114841410	114851851	114796977	114830747	-	-50k	+	AS	cytochrome P450, family 4, subfamily a,
**892**	UID948	chr4	NM_172306	114841410	114851851	114909555	114936966	-	+100k	+	AS	cytochrome P450, family 4, subfamily a family
**893**	UID949	chr4	NM_177818	111426628	111437069	111470858	111481052	-	+50k	+	AS	selection and upkeep of intraepithelial T cells
**894**	UID949	chr4	NM_001102662	111426628	111437069	111504200	111527470	-	+100k	+	AS	selection and upkeep of intraepithelial T cells
**895**	UID949	chr4	NM_001100466	111426628	111437069	111417486	111448288	-	-10k	+	AS	selection and upkeep of intraepithelial T cells
**896**	UID949	chr4	NM_001142775	111426628	111437069	111470858	111486151	-	+50k	+	AS	selection and upkeep of intraepithelial T cells
**897**	UID949	chr4	NM_145551	111426628	111437069	111373308	111400728	-	-100k	-	AS	solute carrier family 5 (sodium/glucose

**898**	UID950	chr4	NM_001048189	109966483	109976882	109895722	111155442	-	-100k	+	AS	carboxypeptidase 6, cytosolic isoform 2
**899**	UID950	chr4	NM_030231	109966483	109976882	109895722	111155442	-	-100k	+	AS	carboxypeptidase 6, cytosolic isoform 1
**900**	UID951	chr4	NM_001048189	109812438	109822879	109895722	111155442	-	+100k	+	AS	carboxypeptidase 6, cytosolic isoform 2
**901**	UID951	chr4	NM_030231	109812438	109822879	109895722	111155442	-	+100k	+	AS	carboxypeptidase 6, cytosolic isoform 1
**902**	UID954	chr4	NM_025909	102870632	102881073	102811777	102864360	-	-100k	+	AS	OMA1 homolog, zinc metallopeptidase precursor
**903**	UID955	chr4	NM_146146	101250497	101260895	101215338	101313286	-	-50k	+	AS	leptin receptor isoform 1
**904**	UID955	chr4	NM_010704	101250497	101260895	101215338	101290190	-	-50k	+	AS	leptin receptor isoform 2
**905**	UID955	chr4	NM_001122899	101250497	101260895	101215338	101311599	-	-50k	+	AS	leptin receptor isoform 3
**906**	UID955	chr4	NM_001033790	101250497	101260895	101332900	101341954	-	+100k	-	S	hypothetical protein LOC545662
**907**	UID957	chr4	NM_007704	98133588	98144074	98038651	98211621	-	-100k	+	AS	InaD-like isoform 3
**908**	UID957	chr4	NM_001005787	98133588	98144074	98038651	98181028	-	-100k	+	AS	InaD-like isoform 4
**909**	UID957	chr4	NM_001081202	98133588	98144074	98218771	98230504	-	+100k	+	AS	LINE-1 type transposase domain containing 1
**910**	UID958	chr4	NM_013690	94282606	94293004	94231317	94366957	-	-100k	+	AS	endothelial-specific receptor tyrosine kinase
**911**	UID959	chr4	NM_026319	94190318	94200759	94106537	94185251	-	-100k	+	AS	coiled-coil domain containing 2
**912**	UID959	chr4	NM_013690	94190318	94200759	94231317	94366957	-	+50k	+	AS	endothelial-specific receptor tyrosine kinase
**913**	UID959	chr4	NM_175305	94190318	94200759	94128677	94142162	-	-100k	-	AS	leucine rich repeat containing 19 precursor
**914**	UID964	chr4	NM_010486	90674737	90685178	90742784	90864073	-	+100k	-	S	ELAV-like 2 isoform 2
**915**	UID964	chr4	NM_207686	90674737	90685178	90742784	90864073	-	+100k	-	S	ELAV-like 2 isoform 3
**916**	UID964	chr4	NM_207685	90674737	90685178	90742784	90892002	-	+100k	-	S	ELAV-like 2 isoform 1
**917**	UID969	chr4	NM_029931	87144730	87155171	87241155	87277545	-	+100k	-	S	myeloid/lymphoid or mixed lineage-leukemia
**918**	UID969	chr4	NM_027326	87144730	87155171	87241155	87504638	-	+100k	-	S	myeloid/lymphoid or mixed lineage-leukemia
**919**	UID973	chr4	NM_026821	80431408	80441849	80381916	80425532	-	-50k	+	AS	hypothetical protein LOC52829
**920**	UID976	chr4	NM_011211	75395190	75405631	75412469	76065530	-	+20k	-	S	protein tyrosine phosphatase, receptor type, D
**921**	UID976	chr4	NM_001014288	75395190	75405631	75412469	75604707	-	+20k	-	S	protein tyrosine phosphatase, receptor type, D
**922**	UID978	chr4	NM_025849	74818967	74829403	74748584	74749517	-	-100k	-	AS	hypothetical protein LOC66928
**923**	UID979	chr4	NM_011599	71559275	71569716	71603502	71687280	-	+50k	-	S	transducin-like enhancer protein 1
**924**	UID988	chr4	NM_001009550	61645156	61655597	61634192	61637202	-	-20k	-	AS	major urinary protein 26
**925**	UID988	chr4	NM_009554	61645156	61655597	61675903	61694907	-	+50k	-	S	zinc finger protein 37
**926**	UID988	chr4	NM_001039544	61645156	61655597	61569837	61573673	-	-100k	-	AS	major urinary protein 3 precursor
**927**	UID989	chr4	NM_001037127	58478672	58489113	58380015	58468357	-	-100k	+	AS	skeletal muscle receptor tyrosine kinase isoform
**928**	UID989	chr4	NM_010944	58478672	58489113	58380015	58468357	-	-100k	+	AS	skeletal muscle receptor tyrosine kinase isoform
**929**	UID989	chr4	NM_172989	58478672	58489113	58529305	58647347	-	+100k	-	S	lysophosphatidic acid receptor 1
**930**	UID989	chr4	NM_010336	58478672	58489113	58529305	58647545	-	+100k	-	S	lysophosphatidic acid receptor 1
**931**	UID989	chr4	NM_001037128	58478672	58489113	58380015	58468357	-	-100k	+	AS	skeletal muscle receptor tyrosine kinase isoform
**932**	UID989	chr4	NM_001037130	58478672	58489113	58380015	58468357	-	-100k	+	AS	skeletal muscle receptor tyrosine kinase isoform
**933**	UID989	chr4	NM_001037129	58478672	58489113	58380015	58468357	-	-100k	+	AS	skeletal muscle receptor tyrosine kinase isoform
**934**	UID990	chr4	NM_011660	58105023	58115464	58037426	58050465	-	-100k	-	AS	thioredoxin 1
**935**	UID990	chr4	NM_026132	58105023	58115464	58078082	58103178	-	-50k	-	AS	thioredoxin domain containing 8
**936**	UID990	chr4	NM_022814	58105023	58115464	58136849	58300650	-	+50k	-	S	polydom precursor
**937**	UID994	chr4	NM_133891	53406915	53417356	53461512	53643578	-	+100k	+	AS	solute carrier family 44, member 1 isoform A
**938**	UID994	chr4	NM_001159633	53406915	53417356	53461512	53571264	-	+100k	+	AS	solute carrier family 44, member 1 isoform B
**939**	UID995	chr4	NM_025623	53072211	53082652	53033023	53043159	-	-50k	+	AS	nipsnap homolog 3B
**940**	UID995	chr4	NM_013454	53072211	53082652	53051888	53180995	-	-50k	-	AS	ATP-binding cassette 1, sub-family A, member 1

**941**	UID995	chr4	NM_146607	53072211	53082652	52991728	52992667	-	-100k	+	AS	olfactory receptor 270
**942**	UID995	chr4	NM_028529	53072211	53082652	53010383	53021954	-	-100k	+	AS	nipsnap homolog 3A
**943**	UID1001	chr4	NM_172688	32220184	32230583	32292719	32352077	-	+100k	+	AS	mitogen-activated protein kinase kinase kinase 7
**944**	UID1001	chr4	NM_009316	32220184	32230583	32292719	32352077	-	+100k	+	AS	mitogen-activated protein kinase kinase kinase 7
**945**	UID1008	chr4	NM_008899	22625727	22636168	22577628	22578966	-	-50k	-	AS	POU domain, class 3, transcription factor 2
**946**	UID1010	chr4	NM_010281	20258493	20268891	20164802	20188862	-	-100k	+	AS	gamma-glutamyl hydrolase precursor
**947**	UID1010	chr4	NM_172987	20258493	20268891	20334687	20869503	-	+100k	-	S	Na+/K+ transporting ATPase interacting 3
**948**	UID1011	chr4	NM_028746	19670211	19680652	19745873	19769360	-	+100k	+	AS	aspartate/glutamate transporter 1
**949**	UID1012	chr4	NM_025476	19589881	19600279	19502213	19534079	-	-100k	+	AS	family with sequence similarity 82, member B
**950**	UID1012	chr4	NM_177327	19589881	19600279	19536819	19636140	-	-100k	-	AS	WW domain-containing protein 1
**951**	UID1017	chr4	NM_178617	14940366	14950762	14879392	15076278	-	-100k	-	AS	N-terminal EF-hand calcium binding protein 1
**952**	UID1020	chr4	NM_173746	12879660	12890099	12834004	12908618	-	-50k	+	AS	hypothetical protein LOC208820
**953**	UID1024	chr4	NM_013526	9848024	9858422	9771518	9789492	-	-100k	+	AS	growth differentiation factor 6 precursor
**954**	UID1024	chr4	NR_004448	9848024	9858422	9843111	9844544	-	-5k	-	AS	
**955**	UID1025	chr4	NM_001081417	8580494	8590935	8618067	8793956	-	+50k	+	AS	chromodomain helicase DNA binding protein 7
**956**	UID1026	chr4	NM_173426	5634808	5645252	5571325	5727091	-	-100k	+	AS	hypothetical protein LOC242297
**957**	UID1033	chr8	NM_007850	23064503	23074900	23121345	23122186	+	+100k	+	AS	alpha-defensin, 27 precursor
**958**	UID1033	chr8	NM_010039	23064503	23074900	23131341	23131513	+	+100k	+	AS	alpha-defensin, 28 precursor
**959**	UID1033	chr8	NM_001079933	23064503	23074900	23083727	23084427	+	+20k	+	AS	alpha-defensin, 26 precursor
**960**	UID1033	chr8	NM_183268	23064503	23074900	22974800	22975782	+	-100k	+	S	defensin-related cryptdin 20
**961**	UID1033	chr8	NR_003146	23064503	23074900	23160556	23161398	+	+100k	+	AS	
**962**	UID1036	chr8	NM_145841	39019481	39029964	38991070	40130025	+	-50k	-	AS	sarcoglycan zeta
**963**	UID1045	chr8	NM_001163564	69879866	69890264	69789205	69819553	+	-100k	+	S	nuclear assembly factor 1 homolog
**964**	UID1049	chr8	NM_145600	85706011	85716452	85653724	85664231	+	-100k	-	AS	zinc finger protein 330
**965**	UID1049	chr8	NM_177378	85706011	85716452	85753460	85974919	+	+50k	+	AS	ring finger protein 150 precursor
**966**	UID1053	chr8	NM_130457	115366814	115377257	115456019	115768684	+	+100k	+	AS	contactin associated protein-like 4 precursor
**967**	UID1054	chr8	NM_130457	115530296	115540737	115456019	115768684	-	-100k	+	AS	contactin associated protein-like 4 precursor
**968**	UID1063	chr8	NM_053200	95999314	96009712	96055199	96086932	-	+100k	-	S	carboxylesterase 3 precursor
**969**	UID1063	chr8	NM_133660	95999314	96009712	96090346	96118669	-	+100k	-	S	esterase 22 precursor
**970**	UID1063	chr8	NM_001013764	95999314	96009712	95909341	95937320	-	-100k	-	AS	hypothetical protein LOC244595
**971**	UID1063	chr8	NM_001081372	95999314	96009712	95945854	95969145	-	-100k	-	AS	predicted gene 5158
**972**	UID1063	chr8	NM_007954	95999314	96009712	95988145	96020411	-	-20k	-	AS	esterase 1 precursor
**973**	UID1064	chr8	NM_022428	95473608	95484091	95563529	95569208	-	+100k	+	AS	Iroquois related homeobox 6
**974**	UID1065	chr8	NM_026904	82654813	82665254	82607891	82673393	-	-50k	+	AS	anaphase promoting complex subunit 10
**975**	UID1065	chr8	NM_015751	82654813	82665254	82579513	82607812	-	-100k	-	AS	ATP-binding cassette, subfamily E, member 1
**976**	UID1066	chr8	NM_010442	78035261	78045205	77989689	77996664	-	-50k	+	AS	heme oxygenase (decyclizing) 1
**977**	UID1066	chr8	NM_008566	78035261	78045205	78005599	78024511	-	-50k	+	AS	minichromosome maintenance deficient 5, cell
**978**	UID1066	chr8	NM_029182	78035261	78045205	78110015	78120185	-	+100k	+	AS	RASD family, member 2 precursor
**979**	UID1071	chr8	NM_011922	65047602	65058043	64949662	65015260	-	-100k	-	AS	annexin A10 isoform 2
**980**	UID1071	chr8	NM_001136089	65047602	65058043	64949663	65015239	-	-100k	-	AS	annexin A10 isoform 1
**981**	UID1073	chr8	NM_010402	60282008	60292406	60213124	60216659	-	-100k	+	AS	heart and neural crest derivatives expressed
**982**	UID1073	chr8	NM_009136	60282008	60292406	60348064	60369727	-	+100k	+	AS	scrapie responsive gene 1 precursor
**983**	UID1073	chr8	NM_021788	60282008	60292406	60374843	60380002	-	+100k	-	S	sin3 associated polypeptide

**984**	UID1078	chr8	NM_026067	36966962	36977396	36933781	36964050	-	-50k	-	AS	three prime histone mRNA exonuclease 1
**985**	UID1078	chr8	NM_001081279	36966962	36977396	37056314	37147966	-	+100k	+	AS	malignant fibrous histiocytoma amplified
**986**	UID1081	chr8	NM_153135	30078747	30089144	30112652	30685572	-	+50k	-	S	unc-5 homolog D precursor
**987**	UID1087	chr5	NM_198620	8554395	8564836	8496341	8628958	+	-100k	-	AS	RUN domain containing 3B
**988**	UID1088	chr5	NM_011076	8655990	8666388	8666097	8754576	+	+20k	+	AS	ATP-binding cassette, subfamily B, member 1A
**989**	UID1089	chr5	NM_013657	17118115	17128556	17086639	17242091	+	-50k	+	S	semaphorin 3C precursor
**990**	UID1090	chr5	NM_001136060	27623925	27634366	27592870	28057268	+	-50k	+	S	dipeptidylpeptidase 6 isoform 1
**991**	UID1091	chr5	NM_020295	29504734	29515176	29560588	29709177	+	+100k	-	AS	limb region 1
**992**	UID1091	chr5	NM_021470	29504734	29515176	29528792	29556078	+	+50k	+	AS	ring finger protein 32
**993**	UID1099	chr5	NM_019468	62063969	62074410	62097079	62098714	+	+50k	+	AS	glucose-6-phosphate dehydrogenase 2
**994**	UID1103	chr5	NM_023784	69808086	69818527	69795812	69821767	+	-20k	-	AS	Yip1 domain family, member 7
**995**	UID1103	chr5	NM_172711	69808086	69818527	69836076	69852765	+	+50k	+	AS	GUF1 GTPase homolog
**996**	UID1103	chr5	NM_001038015	69808086	69818527	69854136	69871420	+	+50k	-	AS	glucosamine-6-phosphate deaminase 2
**997**	UID1105	chr5	NM_010252	71038255	71048738	71030181	71121752	+	-10k	-	AS	gamma-aminobutyric acid A receptor, gamma 1
**998**	UID1107	chr5	NM_001159518	78481390	78491831	78424016	78482820	+	-100k	-	AS	insulin-like growth factor binding protein 7
**999**	UID1107	chr5	NM_153798	78481390	78491831	78385258	78424102	+	-100k	+	S	DNA directed RNA polymerase II polypeptide B
**1000**	UID1107	chr5	NM_008048	78481390	78491831	78424016	78482820	+	-100k	-	AS	insulin-like growth factor binding protein 7
**1001**	UID1108	chr5	NR_027806	78608050	78618455	78585969	78586523	+	-50k	-	AS	
**1002**	UID1112	chr5	NM_001122668	96058812	96069246	96016171	96044532	+	-50k	+	S	hypothetical protein LOC665943
**1003**	UID1112	chr5	NM_198667	96058812	96069246	95972393	95976475	+	-100k	-	AS	hypothetical protein LOC381724
**1004**	UID1113	chr5	NM_172885	128162789	128173272	128072693	128747770	+	-100k	-	AS	transmembrane protein 132D precursor
**1005**	UID1114	chr5	NM_001105159	145732215	145742613	145812190	145838257	+	+100k	-	AS	cytochrome P450, family 3, subfamily a,
**1006**	UID1114	chr5	NM_017396	145732215	145742613	145812190	145838257	+	+100k	-	AS	cytochrome P450, family 3, subfamily a,
**1007**	UID1114	chr5	NM_007820	145732215	145742613	145689835	145723250	+	-50k	-	AS	cytochrome P450, family 3, subfamily a,
**1008**	UID1115	chr5	NM_001105159	145757139	145767537	145812190	145838257	+	+100k	-	AS	cytochrome P450, family 3, subfamily a,
**1009**	UID1115	chr5	NM_017396	145757139	145767537	145812190	145838257	+	+100k	-	AS	cytochrome P450, family 3, subfamily a,
**1010**	UID1115	chr5	NM_007820	145757139	145767537	145689835	145723250	+	-100k	-	AS	cytochrome P450, family 3, subfamily a,
**1011**	UID1116	chr5	NM_177879	141579361	141589759	141494007	142466265	-	-100k	+	AS	sidekick 1 precursor
**1012**	UID1117	chr5	NM_010294	97759015	97769456	97695462	97697310	-	-100k	-	AS	glycerol kinase 2
**1013**	UID1118	chr5	NM_144910	96253052	96263449	96317098	96402292	-	+100k	-	S	CCR4-NOT transcription complex, subunit 6-like
**1014**	UID1118	chr5	NM_178854	96253052	96263449	96316039	96402057	-	+100k	-	S	CCR4-NOT transcription complex, subunit 6-like
**1015**	UID1118	chr5	NM_018866	96253052	96263449	96197240	96201370	-	-100k	+	AS	chemokine (C-X-C motif) ligand 13 precursor
**1016**	UID1121	chr5	NM_007423	91609302	91619745	91565913	91584107	-	-50k	+	AS	alpha fetoprotein precursor
**1017**	UID1121	chr5	NM_009654	91609302	91619745	91536088	91551803	-	-100k	+	AS	albumin precursor
**1018**	UID1121	chr5	NM_028478	91609302	91619745	91678275	91715687	-	+100k	-	S	Ras association (RalGDS/AF-6) domain family
**1019**	UID1121	chr5	NM_145146	91609302	91619745	91594148	91628744	-	-20k	+	AS	afamin precursor
**1020**	UID1122	chr5	NM_145561	87436468	87446909	87377414	87447948	-	-100k	-	AS	transmembrane protease, serine 11d precursor
**1021**	UID1122	chr5	NM_001033233	87436468	87446909	87484970	87543551	-	+50k	-	S	transmembrane protease, serine 11a
**1022**	UID1129	chr5	NM_001018019	68377670	68388114	68310969	68445531	-	-100k	+	AS	glutaredoxin, cysteine rich 1
**1023**	UID1134	chr5	NM_181857	34357956	34368397	34324056	34486305	-	-50k	-	AS	DNA-directed DNA polymerase nu
**1024**	UID1134	chr5	NM_001001985	34357956	34368397	34312840	34322773	-	-50k	+	AS	N-acetyltransferase 8-like
**1025**	UID1134	chr5	NM_001033458	34357956	34368397	34300330	34301862	-	-100k	+	AS	hypothetical protein LOC381633
**1026**	UID1135	chr5	NM_001033457	29679291	29689732	29765453	29782956	-	+100k	+	AS	nucleolar protein with MIF4G domain 1-like

**1027**	UID1136	chr5	NM_001136060	27688675	27699158	27592870	28057268	-	-100k	+	AS	dipeptidylpeptidase 6 isoform 1
**1028**	UID1137	chr5	NM_001136060	27555017	27565416	27592870	28057268	-	+50k	+	AS	dipeptidylpeptidase 6 isoform 1
**1029**	UID1142	chr5	NM_011995	14551481	14561925	14520923	14869465	-	-50k	+	AS	piccolo isoform 1
**1030**	UID1142	chr5	NM_001110796	14551481	14561925	14520923	14802012	-	-50k	+	AS	piccolo isoform 2
**1031**	UID1144	chr5	NM_001163223	6741026	6751467	6775037	7350384	-	+50k	-	S	zinc finger protein 804B
**1032**	UID1146	chr5	NM_194462	3986547	3996988	3934191	4086210	-	-100k	+	AS	A kinase (PRKA) anchor protein (yotiao) 9
**1033**	UID1146	chr5	NM_001013023	3986547	3996988	3896586	3899939	-	-100k	-	AS	mitochondrial transcription termination factor
**1034**	UID1146	chr5	NM_172135	3986547	3996988	3896586	3899939	-	-100k	-	AS	mitochondrial transcription termination factor
**1035**	UID1150	chr17	NM_172827	17256444	17266885	17232340	17329107	+	-50k	-	AS	leucyl/cystinyl aminopeptidase
**1036**	UID1151	chr17	NM_001104575	22405278	22415676	22305847	22323833	+	-100k	+	S	vomeronasal receptor Vmn2r112
**1037**	UID1152	chr17	NM_008205	36312829	36323270	36248477	36250697	+	-100k	-	AS	histocompatibility 2, M region locus 9
**1038**	UID1152	chr17	NM_177636	36312829	36323270	36278060	36280250	+	-50k	-	AS	histocompatibility 2, M region locus 1
**1039**	UID1152	chr17	NM_177637	36312829	36323270	36380962	36384288	+	+100k	+	AS	histocompatibility 2, M region locus 10.5
**1040**	UID1153	chr17	NM_031863	40426043	40436484	40386538	40398035	+	-50k	-	AS	centromere protein Q
**1041**	UID1153	chr17	NM_008650	40426043	40436484	40398208	40424383	+	-50k	+	S	methylmalonyl-Coenzyme A mutase precursor
**1042**	UID1153	chr17	NM_001145060	40426043	40436484	40368224	40377834	+	-100k	-	AS	glycine N-acyltransferase-like protein 3
**1043**	UID1153	chr17	NM_027834	40426043	40436484	40338965	40344042	+	-100k	-	AS	hypothetical protein LOC71583
**1044**	UID1154	chr17	NM_001104582	55047852	55058293	55068358	55100690	+	+50k	-	AS	vomeronasal 2, receptor 118
**1045**	UID1155	chr17	NM_001081653	57381607	57392051	57454904	58095677	+	+100k	+	AS	contactin associated protein-like 5C precursor
**1046**	UID1156	chr17	NM_026497	58728252	58738690	58686731	58698653	+	-50k	-	AS	nudix-type motif 12
**1047**	UID1158	chr17	NM_009547	69226726	69237167	69288879	69295446	+	+100k	+	AS	zinc finger protein 161
**1048**	UID1158	chr17	NR_026848	69226726	69237167	69321348	69324092	+	+100k	+	AS	
**1049**	UID1163	chr17	NM_001112798	81254690	81265131	81281430	81646703	-	+50k	-	S	solute carrier family 8 (sodium/calcium
**1050**	UID1163	chr17	NM_011406	81254690	81265131	81281430	81646703	-	+50k	-	S	solute carrier family 8 (sodium/calcium
**1051**	UID1164	chr17	NM_009994	79548756	79559154	79615278	79623367	-	+100k	-	S	cytochrome P450, family 1, subfamily b,
**1052**	UID1164	chr17	NM_201361	79548756	79559154	79523225	79590478	-	-50k	+	AS	hypothetical protein LOC381110
**1053**	UID1169	chr17	NM_001104547	18305254	18315695	18286344	18302775	-	-20k	+	AS	vomeronasal 2, receptor 96
**1054**	UID1170	chr17	NM_001104539	17384279	17394678	17408558	17438785	-	+50k	+	AS	vomeronasal receptor Vmn2r90
**1055**	UID1178	chr9	NM_001011812	39190786	39201227	39144363	39145308	+	-50k	+	S	olfactory receptor 951
**1056**	UID1178	chr9	NM_146503	39190786	39201227	39176695	39177640	+	-20k	-	AS	olfactory receptor 952
**1057**	UID1178	chr9	NM_146331	39190786	39201227	39212003	39212948	+	+50k	+	AS	olfactory receptor 954
**1058**	UID1178	chr9	NM_207141	39190786	39201227	39220350	39221295	+	+50k	-	AS	olfactory receptor 955
**1059**	UID1178	chr9	NM_146830	39190786	39201227	39234774	39244559	+	+50k	-	AS	olfactory receptor 44
**1060**	UID1178	chr9	NM_146745	39190786	39201227	39261353	39262289	+	+100k	-	AS	olfactory receptor 957
**1061**	UID1179	chr9	NM_172444	59840373	59850817	59767724	59939141	+	-100k	-	AS	thrombospondin, type I, domain containing 4
**1062**	UID1179	chr9	NM_001040426	59840373	59850817	59767714	60309041	+	-100k	-	AS	thrombospondin, type I, domain containing 4
**1063**	UID1179	chr9	NM_013708	59840373	59850817	59740776	59748085	+	-100k	-	AS	nuclear receptor subfamily 2, group E, member 3
**1064**	UID1179	chr9	NM_001101600	59840373	59850817	59749299	59757049	+	-100k	+	S	hypothetical protein LOC665389
**1065**	UID1180	chr9	NM_001081153	73191249	73201647	73276555	73730699	+	+100k	-	AS	unc13 homolog 3
**1066**	UID1181	chr9	NM_144533	98057680	98068121	98105934	98220780	+	+50k	+	AS	nicotinamide mononucleotide adenylyltransferase
**1067**	UID1183	chr9	NM_175025	105189440	105199838	105269461	105353217	+	+100k	-	AS	calcium-transporting ATPase 2C1
**1068**	UID1183	chr9	NM_025651	105189440	105199838	105253621	105263561	+	+100k	+	AS	asteroid homolog 1 isoform 1
**1069**	UID1184	chr9	NM_026179	122143028	122153257	122200313	122230221	+	+100k	+	AS	abhydrolase domain containing 5

**1070**	UID1185	chr9	NM_015793	109101424	109111822	109128530	109145082	-	+50k	-	S	F-box and WD-40 domain protein 14
**1071**	UID1185	chr9	NM_177598	109101424	109111822	109036632	109053381	-	-100k	-	AS	F-box and WD-40 domain protein 13
**1072**	UID1185	chr9	NM_001008428	109101424	109111822	109074837	109092130	-	-50k	-	AS	F-box and WD-40 domain protein 20
**1073**	UID1187	chr9	NM_177909	94444379	94454777	94479260	95038800	-	+50k	+	AS	solute carrier family 9 (sodium/hydrogen
**1074**	UID1191	chr9	NM_175213	81726098	81736539	81660461	81824209	-	-100k	+	AS	hypothetical protein LOC75033
**1075**	UID1192	chr9	NM_009022	70987227	70997668	71014348	71094803	-	+50k	+	AS	aldehyde dehydrogenase 1A2
**1076**	UID1192	chr9	NM_022026	70987227	70997668	70909220	70961849	-	-100k	-	AS	aquaporin 9
**1077**	UID1194	chr9	NM_146869	38249418	38259862	38153446	38154400	-	-100k	+	AS	olfactory receptor 147
**1078**	UID1194	chr9	NM_001011806	38249418	38259862	38180854	38181790	-	-100k	+	AS	olfactory receptor 901
**1079**	UID1194	chr9	NM_146802	38249418	38259862	38199444	38200374	-	-50k	+	AS	olfactory receptor 902
**1080**	UID1194	chr9	NM_146801	38249418	38259862	38214613	38215546	-	-50k	+	AS	olfactory receptor 904
**1081**	UID1194	chr9	NM_146804	38249418	38259862	38223319	38224252	-	-50k	+	AS	olfactory receptor 905
**1082**	UID1194	chr9	NM_146803	38249418	38259862	38238601	38239537	-	-20k	+	AS	olfactory receptor 906
**1083**	UID1194	chr9	NM_146805	38249418	38259862	38249241	38250174	-	-5k	+	AS	olfactory receptor 907
**1084**	UID1194	chr9	NM_146872	38249418	38259862	38266604	38267536	-	+20k	+	AS	olfactory receptor 908
**1085**	UID1194	chr9	NM_146873	38249418	38259862	38274304	38275240	-	+50k	+	AS	olfactory receptor 909
**1086**	UID1194	chr9	NM_146811	38249418	38259862	38289467	38290400	-	+50k	+	AS	olfactory receptor 910
**1087**	UID1194	chr9	NM_146810	38249418	38259862	38330814	38332782	-	+100k	+	AS	olfactory receptor 912
**1088**	UID1194	chr9	NM_001011523	38249418	38259862	38344793	38345732	-	+100k	+	AS	olfactory receptor 913
**1089**	UID1195	chr9	NM_029299	27213386	27223826	27146266	27151156	-	-100k	+	AS	spermatogenesis associated 19 precursor
**1090**	UID1197	chr9	NM_146606	18540406	18550805	18505093	18506035	-	-50k	-	AS	olfactory receptor 24
**1091**	UID1197	chr9	NM_146605	18540406	18550805	18565755	18566694	-	+50k	-	S	olfactory receptor 828
**1092**	UID1197	chr9	NM_147067	18540406	18550805	18607028	18607994	-	+100k	+	AS	olfactory receptor 829
**1093**	UID1197	chr9	NM_146566	18540406	18550805	18625730	18626678	-	+100k	+	AS	olfactory receptor 830
**1094**	UID1201	chr7	NM_001111296	12691772	12702204	12633683	12673172	-	-100k	+	AS	bile-salt sulfotransferase 2A1
**1095**	UID1201	chr7	NM_009286	12691772	12702204	12633718	12673172	-	-100k	+	AS	sulfotransferase family 2A,
**1096**	UID1203	chr7	NM_207547	19312190	19322586	19296644	19297610	+	-20k	-	AS	vomeronasal 1 receptor, D21
**1097**	UID1204	chr7	NM_001034904	21531712	21542156	21618193	21630049	-	+100k	-	S	similar to serine/threonine kinase
**1098**	UID1204	chr7	NM_001034904	22754901	22765345	22841382	22853238	-	+100k	-	S	similar to serine/threonine kinase
**1099**	UID1205	chr7	NM_001034904	21531712	21542156	21618193	21630049	-	+100k	-	S	similar to serine/threonine kinase
**1100**	UID1205	chr7	NM_001034904	22754901	22765345	22841382	22853238	-	+100k	-	S	similar to serine/threonine kinase
**1101**	UID1207	chr7	NM_001105055	41615718	41626122	41610033	41645823	+	-10k	-	AS	vomeronasal receptor Vmn2r58
**1102**	UID1217	chr7	NM_172310	65595729	65606212	65523847	65571043	+	-100k	+	S	threonyl-tRNA synthetase-like 2
**1103**	UID1217	chr7	NM_026795	65595729	65606212	65572366	65580863	+	-50k	+	S	TM2 domain containing 3 isoform 1
**1104**	UID1217	chr7	NM_178056	65595729	65606212	65572366	65580863	+	-50k	+	S	TM2 domain containing 3 isoform 2
**1105**	UID1224	chr7	NM_017400	82084312	82094753	82048354	82183600	+	-50k	+	S	SH3-domain GRB2-like 3
**1106**	UID1226	chr7	NM_011661	87249819	87260302	87303165	87369172	+	+100k	-	AS	tyrosinase precursor
**1107**	UID1227	chr7	NM_001081414	87384637	87394872	87459537	88010439	+	+100k	+	AS	glutamate receptor, metabotropic 5 isoform a
**1108**	UID1227	chr7	NM_001143834	87384637	87394872	87459537	88010439	+	+100k	+	AS	glutamate receptor, metabotropic 5 isoform b
**1109**	UID1227	chr7	NM_011661	87384637	87394872	87303165	87369172	+	-100k	-	AS	tyrosinase precursor
**1110**	UID1229	chr7	NM_207556	103153871	103164269	103060423	103061362	+	-100k	+	S	olfactory receptor 592
**1111**	UID1229	chr7	NM_146380	103153871	103164269	103085682	103086666	+	-100k	+	S	olfactory receptor 593
**1112**	UID1229	chr7	NM_207143	103153871	103164269	103093540	103094476	+	-100k	+	S	olfactory receptor 594

**1113**	UID1229	chr7	NM_001001559	103153871	103164269	103123906	103126326	+	-50k	-	AS	deubiquitinating enzyme 2a
**1114**	UID1229	chr7	NM_001011845	103153871	103164269	103194233	103195181	+	+50k	+	AS	olfactory receptor 597
**1115**	UID1229	chr7	NM_001011793	103153871	103164269	103202308	103203268	+	+50k	+	AS	olfactory receptor 598
**1116**	UID1229	chr7	NM_146731	103153871	103164269	103211876	103212824	+	+100k	+	AS	olfactory receptor 599
**1117**	UID1229	chr7	NM_147046	103153871	103164269	103219802	103220747	+	+100k	-	AS	olfactory receptor 600
**1118**	UID1229	chr7	NM_146314	103153871	103164269	103232068	103233013	+	+100k	-	AS	olfactory receptor 601
**1119**	UID1230	chr7	NM_008219	103792643	103803126	103715458	103716983	+	-100k	-	AS	hemoglobin Z, beta-like embryonic chain
**1120**	UID1230	chr7	NM_008221	103792643	103803126	103725574	103727028	+	-100k	-	AS	hemoglobin Y, beta-like embryonic chain
**1121**	UID1230	chr7	NM_013616	103792643	103803126	103777277	103781357	+	-20k	+	S	olfactory receptor 65
**1122**	UID1230	chr7	NM_013618	103792643	103803126	103755126	103756062	+	-50k	-	AS	olfactory receptor 66
**1123**	UID1230	chr7	NM_013617	103792643	103803126	103766630	103768292	+	-50k	-	AS	olfactory receptor 64
**1124**	UID1230	chr7	NM_146959	103792643	103803126	103788882	103803732	+	-5k	+	S	olfactory receptor 631
**1125**	UID1230	chr7	NM_147119	103792643	103803126	103811202	103812156	+	+20k	+	AS	olfactory receptor 632
**1126**	UID1230	chr7	NM_146354	103792643	103803126	103820388	103821327	+	+50k	+	AS	olfactory receptor 633
**1127**	UID1230	chr7	NM_147118	103792643	103803126	103852996	103853962	+	+100k	+	AS	olfactory receptor 635
**1128**	UID1230	chr7	NM_147120	103792643	103803126	103877079	103878045	+	+100k	+	AS	olfactory receptor 638
**1129**	UID1230	chr7	NM_147084	103792643	103803126	103885570	103886521	+	+100k	-	AS	olfactory receptor 639
**1130**	UID1230	chr7	NM_001127686	103792643	103803126	103712944	103714342	+	-100k	-	AS	hypothetical protein LOC436003
**1131**	UID1231	chr7	NM_146329	103991103	104001545	103923228	103924173	+	-100k	-	AS	olfactory receptor 642
**1132**	UID1231	chr7	NM_146822	103991103	104001545	103895192	103896137	+	-100k	-	AS	olfactory receptor 640
**1133**	UID1231	chr7	NM_147072	103991103	104001545	103913618	103914557	+	-100k	+	S	olfactory receptor 641
**1134**	UID1231	chr7	NM_147077	103991103	104001545	103932476	103933421	+	-100k	-	AS	olfactory receptor 643
**1135**	UID1231	chr7	NM_147121	103991103	104001545	103941905	103942850	+	-50k	-	AS	olfactory receptor 644
**1136**	UID1231	chr7	NM_207144	103991103	104001545	103957951	103958899	+	-50k	-	AS	olfactory receptor 645
**1137**	UID1231	chr7	NM_147056	103991103	104001545	103980101	103981040	+	-20k	+	S	olfactory receptor 646
**1138**	UID1231	chr7	NM_198623	103991103	104001545	104014443	104017093	+	+50k	-	AS	ubiquilin 3
**1139**	UID1231	chr7	NM_198624	103991103	104001545	104022079	104024377	+	+50k	-	AS	ubiquilin-like
**1140**	UID1231	chr7	NM_172905	103991103	104001545	104026833	104038637	+	+50k	-	AS	hypothetical protein LOC244180
**1141**	UID1231	chr7	NM_146751	103991103	104001545	104053276	104054227	+	+100k	-	AS	olfactory receptor 648
**1142**	UID1231	chr7	NM_147055	103991103	104001545	104063087	104064026	+	+100k	-	AS	olfactory receptor 649
**1143**	UID1232	chr7	NM_007627	105221030	105231603	105299640	105310159	+	+100k	+	AS	cholecystokinin B receptor
**1144**	UID1232	chr7	NM_001011533	105221030	105231603	105161915	105162881	+	-100k	+	S	olfactory receptor 688
**1145**	UID1232	chr7	NM_146750	105221030	105231603	105187826	105188789	+	-50k	+	S	olfactory receptor 689
**1146**	UID1232	chr7	NM_147061	105221030	105231603	105210566	105211535	+	-20k	-	AS	olfactory receptor 691
**1147**	UID1232	chr7	NM_146355	105221030	105231603	105242148	105243138	+	+50k	+	AS	olfactory receptor 692
**1148**	UID1232	chr7	NM_001033317	105221030	105231603	105278388	105282559	+	+100k	+	AS	cyclic nucleotide gated channel alpha 4
**1149**	UID1232	chr7	NM_199009	105221030	105231603	105252430	105273832	+	+50k	-	AS	hypothetical protein LOC74349
**1150**	UID1232	chr7	NM_020290	105221030	105231603	105203037	105204099	+	-20k	-	AS	olfactory receptor 690
**1151**	UID1233	chr7	NM_146197	119292772	119303213	119352859	119387740	+	+100k	+	AS	acyl-CoA synthetase medium-chain family member
**1152**	UID1233	chr7	NM_025989	119292772	119303213	119233692	119250421	+	-100k	-	AS	zymogen granule membrane glycoprotein 2
**1153**	UID1233	chr7	NM_178758	119292772	119303213	119317413	119334509	+	+50k	+	AS	acyl-CoA synthetase medium-chain family member 5
**1154**	UID1233	chr7	NM_009470	119292772	119303213	119254015	119270404	+	-50k	-	AS	uromodulin precursor
**1155**	UID1233	chr7	NM_027943	119292772	119303213	119277735	119314631	+	-20k	-	AS	protein disulfide isomerase-like, testis

**1156**	UID1234	chr7	NR_024051	120294785	120305226	120212849	120335960	+	-100k	+	S	
**1157**	UID1234	chr7	NR_024324	120294785	120305226	120388773	120389623	+	+100k	+	AS	
**1158**	UID1238	chr7	NM_146329	103900405	103910847	103923228	103924173	-	+50k	-	S	olfactory receptor 642
**1159**	UID1238	chr7	NM_147119	103900405	103910847	103811202	103812156	-	-100k	+	AS	olfactory receptor 632
**1160**	UID1238	chr7	NM_146354	103900405	103910847	103820388	103821327	-	-100k	+	AS	olfactory receptor 633
**1161**	UID1238	chr7	NM_147118	103900405	103910847	103852996	103853962	-	-50k	+	AS	olfactory receptor 635
**1162**	UID1238	chr7	NM_147120	103900405	103910847	103877079	103878045	-	-50k	+	AS	olfactory receptor 638
**1163**	UID1238	chr7	NM_147084	103900405	103910847	103885570	103886521	-	-20k	-	AS	olfactory receptor 639
**1164**	UID1238	chr7	NM_146822	103900405	103910847	103895192	103896137	-	-10k	-	AS	olfactory receptor 640
**1165**	UID1238	chr7	NM_147072	103900405	103910847	103913618	103914557	-	+20k	+	AS	olfactory receptor 641
**1166**	UID1238	chr7	NM_147077	103900405	103910847	103932476	103933421	-	+50k	-	S	olfactory receptor 643
**1167**	UID1238	chr7	NM_147121	103900405	103910847	103941905	103942850	-	+50k	-	S	olfactory receptor 644
**1168**	UID1238	chr7	NM_207144	103900405	103910847	103957951	103958899	-	+100k	-	S	olfactory receptor 645
**1169**	UID1238	chr7	NM_147056	103900405	103910847	103980101	103981040	-	+100k	+	AS	olfactory receptor 646
**1170**	UID1239	chr7	NM_001162477	97015131	97025581	96956942	97184143	-	-100k	+	AS	growth factor receptor bound protein
**1171**	UID1239	chr7	NM_010248	97015131	97025581	96956942	97184143	-	-100k	+	AS	growth factor receptor bound protein
**1172**	UID1243	chr7	NM_001011767	86440050	86450491	86341173	86342166	-	-100k	+	AS	olfactory receptor 299
**1173**	UID1243	chr7	NM_001011751	86440050	86450491	86364311	86365310	-	-100k	-	AS	olfactory receptor 298
**1174**	UID1243	chr7	NM_146618	86440050	86450491	86402519	86403452	-	-50k	+	AS	olfactory receptor 297
**1175**	UID1243	chr7	NM_146851	86440050	86450491	86461037	86461967	-	+50k	+	AS	olfactory receptor 295
**1176**	UID1243	chr7	NM_001011750	86440050	86450491	86491396	86492404	-	+100k	-	S	olfactory receptor 294
**1177**	UID1243	chr7	NM_001011752	86440050	86450491	86539424	86540435	-	+100k	+	AS	olfactory receptor 293
**1178**	UID1245	chr7	NM_017400	82027555	82037996	82048354	82183600	-	+50k	+	AS	SH3-domain GRB2-like 3
**1179**	UID1248	chr7	NM_130880	63251429	63261873	63323721	63637977	-	+100k	+	AS	OTU domain containing 7
**1180**	UID1253	chr7	NM_001037906	49921334	49931774	49843370	50731308	-	-100k	+	AS	protein kinase C-binding protein NELL1
**1181**	UID1254	chr7	NM_153101	47372885	47383284	47331828	47357604	-	-50k	-	AS	MAS-related GPR, member A2
**1182**	UID1254	chr7	NM_153067	47372885	47383284	47456971	47469393	-	+100k	-	S	MAS-related GPR, member A3
**1183**	UID1255	chr7	NM_207212	33748588	33759028	33818309	33842028	-	+100k	-	S	WT1-interacting protein
**1184**	UID1255	chr7	NM_001099330	33748588	33759028	33804190	33809597	-	+100k	+	AS	hypothetical protein LOC100043868
**1185**	UID1255	chr7	NM_178308	33748588	33759028	33651756	33653745	-	-100k	-	AS	androgen binding protein gamma
**1186**	UID1255	chr7	NM_001100464	33748588	33759028	33720691	33722702	-	-50k	-	AS	androgen binding protein beta
**1187**	UID1255	chr7	NM_009596	33748588	33759028	33730326	33731641	-	-20k	+	AS	androgen binding protein alpha
**1188**	UID1256	chr7	NM_207212	33748588	33759028	33818309	33842028	-	+100k	-	S	WT1-interacting protein
**1189**	UID1256	chr7	NM_001099330	33748588	33759028	33804190	33809597	-	+100k	+	AS	hypothetical protein LOC100043868
**1190**	UID1256	chr7	NM_178308	33748588	33759028	33651756	33653745	-	-100k	-	AS	androgen binding protein gamma
**1191**	UID1256	chr7	NM_001100464	33748588	33759028	33720691	33722702	-	-50k	-	AS	androgen binding protein beta
**1192**	UID1256	chr7	NM_009596	33748588	33759028	33730326	33731641	-	-20k	+	AS	androgen binding protein alpha
**1193**	UID1258	chr10	NM_026405	10272033	10282431	10235228	10248397	+	-50k	-	AS	RAB32
**1194**	UID1262	chr10	NM_020565	33507938	33518379	33547137	33568891	+	+50k	+	AS	sulfotransferase family 3A, member 1
**1195**	UID1262	chr10	NM_001101452	33507938	33518379	33455839	33471531	+	-100k	-	AS	hypothetical protein LOC215895
**1196**	UID1262	chr10	NM_001162957	33507938	33518379	33594526	33605437	+	+100k	+	AS	radial spoke head 4 homolog A
**1197**	UID1263	chr10	NM_009925	34121489	34131972	34079396	34086501	+	-50k	+	S	procollagen, type X, alpha 1 precursor
**1198**	UID1263	chr10	NM_010237	34121489	34131972	34172815	34300642	+	+100k	+	AS	fyn-related kinase

**1199**	UID1263	chr10	NM_001159544	34121489	34131972	34172815	34300642	+	+100k	+	AS	fyn-related kinase
**1200**	UID1272	chr10	NM_011595	85661523	85674381	85730210	85779304	+	+100k	+	AS	tissue inhibitor of metalloproteinase 3
**1201**	UID1275	chr10	NM_001039354	106767694	106778135	106675940	106829253	+	-100k	+	S	lin 7 homolog a isoform 1
**1202**	UID1275	chr10	NM_001033223	106767694	106778135	106675940	106829253	+	-100k	+	S	lin 7 homolog a isoform 2
**1203**	UID1279	chr10	NM_178609	110248780	110259013	110149574	110191494	+	-100k	+	S	E2F transcription factor 7
**1204**	UID1279	chr10	NM_172554	110248780	110259013	110347146	110414118	+	+100k	-	AS	zinc finger, DHHC domain containing 17
**1205**	UID1279	chr10	NM_007792	110248780	110259013	110324285	110343624	+	+100k	+	AS	cysteine and glycine-rich protein 2
**1206**	UID1281	chr10	NM_001161855	115425219	115435657	115515774	115518027	+	+100k	-	AS	RIKEN cDNA 4933416C03
**1207**	UID1281	chr10	NM_011217	115425219	115435657	115422472	115679039	+	-5k	+	S	protein tyrosine phosphatase, receptor type, R
**1208**	UID1283	chr10	NM_001033474	112269085	112279526	112329537	112333136	-	+100k	-	S	hypothetical protein LOC382423
**1209**	UID1284	chr10	NM_029134	102636227	102646671	102644088	102666010	-	+10k	-	S	leucine-rich repeats and IQ motif containing 1
**1210**	UID1285	chr10	NM_146240	101855574	101866018	101941909	101976248	-	+100k	+	AS	Ras association (RalGDS/AF-6) domain family
**1211**	UID1285	chr10	NM_024435	101855574	101866018	101911443	101920106	-	+100k	-	S	neurotensin
**1212**	UID1285	chr10	NM_026243	101855574	101866018	101804124	101821154	-	-100k	+	AS	alpha-1,3-mannosyl-glycoprotein
**1213**	UID1299	chr10	NM_175448	33260680	33271078	33201749	33314016	-	-100k	-	AS	retinaldehyde binding protein 1-like 2
**1214**	UID1300	chr10	NM_001002268	14172804	14182458	14092782	14235234	-	-100k	-	AS	G protein-coupled receptor 126 precursor
**1215**	UID1301	chr10	NM_009538	12686625	12697066	12780985	12821892	-	+100k	+	AS	pleiomorphic adenoma gene-like 1
**1216**	UID1301	chr10	NM_175102	12686625	12697066	12698647	12699380	-	+20k	+	AS	splicing factor 3b, subunit 5
**1217**	UID1301	chr10	NM_001163590	12686625	12697066	12630178	12654457	-	-100k	-	AS	syntaxin 11
**1218**	UID1301	chr10	NM_001163591	12686625	12697066	12630178	12654457	-	-100k	-	AS	syntaxin 11
**1219**	UID1301	chr10	NM_029075	12686625	12697066	12630178	12654457	-	-100k	-	AS	syntaxin 11
**1220**	UID1305	chr12	NM_011859	9649776	9660216	9600449	9607507	+	-50k	+	S	odd-skipped related 1
**1221**	UID1306	chr12	NM_173417	11211054	11221495	11116544	11176716	+	-100k	-	AS	potassium voltage-gated channel,
**1222**	UID1306	chr12	NM_177331	11211054	11221495	11267268	11292130	+	+100k	-	AS	Gen homolog 1, endonuclease
**1223**	UID1306	chr12	NM_025695	11211054	11221495	11292228	11346128	+	+100k	+	AS	SMC6 protein
**1224**	UID1306	chr12	NM_019544	11211054	11221495	11234724	11235291	+	+50k	-	AS	mesogenin 1
**1225**	UID1310	chr12	NM_001003719	56548388	56558829	56521028	56739297	+	-50k	-	AS	GTPase activating RANGAP domain-like 1 isoform
**1226**	UID1310	chr12	NM_001112714	56548388	56558829	56521028	56739297	+	-50k	-	AS	GTPase activating RANGAP domain-like 1 isoform
**1227**	UID1310	chr12	NM_019994	56548388	56558829	56521028	56739297	+	-50k	-	AS	GTPase activating RANGAP domain-like 1 isoform
**1228**	UID1310	chr12	NM_020287	56548388	56558829	56517046	56520147	+	-50k	+	S	insulinoma-associated 2
**1229**	UID1310	chr12	NR_003959	56548388	56558829	56483264	56484839	+	-100k	+	S	
**1230**	UID1311	chr12	NM_009216	59035841	59046239	59129815	59134048	+	+100k	+	AS	somatostatin receptor 1
**1231**	UID1317	chr12	NM_012024	76295716	76306158	76369720	76515040	+	+100k	-	AS	epsilon isoform of regulatory subunit B56,
**1232**	UID1317	chr12	NM_023275	76295716	76306158	76227152	76320295	+	-100k	+	S	ras homolog gene family, member J precursor
**1233**	UID1317	chr12	NM_175644	76295716	76306158	76330559	76335621	+	+50k	-	AS	glycoprotein hormone beta 5 precursor
**1234**	UID1323	chr12	NM_173023	101845175	101855618	101805731	102026998	+	-50k	+	S	cation channel, sperm-associated, beta
**1235**	UID1325	chr12	NM_001163136	119956745	119967188	119887511	119911032	-	-100k	+	AS	metastasis associated in colon cancer 1
**1236**	UID1328	chr12	NM_001081169	112501491	112511974	112554490	112575381	-	+100k	+	AS	60 kDa lysophospholipase
**1237**	UID1328	chr12	NM_027360	112501491	112511974	112409183	112414785	-	-100k	-	AS	6.8 kDa mitochondrial proteolipid
**1238**	UID1328	chr12	NM_001097621	112501491	112511974	112594015	112629555	-	+100k	+	AS	kinesin family member 26A
**1239**	UID1328	chr12	NM_001127685	112501491	112511974	112427130	112428559	-	-100k	-	AS	hypothetical protein LOC217874
**1240**	UID1328	chr12	NM_029056	112501491	112511974	112419366	112516662	-	-100k	+	AS	tudor domain containing 9
**1241**	UID1329	chr12	NM_013747	102806141	102816582	102870959	102898957	-	+100k	+	AS	golgi autoantigen, golgin subfamily a, 5

**1242**	UID1329	chr12	NM_011175	102806141	102816582	102795147	102840747	-	-20k	-	AS	legumain precursor
**1243**	UID1330	chr12	NM_183186	99542039	99552480	99596143	99851124	-	+100k	-	S	checkpoint suppressor 1
**1244**	UID1331	chr12	NM_027076	96395131	96405565	96442445	96443053	-	+50k	-	S	hypothetical protein LOC69423
**1245**	UID1332	chr12	NM_201518	96006223	96016664	96088056	96181042	-	+100k	+	AS	fibronectin leucine rich transmembrane protein
**1246**	UID1342	chr12	NM_029760	53030411	53040852	53019365	53232579	-	-20k	+	AS	nucleotide binding protein-like
**1247**	UID1342	chr12	NM_008159	53030411	53040852	52944625	52949683	-	-100k	-	AS	G protein-coupled receptor 33
**1248**	UID1347	chr12	NM_015763	16647197	16657638	16561148	16615250	-	-100k	-	AS	lipin 1 isoform b
**1249**	UID1347	chr12	NM_172950	16647197	16657638	16561148	16615250	-	-100k	-	AS	lipin 1 isoform a
**1250**	UID1347	chr12	NM_015764	16647197	16657638	16696094	16826366	-	+50k	-	S	Greb1 protein
**1251**	UID1347	chr12	NM_008747	16647197	16657638	16678949	16685716	-	+50k	+	AS	neurotensin receptor 2
**1252**	UID1347	chr12	NM_001130412	16647197	16657638	16561148	16615250	-	-100k	-	AS	lipin 1 isoform a
**1253**	UID1352	chr6	NM_028990	13443291	13453732	13530688	13558063	+	+100k	-	AS	transmembrane protein 168
**1254**	UID1357	chr6	NM_028459	24620366	24630804	24563814	24614981	+	-100k	-	AS	Wiskott-Aldrich syndrome-like
**1255**	UID1357	chr6	NM_029848	24620366	24630804	24698376	24716529	+	+100k	+	AS	hyaluronoglucosaminidase 4
**1256**	UID1357	chr6	NM_053098	24620366	24630804	24547780	24555424	+	-100k	+	S	leiomodin 2 (cardiac)
**1257**	UID1357	chr6	NM_028920	24620366	24630804	24683254	24695462	+	+100k	+	AS	hyaluronoglucosaminidase 6
**1258**	UID1360	chr6	NM_001081678	28125131	28135531	28189940	28211611	+	+100k	-	AS	zinc finger protein 800
**1259**	UID1362	chr6	NM_172728	53407679	53418074	53502951	53625410	+	+100k	+	AS	cAMP responsive element binding protein 5
**1260**	UID1363	chr6	NM_053235	57214376	57224817	57139435	57140338	+	-100k	+	S	vomeronasal 1 receptor, C5
**1261**	UID1363	chr6	NM_053237	57214376	57224817	57163016	57163928	+	-100k	+	S	vomeronasal 1 receptor, C7
**1262**	UID1363	chr6	NM_053236	57214376	57224817	57187726	57188626	+	-50k	+	S	vomeronasal 1 receptor, C6
**1263**	UID1363	chr6	NM_134184	57214376	57224817	57252301	57253213	+	+50k	-	AS	vomeronasal 1 receptor, C29
**1264**	UID1363	chr6	NM_134171	57214376	57224817	57290044	57290956	+	+100k	-	AS	vomeronasal 1 receptor, C16
**1265**	UID1369	chr6	NM_019455	65142357	65152798	65046871	65074308	+	-100k	-	AS	prostaglandin D2 synthase, hematopoietic
**1266**	UID1370	chr6	NM_175524	65244728	65255170	65310871	65387728	+	+100k	+	AS	hypothetical protein LOC243407
**1267**	UID1372	chr6	NM_009042	78411784	78422215	78355491	78358175	+	-100k	+	S	regenerating islet-derived 1 precursor
**1268**	UID1372	chr6	NM_009043	78411784	78422215	78334663	78337606	+	-100k	+	S	regenerating islet-derived 2 precursor
**1269**	UID1372	chr6	NM_011260	78411784	78422215	78395776	78398383	+	-20k	-	AS	regenerating islet-derived 3 gamma precursor
**1270**	UID1379	chr6	NM_001014997	129820118	129830562	129732339	129741542	+	-100k	-	AS	killer cell lectin-like receptor subfamily H,
**1271**	UID1379	chr6	NM_008463	129820118	129830562	129864680	129878917	+	+50k	-	AS	killer cell lectin-like receptor, subfamily A,
**1272**	UID1379	chr6	NM_133203	129820118	129830562	129796846	129842363	+	-50k	-	AS	killer cell lectin-like receptor, subfamily A,
**1273**	UID1380	chr6	NM_177787	137985512	137995920	137947785	138037394	+	-50k	-	AS	solute carrier family 15, member 5
**1274**	UID1381	chr6	NM_011216	137190841	137201240	137216660	137427428	-	+50k	+	AS	protein tyrosine phosphatase, receptor type, O
**1275**	UID1383	chr6	NM_021899	122874054	122884540	122786129	122810980	-	-100k	+	AS	forkhead box J2
**1276**	UID1383	chr6	NM_009779	122874054	122884540	122812758	122821776	-	-100k	-	AS	complement component 3a receptor 1
**1277**	UID1383	chr6	NM_026267	122874054	122884540	122840175	122854560	-	-50k	+	AS	NECAP endocytosis associated 1
**1278**	UID1383	chr6	NM_199311	122874054	122884540	122887476	122900238	-	+20k	+	AS	C-type lectin domain family 4, member a1
**1279**	UID1383	chr6	NM_153197	122874054	122884540	122918262	122935493	-	+50k	+	AS	dendritic cell inhibitory receptor 3
**1280**	UID1383	chr6	NM_001005860	122874054	122884540	122955985	122989724	-	+100k	+	AS	C-type lectin domain family 4, member a4
**1281**	UID1386	chr6	NM_010585	108230670	108241153	108178872	108516887	-	-100k	+	AS	inositol 1,4,5-triphosphate receptor 1
**1282**	UID1389	chr6	NM_001102670	95174633	95185073	95083368	95095250	-	-100k	+	AS	T-cell activation kelch repeat protein isoform
**1283**	UID1389	chr6	NM_001008785	95174633	95185073	95083368	95095250	-	-100k	+	AS	T-cell activation kelch repeat protein isoform
**1284**	UID1390	chr6	NM_011684	89992005	90002446	89897153	89906011	-	-100k	-	AS	vomeronasal 1 receptor, A2

**1285**	UID1390	chr6	NM_053229	89992005	90002446	89941674	89942604	-	-100k	+	AS	vomeronasal 1 receptor, B8
**1286**	UID1390	chr6	NM_053219	89992005	90002446	89987391	89988324	-	-5k	+	AS	vomeronasal 1 receptor, A4
**1287**	UID1390	chr6	NM_053218	89992005	90002446	90001436	90002345	-	+10k	-	S	vomeronasal 1 receptor, A3
**1288**	UID1390	chr6	NM_053225	89992005	90002446	90072778	90073711	-	+100k	+	AS	vomeronasal 1 receptor, B1
**1289**	UID1390	chr6	NM_011683	89992005	90002446	90088146	90096149	-	+100k	+	AS	vomeronasal 1 receptor, A1
**1290**	UID1390	chr6	NM_011911	89992005	90002446	90037589	90038522	-	+50k	-	S	vomeronasal 1, receptor B2
**1291**	UID1392	chr6	NM_134169	66577103	66587501	66541228	66542146	-	-50k	-	AS	vomeronasal 1 receptor, C14
**1292**	UID1392	chr6	NM_134167	66577103	66587501	66608371	66609262	-	+50k	-	S	vomeronasal 1 receptor, C12
**1293**	UID1392	chr6	NM_134166	66577103	66587501	66645549	66646467	-	+100k	-	S	vomeronasal 1 receptor, C11
**1294**	UID1392	chr6	NM_134165	66577103	66587501	66660969	66661878	-	+100k	+	AS	vomeronasal 1 receptor, C10
**1295**	UID1392	chr6	NM_134170	66577103	66587501	66481760	66489286	-	-100k	-	AS	vomeronasal 1 receptor, C15
**1296**	UID1398	chr6	NM_134177	58353362	58363760	58364514	58365423	-	+20k	-	S	vomeronasal 1 receptor, C22
**1297**	UID1409	chr16	NM_001013761	26779374	26789815	26872467	26905207	+	+100k	-	AS	hypothetical protein LOC239789
**1298**	UID1410	chr16	NM_175548	41492452	41502893	41452673	42065545	+	-50k	+	S	limbic system-associated membrane protein
**1299**	UID1417	chr16	NM_173069	69772816	69783258	69739515	69746386	+	-50k	-	AS	spermatogenesis associated glutamate (E)-rich
**1300**	UID1425	chr16	NM_018778	88413474	88423915	88449684	88452042	+	+50k	-	AS	claudin 8
**1301**	UID1425	chr16	NM_181490	88413474	88423915	88394665	88395837	+	-20k	-	AS	claudin 17
**1302**	UID1425	chr16	NM_001163141	88413474	88423915	88499567	88501138	+	+100k	-	AS	keratin associated protein 24-1
**1303**	UID1426	chr16	NM_028621	89216033	89226474	89291885	89292633	+	+100k	-	AS	keratin associated protein 16-7
**1304**	UID1426	chr16	NM_010673	89216033	89226474	89308181	89308970	+	+100k	-	AS	keratin associated protein 6-2
**1305**	UID1427	chr16	NM_001113406	89522436	89532877	89459034	89460042	+	-100k	-	AS	keratin associated protein 11-1
**1306**	UID1429	chr16	NM_009621	85601720	85612118	85683023	85692311	-	+100k	-	S	a disintegrin-like and metalloprotease
**1307**	UID1433	chr16	NM_175549	73873550	73883991	73774985	74293592	-	-100k	-	AS	roundabout homolog 2
**1308**	UID1438	chr16	NM_007938	59639460	59649903	59595738	60547787	-	-50k	-	AS	Eph receptor A6
**1309**	UID1438	chr16	NM_019665	59639460	59649903	59555576	59581595	-	-100k	-	AS	ADP-ribosylation factor-like 6
**1310**	UID1439	chr16	NM_146397	59112794	59123235	59016410	59017334	-	-100k	-	AS	olfactory receptor 190
**1311**	UID1439	chr16	NM_001011807	59112794	59123235	59027807	59028737	-	-100k	-	AS	olfactory receptor 191
**1312**	UID1439	chr16	NM_207549	59112794	59123235	59040321	59041246	-	-100k	-	AS	olfactory receptor 192
**1313**	UID1439	chr16	NM_001011791	59112794	59123235	59051934	59052864	-	-100k	-	AS	olfactory receptor 193
**1314**	UID1439	chr16	NM_001005524	59112794	59123235	59061403	59062324	-	-100k	-	AS	olfactory receptor 194
**1315**	UID1439	chr16	NM_146998	59112794	59123235	59091107	59092034	-	-50k	+	AS	olfactory receptor 195
**1316**	UID1439	chr16	NM_146779	59112794	59123235	59109467	59110397	-	-5k	-	AS	olfactory receptor 196
**1317**	UID1439	chr16	NM_146484	59112794	59123235	59127811	59128737	-	+20k	-	S	olfactory receptor 197
**1318**	UID1439	chr16	NM_001011808	59112794	59123235	59143759	59144680	-	+50k	-	S	olfactory receptor 198
**1319**	UID1439	chr16	NM_146994	59112794	59123235	59210994	59211921	-	+100k	-	S	olfactory receptor 201
**1320**	UID1439	chr16	NM_207550	59112794	59123235	59157941	59158867	-	+50k	-	S	olfactory receptor 199
**1321**	UID1443	chr16	NM_026439	44968523	44978922	45013384	45047256	-	+50k	+	AS	steroid-sensitive protein 1 precursor
**1322**	UID1443	chr16	NM_028756	44968523	44978922	45058904	45078005	-	+100k	-	S	solute carrier family 35, member A5
**1323**	UID1448	chr14	NM_177624	12431524	12442007	12464142	12476415	+	+50k	+	AS	sentan
**1324**	UID1449	chr14	NM_177624	12484584	12495025	12464142	12476415	+	-50k	+	S	sentan
**1325**	UID1455	chr14	NM_177816	39750484	39760882	39724580	39801678	+	-50k	-	AS	SH2 domain containing 4B
**1326**	UID1455	chr14	NM_145928	39750484	39760882	39815156	39875520	+	+100k	-	AS	tetraspanin 14
**1327**	UID1456	chr14	NM_013542	55146899	55157340	55212927	55216331	+	+100k	-	AS	granzyme B precursor

**1328**	UID1456	chr14	NM_010373	55146899	55157340	55071689	55074696	+	-100k	-	AS	granzyme E precursor
**1329**	UID1456	chr14	NM_010372	55146899	55157340	55083638	55086664	+	-100k	-	AS	granzyme D
**1330**	UID1456	chr14	NM_010375	55146899	55157340	55110651	55113650	+	-50k	-	AS	granzyme G precursor
**1331**	UID1456	chr14	NM_153052	55146899	55157340	55119866	55128667	+	-50k	-	AS	granzyme N
**1332**	UID1456	chr14	NM_010374	55146899	55157340	55159333	55165478	+	+20k	-	AS	granzyme F precursor
**1333**	UID1456	chr14	NM_007800	55146899	55157340	55053951	55056645	+	-100k	-	AS	cathepsin G preproprotein
**1334**	UID1456	chr14	NM_010371	55146899	55157340	55185471	55188727	+	+50k	-	AS	granzyme C preproprotein
**1335**	UID1458	chr14	NM_027436	59826361	59836802	59738675	59857723	+	-100k	+	S	mitochondrial intermediate peptidase precursor
**1336**	UID1458	chr14	NM_013869	59826361	59836802	59918145	60000579	+	+100k	-	AS	tumor necrosis factor receptor superfamily,
**1337**	UID1469	chr14	NM_145467	122741399	122751840	122795319	123109259	+	+100k	+	AS	integrin, beta-like 1 precursor
**1338**	UID1488	chr14	NM_013542	55141405	55151846	55212927	55216331	-	+100k	-	S	granzyme B precursor
**1339**	UID1488	chr14	NM_010373	55141405	55151846	55071689	55074696	-	-100k	-	AS	granzyme E precursor
**1340**	UID1488	chr14	NM_010372	55141405	55151846	55083638	55086664	-	-100k	-	AS	granzyme D
**1341**	UID1488	chr14	NM_010375	55141405	55151846	55110651	55113650	-	-50k	-	AS	granzyme G precursor
**1342**	UID1488	chr14	NM_153052	55141405	55151846	55119866	55128667	-	-50k	-	AS	granzyme N
**1343**	UID1488	chr14	NM_010374	55141405	55151846	55159333	55165478	-	+20k	-	S	granzyme F precursor
**1344**	UID1488	chr14	NM_007800	55141405	55151846	55053951	55056645	-	-100k	-	AS	cathepsin G preproprotein
**1345**	UID1488	chr14	NM_010371	55141405	55151846	55185471	55188727	-	+50k	-	S	granzyme C preproprotein
**1346**	UID1489	chr14	NM_146494	48799343	48809784	48816208	48823892	-	+20k	-	S	olfactory receptor 722
**1347**	UID1489	chr14	NM_001011530	48799343	48809784	48850563	48851493	-	+100k	-	S	olfactory receptor 723
**1348**	UID1489	chr14	NM_146492	48799343	48809784	48882091	48883021	-	+100k	-	S	olfactory receptor 724
**1349**	UID1491	chr14	NM_007479	25530431	25540872	25471380	25490442	-	-100k	+	AS	ADP-ribosylation factor 4
**1350**	UID1491	chr14	NM_178668	25530431	25540872	25497300	25503030	-	-50k	-	AS	2'-phosphodiesterase
**1351**	UID1497	chrY	NM_011667	85740	96181	155155	180667	+	+100k	+	AS	ubiquitin-activating enzyme E1, Chr Y 1
**1352**	UID1497	chrY	NM_009570	85740	96181	61649	133852	+	-50k	-	AS	zinc finger protein 1, Y linked
**1353**	UID1498	chrY	NM_012011	332352	342793	347054	365037	+	+20k	+	AS	eukaryotic translation initiation factor 2,
**1354**	UID1498	chrY	NM_011419	332352	342793	234230	280254	+	-100k	+	S	jumonji, AT rich interactive domain 1D (Rbp2
**1355**	UID1498	chrY	NR_027507	332352	342793	392206	395311	+	+100k	-	AS	
**1356**	UID1499	chrY	NM_009571	1481822	1492263	1391082	1455317	-	-100k	-	AS	zinc finger protein 2, Y linked
