# Supplementary material for: The Impact of CpG Island on Defining Transcriptional Activation of the Mouse L1 Retrotransposable Elements
Source: PLoS One. 2010 Jun 29;5(6):e11353. doi: 10.1371/journal.pone.0011353 (PMC2894050; doi:10.1371/journal.pone.0011353)
Supplement: Table S6 — List of tissue-specific genes in the vicinity of L1 elements within 20-kb distance. (0.02 MB PDF) [file pone.0011353.s006.pdf]

Table 6: List of tissue-specific genes in the vicinity of L1 elements within 20-kb distance

Neighboring genes in sense orientation

No	LINE ID	Chr	LINE strand	Distance	RefSeq strand	CpG island	RefSeq ID	Cell/Tissue	RefSeq Definition	Biological function
1	UID1230	chr7	+	-5k	+	X	NM_146959		olfactory receptor 631 (Olfr631), 1482bp	
2	UID285	chr2	+	-5k	+	1 (5'UTR)	NM_001004174	testis, intestine	expressed sequence AA467197 (AA467197), 672bp	Normal mucosa of esophagus-specific gene 1 protein
3	UID1281	chr10	+	-5k	+	X	NM_011217	brain	protein tyrosine phosphatase, receptor type, R (Ptprr), 3430bp	Sequesters MAPKs
4	UID45	chr18	-	+10k	-	X	NM_177450	brain	carnosine dipeptidase 1	Catalysis of the hydrolysis of peptide bonds
5	UID1284	chr10	-	+10k	-	X	NM_029134	testicle	eucine-rich repeats and IQ motif containing 1 (Lrriq1), 2560bp	
6	UID790	chrX	-	+10k	-	X	NM_175179	kidney	Mus musculus family with sequence similarity 123, member B (Fam123b), mRNA	Involved in kidney development
7	UID866	chrX	-	+10k	-	X	NM_007807	Liver	cytochrome b-245, beta polypeptide (Cybb), 3242bp	Electron transport, Ion transport, Transport
8	UID1390	chr6	-	+10k	-	X	NM_053218	pancreas	vomeronasal 1 receptor, A3	Combining with a pheromone to initiate a change in cell activity
9	UID339	chr2	-	+20k	-	X	NM_146984		olfactory receptor 142 (Olfr142), 918bp	
10	UID63	chr18	-	+20k	-	2 (5'UTR)	NM_139143	brain, testis	solute carrier family 39 (metal ion transporter), member 6 (Slc39a6), 3287bp	zinc-influx transporter
11	UID312	chr2	-	+20k	-	X	NM_021527	wide	McKusick-Kaufman syndrome protein (Mkks), 2699bp	protein processing in limb, cardiac and reproductive system development
12	UID312	chr2	-	+20k	-	X	NM_001141946	wide	McKusick-Kaufman syndrome protein (Mkks), 2699bp	protein processing in limb, cardiac and reproductive system development
13	UID1398	chr6	-	+20k	-	X	NM_134177	Brain	vomeronasal 1 receptor, C22 (V1rc22), 909BP	G-protein coupled receptor protein signaling pathway
14	UID837	chrX	-	+20k	-	X	NM_001018063	lung	CAAX box 1 homolog B (human) (Cxx1b), 1246bp	
15	UID578	chr13	-	+20k	-	X	NM_134225	pancreas	vomeronasal 1 receptor, I9 (V1ri9), 960bp	G-protein coupled receptor protein signaling pathway
16	UID782	chrX	-	+20k	-	X	NM_009592	tymus	ATP-binding cassette, sub-family B (MDR/TAP),	the active transport of a substance across a membrane
17	UID1439	chr16	-	+20k	-	X	NM_146484		olfactory receptor 197 (Olfr197), 930bp	
18	UID1489	chr14	-	+20k	-	X	NM_146494		olfactory receptor 722 (Olfr722), 1655bp	
19	UID755	chrX	-	+20k	-	X	NM_019656	pancreas	tetraspanin 6 (Tspan6), 1741bp	Modulates exocytosis and secretion of hormones in the pancreas
20	UID976	chr4	-	+20k	-	X	NM_011211	Brain	protein tyrosine phosphatase, receptor type, D (Ptprd), transcript variant a, 7678bp	protein tyrosine phosphatase activity
21	UID976	chr4	-	+20k	-	X	NM_001014288	Brain	protein tyrosine phosphatase, receptor type, D (Ptprd), transcript variant b, 8360bp	protein tyrosine phosphatase activity
22	UID1488	chr14	-	+20k	-	X	NM_010374	Brain	granzyme F (Gzmf), 1027bp	target cell lysis in cell-mediated immune responses
23	UID343	chr2	-	+20k	-	X	NM_146753	muscle	olfactory receptor 1195 (Olfr1195), 927bp	
24	UID524	chr19	+	-20k	+	X	NM_146990		olfactory receptor 1494 (Olfr1494), 948bp	
25	UID705	chrX	+	-20k	+	1 (5'UTR)	NM_183320	testicle	hypothetical protein 4930481M05 (4930481M05), 3521bp	
26	UID704	chrX	+	-20k	+	1 (5'UTR)	NM_183320	testicle	hypothetical protein 4930481M05 (4930481M05), 3521bp	
27	UID705	chrX	+	-20k	+	1 (5'UTR)	NM_183320	testicle	hypothetical protein 4930481M05 (4930481M05), 3521bp	
28	UID704	chrX	+	-20k	+	1 (5'UTR)	NM_183320	testicle	hypothetical protein 4930481M05 (4930481M05), 3521bp	
29	UID717	chrX	+	-20k	+	X	NM_029199	Testis	hypothetical protein LOC75185, RIKEN cDNA 4930542N07 gene (4930542N07Rik)	
30	UID1230	chr7	+	-20k	+	X	NM_013616		olfactory receptor 64 (Olfr64), 1991bp	
31	UID939	chr4	+	-20k	+	X	NM_001007579	brain	hypothetical protein LOC329986, cDNA sequence BC080695 (BC080695), 2123bp	
32	UID929	chr4	+	-20k	+	X	NM_001081264		dolichyl pyrophosphate Man9GlcNAc2	
33	UID175	chr11	+	-20k	+	X	NM_146468		olfactory receptor 1391 (Olfr1391), 936bp	
34	UID268	chr2	+	-20k	+	X	NM_146365		olfactory receptor 1094 (Olfr1094), 993bp	
35	UID1231	chr7	+	-20k	+	X	NM_147056	brian	olfactory receptor 646 (Olfr646), 939bp	

Neighboring genes in antisense orientation

No	LINE ID	Chr	LINE strand	Distance	RefSeq strand	CpG island	RefSeq ID	Cell/tissue	Refseq Definition	Biological function
1	UID325	chr2	-	-5k	-	X	NM_029790	wide (brain)	methyltransferase 5 domain containing 1 (Mett5d1), 1892bp	methyltransferase activity
2	UID342	chr2	-	-5k	-	X	NM_146464		olfactory receptor 1196 (Olfr1196), 966bp	
3	UID547	chr19	-	-5k	-	X	NM_144785	Kidney	solute carrier family 22 (organic anion transporter), member 19 (Slc22a19), 1964bp	organic anion transport
4	UID1439	chr16	-	-5k	-	X	NM_146779		olfactory receptor 196 (Olfr196), 930bp	
5	UID487	chr1	-	-10k	-	X	NM_010778	Testis	CD46 antigen, complement regulatory protein (Cd46), 1214bp	the fusion of the spermatozoa with the oocyte during fertilization
6	UID745	chrX	-	-10k	-	1 (5'UTR)	NM_009121	kidney and color	spermidine/spermine N1-acetyl transferase 1 (Sat1), 1233bp	catalyzes the acetylation of polyamines
7	UID1238	chr7	-	-10k	-	X	NM_146822		olfactory receptor 640 (Olfr640), 945bp	
8	UID45	chr18	-	+10k	+	X	NM_177450	kidney	carnosine dipeptidase 1 (metallopeptidase M20 family) (Cndp1), 2814bp	proteolysis
9	UID198	chr11	-	-20k	-	X	NM_146878		olfactory receptor 30 (Olfr30), 948bp	
10	UID330	chr2	-	-20k	-	X	NM_175161	Testis	RIKEN cDNA 4931422A03 gene (4931422A03Rik), 1038bp	
11	UID340	chr2	-	-20k	-	X	NM_146985		olfactory receptor 1270 (Olfr1270), 915bp	
12	UID488	chr1	-	-20k	-	X	NM_146106	Kidney	lysophospholipase-like 1 (Lyplal1), 1442bp	hydrolase activity
13	UID578	chr13	-	-20k	-	X	NM_145845		vomeronasal 1 receptor, I1 (V1ri1), 903bp	G-protein coupled receptor protein signaling pathway
14	UID783	chrX	-	-20k	-	X	NM_031384	Testis	testis expressed gene 11 (Tex11), 3233bp	
15	UID811	chrX	-	-20k	-	X	NM_031493	Testis	X-linked lymphocyte-regulated 5C (Xlr5c), 540bp	
16	UID812	chrX	-	-20k	-	X	NM_019405	wide (brain)	centrin 2 (Cetn2), 1187bp, non Start codon (ORF2: N)	microtubule-organizing center structure and function
17	UID837	chrX	-	-20k	-	X	NM_001018063	lung	CAAX box 1 homolog A (human) (Cxx1a), 1208bp	
18	UID988	chr4	-	-20k	-	X	NM_001009550		bM64F17.3 (putative novel protein) (bM64F17.1), 1046bp	
19	UID1063	chr8	-	-20k	-	X	NM_007954	Liver, kidney	esterase 1 (Es1), 1840bp	the detoxification of xenobiotics and in the activation of ester and amide prodrugs
20	UID1168	chr17	-	-20k	-	X	NM_177825	pancrease	hypothetical protein A130033B22 (A130033B22), 2587bp	
21	UID1238	chr7	-	-20k	-	X	NM_147084		olfactory receptor 639 (Olfr639), 951bp	
22	UID1329	chr12	-	-20k	-	1 (5'UTR)	NM_011175	kidney	legumain (Lgmn), 1889bp	Has a strict specificity for hydrolysis of asparaginyl bonds
23	UID473	chr1	+	+20k	+	X	NM_145509		RIKEN cDNA 5430435G22 gene (5430435G22Rik), 2909bp	Protein transport (GTP and Rab family)
24	UID524	chr19	+	+20k	+	X	NM_146989		olfactory receptor 1496 (Olfr1496), 954bp	
25	UID556	chr13	+	+20k	+	X	NM_011455	placenta	serine (or cysteine) peptidase inhibitor, clade B, member 9g (Serpinb9g), 1919bp	serine-type endopeptidase inhibitor activity
26	UID1088	chr5	+	+20k	+	X	NM_011076	Intesitin	ATP-binding cassette, sub-family B (MDR/TAP), member 1A (Abcb1a), 4924bp	Mediates ATP-dependent export of organic anions and drugs from the cytoplasm
27	UID1230	chr7	+	+20k	+	X	NM_147119		olfactory receptor 632 (Olfr632), 954bp	
28	UID1498	chrY	+	+20k	+	X	NM_012011	prostate	eukaryotic translation initiation factor 2, subunit 3, structural gene Y-linked (Eif2s3y)	DNA, protein and zinc ion binding

X represents the absence of CpG island; 1(5'UTR) indicates the presence of CpG island at the 5'-UTR promoter region
Highlighted genes are orientated in sense directions within 5-kb distance of nearby L1 elements
