# Supplementary material for: The Impact of CpG Island on Defining Transcriptional Activation of the Mouse L1 Retrotransposable Elements
Source: PLoS One. 2010 Jun 29;5(6):e11353. doi: 10.1371/journal.pone.0011353 (PMC2894050; doi:10.1371/journal.pone.0011353)
Supplement: Table S7 — The mutation rates at the YY1 transcription binding sites in Tf subfamily. (0.01 MB PDF) [file pone.0011353.s007.pdf]

Table 7: The mutation rates at the YY1 transcription binding sites in Tf subfamily

YY1 binding sites

Mouse L1 with neighboring genes

		activity +++	CpG	activity ++	CpG	activity +	CpG		activity -	CpG	**Total**	CpG
	**Total**	**105**	13	**58**	3	**79**	6	**22**	**65**	**20**	**307**	**42**
	ratio (%)	**34.20**		**18.89**		**25.73**		**52.38**	**21.17**	**47.62**		
mutation of YY1	**+/+**	91	10	47	2	60	3	15	13	3	211	18
	ratio (%)	86.67		81.03		75.95		83.33	20.00	16.67	68.73	
mutation of YY1	**+/-**	14	3	11	1	19	3	7	52	17	96	24
	ratio (%)	13.33		18.97		24.05		29.17	80.00	70.83	31.27	

*** 17 L1s have one monomer**

*** 290 L1s have at least two monomers**

Mouse L1 without neighboring genes

		activity +++	CpG	activity ++	CpG	activity +	CpG		activity -	CpG	**Total**	CpG
	**Total**	**190**	16	**149**	5	**132**	12	**33**	**97**	**25**	**568**	**58**
	ratio (%)	**33.45**		**26.23**		**23.24**		**56.90**	**17.08**	**43.10**		
mutation of YY1	**+/+**	164	11	131	2	103	1	14	34	1	432	15
	ratio (%)	86.32		87.92		78.03		93.33	35.05	6.67	76.06	
mutation of YY1	**+/-**	26	5	18	3	29	11	19	63	24	136	43
	ratio (%)	13.68		12.08		21.97		44.19	64.95	55.81	23.94	

*** 39 L1s have one monomer**

*** 529 L1s have at least two monomers**
